# Modified Gegen Qinlian Decoction Regulates Treg/Th17 Balance to Ameliorate DSS-Induced Acute Experimental Colitis in Mice by Altering the Gut Microbiota

**DOI:** 10.3389/fphar.2021.756978

**Published:** 2021-11-04

**Authors:** Yifan Wang, Jiaqi Zhang, Lin Xu, Jing Ma, Mengxiong Lu, Jinxin Ma, Zhihong Liu, Fengyun Wang, Xudong Tang

**Affiliations:** ^1^ Department of Gastroenterology, Peking University Traditional Chinese Medicine Clinical Medical School (Xiyuan), Beijing, China; ^2^ Academy of Integration of Chinese and Western Medicine, Peking University Health Science Center, Beijing, China; ^3^ Department of Gastroenterology, Xiyuan Hospital of China Academy of Chinese Medical Sciences, Beijing, China; ^4^ China Academy of Chinese Medical Sciences, Beijing, China

**Keywords:** ulcerative colitis, modified gegen qinlian decoction, Treg/Th17 balance, gut microbiota, SCFAs, fecal microbiota transplantation

## Abstract

Inflammatory bowel disease (IBD) is characterized by chronic pathology associated with extensive intestinal microbial dysregulation and intestinal inflammation. Thus, efforts are underway to manipulate the gut microbiome to improve inflammatory pathology. Gegen Qinlian decoction (GQD), a traditional Chinese medicine prescription, has been widely utilized for treating diarrhea and ulcerative colitis (UC) for thousands of years. However, the underlying mechanism of its efficacy and whether its protective effect against colitis is mediated by the gut microbiota are poorly understood. In the present study, our data demonstrated that modified GQD (MGQD) administration significantly improved the pathological phenotypes and colonic inflammation challenged by DSS in mice, which were specifically manifested as reduced loss of body weight, shortening of colon length, DAI score, histological score and suppressed inflammatory response. 16S rRNA sequencing and targeted metabonomics analysis showed that MQGD altered the diversity and community landscape of the intestinal microbiota and the metabolic profiles. In particular, MQGD significantly boosted the abundance of the intestinal microbiota producing short-chain fatty acids (SCFAs), which are causally associated with promoting the development of Treg cells and suppressing the differentiation of pro-inflammatory Th17 cells. More importantly, transferring fecal microbiota from MGQD-treated or healthy controls exhibited equivalent alleviative effects on colitis mice. However, this protective effect could not be replicated in experiments of mice with depleted intestinal microbes through broad-spectrum antibiotic cocktails (ABX), further supporting the importance of SCFA-producing gut microbiota in the beneficial role of MGQD. In general, MGQD therapy has the potential to remodel the intestinal microbiome and reestablish immune homeostasis to ameliorate DSS-induced colitis.

## 1 Introduction

Ulcerative colitis (UC) is an inflammatory bowel disease (IBD) characterized by recurrent abdominal pain, diarrhea, and bloody purulent stool, and the lesion involves the colonic mucosa and submucosa (Ordás et al., 2012). The incidence of IBD is on the rise globally, and it has become a global public health challenge due to the difficulty of treatment, the increasing morbidity worldwide, and the heavy economic burden on patients (Ng et al., 2017; Coward et al., 2019). Although medical management, including 5-ASA, glucocorticoids, immunosuppressants, and biologics, has been exploited to induce and maintain remission, there is currently a lack of effective methods to prevent recurrence and sustain remission of intestinal inflammation. Therefore, more effective options need to be explored (Cheifetz et al., 2017; Ungaro et al., 2017; Lin et al., 2019).

The gut microbiota is increasingly defined as a core environmental factor that affects many aspects of host physiology, such as intestinal homeostasis, immune balance and colonization resistance to foreign pathogens. If or when cross-talk is disrupted, it can have a negative impact on the individual and lead to a variety of diseases, including IBD (Honda and Littman, 2016; Belkaid and Harrison, 2017; Britton et al., 2019; Sun et al., 2020; Lee and Chang, 2021). Remarkable discrepancies in the intestinal microbiota conformation and diversity of intestinal flora between IBD patients and healthy people have been described in multiple studies. Specifically, gut microbiota disturbance in IBD patients commonly manifests as a decrease in the abundance of beneficial symbiotic bacteria, an expansion of potential pathogens, and a reduction in the overall biodiversity of the microbial ecosystem (Ott et al., 2004; Sepehri et al., 2007; Hirano et al., 2018). Additionally, studies have found that the symptoms of UC patients or mice were relieved after receiving fecal flora transplantation from healthy donors (Moayyedi et al., 2015; Goyal et al., 2018; Burrello et al., 2019). In addition, increased microbic diversity in fecal suspensions from multiple donors makes it more effective than that of a single donor (Levy and Allegretti, 2019).

It is well known that the disturbance of the Treg/Th17 balance is involved in the pathogenesis of UC. Foxp3 Tregs are essential for immunomodulatory effects through IL-10 and TGF-*β* secretion. Th17 cells are involved in the occurrence and development of various autoimmune diseases, including IBD, mainly producing proinflammatory cytokines (such as IL-17A and IL-21) (Britton et al., 2019; Liu et al., 2020; Fan et al., 2021). Moreover, intestinal flora and microbiota-derived metabolites can influence Treg/Th17 immune balance (Khan et al., 2019; Ahlawat et al., 2021). Notably, the microbiota strongly inducing Th17 cells can aggravate colitis in mouse models, while short-chain fatty acids (SCFAs), metabolites originating from the intestinal microbiota, promote the differentiation of Treg cells (Smith et al., 2013; Zeng and Chi, 2015). However, during a period of intestinal flora disorder, due to the overexpansion of intestinal causative microorganisms and the decrease in intestinal flora diversity, this balance is disturbed, resulting in the Treg/Th17 axis tending toward Th17 cells, leading to the risk of developing intestinal inflammation (Kleinewietfeld and Hafler, 2013; Omenetti and Pizarro, 2015).

Recently, studies on IBD have proven that many intestinal microorganisms producing anti-inflammatory SCFAs, such as *Bifidobacterium fragilis* and *Firmicutes*, and SCFA concentrations in the feces of patients with IBD or in animal models of IBD are significantly reduced. Impairment of SCFAs production is believed to lead to intestinal and immune dysfunction and destruction of tissue integrity, thereby leading to the occurrence and progression of IBD (Arpaia et al., 2013; Gonçalves et al., 2018; Ji et al., 2019). Furthermore, supplementation with SFCAs-producing bacteria (e.g., *Firmicutes*) or direct addition of SCFAs can inhibit the secretion of proinflammatory cytokines in IBD (Gonçalves et al., 2018). Therefore, therapies aimed at reprogramming the gut microbiome, in particular the bacteria generating SCFAs, may be prospective treatment strategies for IBD.

The drugs most commonly applied to treat IBD, such as aminosalicylate and immunomodulators, have been shown to have adverse effects, such as influencing kidney function (Peng et al., 2019). As a result, there is a growing need for IBD treatment with alternative candidates derived from natural ingredients. Gegen Qinlian decoction (GQD), composed of *Pueraria montana* var. *lobata* (*Willd.*) *Maesen and S.M.Almeida ex Sanjappa & Predeep*, Scutellaria baicalensis Georgi, *Coptis chinensis* Franch. and *Glycyrrhiza uralensis* Fisch. *ex DC*., is a well-known classical TCM for treating damp heat syndrome. It has been widely used in treating colitis, chronic diarrhea and diabetes (Xu et al., 2020; Zhao et al., 2020). Previously, GQD and its active components have been shown to improve the UC model through a variety of pathways and mechanisms, such as regulating the IL-6/JAK2/Stat3 signaling pathway, Notch signaling pathway and gut microbiota (Zhao et al., 2020; Li et al., 2021; Zhao et al., 2021). However, the underlying mechanism by which GQD protects against IBD still needs to be further elucidated.

In this study, given that intestinal flora imbalance is a major etiological factor of IBD, we attempted to dissect the possible mechanism of modified GQD (MGQD) with the addition of ***Zingiber officinale Roscoe*** and *Talcum* under the guidance of clinical practice and TCM theory on DSS-induced experimental colitis in mice and determine the effect of intestinal microorganisms. In this study, we first determined the protective effect of MGQD administration on colonic inflammation. To confirm whether the improvement effect of MGQD on colon inflammation was mediated by intestinal flora, ABX cocktail-induced intestinal flora depletion and FMT experiments were conducted. Finally, the relationship between the Treg/Th17 balance and intestinal flora was also investigated.

## 2 Materials and Methods

### 2.1 Composition and Preparation of Modified GQD

MGQD is a Chinese herbal mixture composed of Pueraria montana var. lobata (Willd.) Maesen and S.M.Almeida ex Sanjappa and Predeep [Fabaceae; Puerarie Lobatae Radix] (24 g)*,* Scutellaria baicalensis Georgi [Lamiaceae; Scutellariae Radix] (9 g)*, Coptis chinensis* Franch. [Ranunculaceae; *Coptidis Rhizoma*] (9 g)*, Glycyrrhiza uralensis* Fisch. *ex DC*. [Fabaceae; *Glycyrrhiza Radix Et Rhizoma*] (6 g)*, Zingiber officinale Roscoe* [Zingiberaceae; *Zingiberis rhizoma praeparatum*] (9 g) and *Talcum* (9 g) at a ratio of 8:3:3:2:3:3 (w/w/w/w/w/w), which are the daily clinical doses for humans. Therefore, the total dosage of MGQD is 66 g. The equivalent dose of 60 kg for adults and mice was calculated according to the conversion coefficient of body surface area. Therefore, the MGQD was administered to mice at 5 g crude herbs/kg (low dose, equivalent to half of the dose of MGQD commonly used in clinical patients), 10 g crude herbs/kg (medium dose, equivalent to the dosage of MGQD commonly used in clinical patients) and 20 g crude herbs/kg (high dose, equivalent to twice the dosage of MGQD commonly used in clinical patients). According to the volume of intragastric administration of mice was 0.1 ml/10 g body weight, low, medium and high concentrations of MGQD extract were concentrated to 0.5 g/ml (0.5 g crude herbs per milliliter water), 1 g/ml (1 g crude herbs per milliliter water) and 2 g/ml (2 g crude herbs per milliliter water), respectively. All crude herbal medicines were purchased from Xiyuan Hospital, China Academy of Chinese Medical Sciences, and prepared by the Department of Pharmaceutical Preparation of Xiyuan Hospital in accordance with good manufacturing practices. The MGQD water extract was obtained according to the standard of TCM decoction method. 240 g Pueraria montana var. lobata (Willd.) Maesen and S.M.Almeida ex Sanjappa & Predeep, 90 g Scutellaria baicalensis Georgi, 90 g *Coptis chinensis* Franch., 60 g *Glycyrrhiza uralensis* Fisch. *ex DC*., 90 g *Zingiber officinale Roscoe* and 90 g *Talcum* were mixed and immersed in distilled water (1:8, w/v) for 1 h at room temperature, then *Talcum* was first decocted for 15 min, then all the herbs were decocted for 40 min, then filtered. After filtration, the residue was then boiled again for 30 min with an addition of 6 times volume of distilled water (1:6, w/v). After that, the extracted filtrates were combined and concentrated to 0.5, 1 and 2 g crude drug/mL, respectively. And the concentrated decoction was sealed and placed at 4°C for later use. The extraction rate of 21.261% was obtained by evaporation of the concentrate at 100°C. These doses used in this study were within the range of *in vivo* studies reported by others (Luo et al., 2019; Lin et al., 2020; Ma et al., 2021; Song et al., 2021).

### 2.2 Component Analysis of MGQD With HPLC

The high concentration of MGQD extract (2 g crude herbs/mL) was determined using an Agilent 1,200 series HPLC system (Agilent Technologies, Palo Alto, CA, United States) equipped with a G1315D DAD detector and an Agilent 5 TC-C18 column (250 × 4.6 mm, 5 μm). The mobile phase consisted of acetonitrile (A) and 0.1% phosphoric acid solution (B) with a gradient elution as follows: 0–3 min for 15% A, 85% B; 3–45 min for 15–35% A, 85%–65% B. The detection wavelength was set at 275 nm, the flow rate was 1.0 ml/min, the loading volume was 10 μL and the column temperature was maintained at 30°C. According to the retention time and peak area of the reference standard, a total of 5 components of MGQD were detected, namely, puerarin, baicalin, wogonin, berberine chloride, and palmatine chloride. HPLC results are shown in Supplementary Figure S1.

### 2.3 Experimental Animals

Male C57BL/6J mice (6–8 weeks old; weighing 18–22 g) were purchased from SPF Biotechnology Co., Ltd (Beijing, China). The animals were housed under specific pathogen-free (SPF) conditions (12 h light/dark cycle, 21 ± 2°C with a relative humidity of 45 ± 10%) with free access to autoclaved food and water in the experimental animal center of Xiyuan Hospital, China Academy of Chinese Medical Sciences. All procedures and experiments carried out here were approved by the Ethics Review Committee for Animal Experimentation of Xiyuan Hospital, China Academy of Chinese Medical Sciences (Approval NO. 2019XLC003-2).

### 2.3 Induction of Colitis and Treatment

**FIGURE 1:**
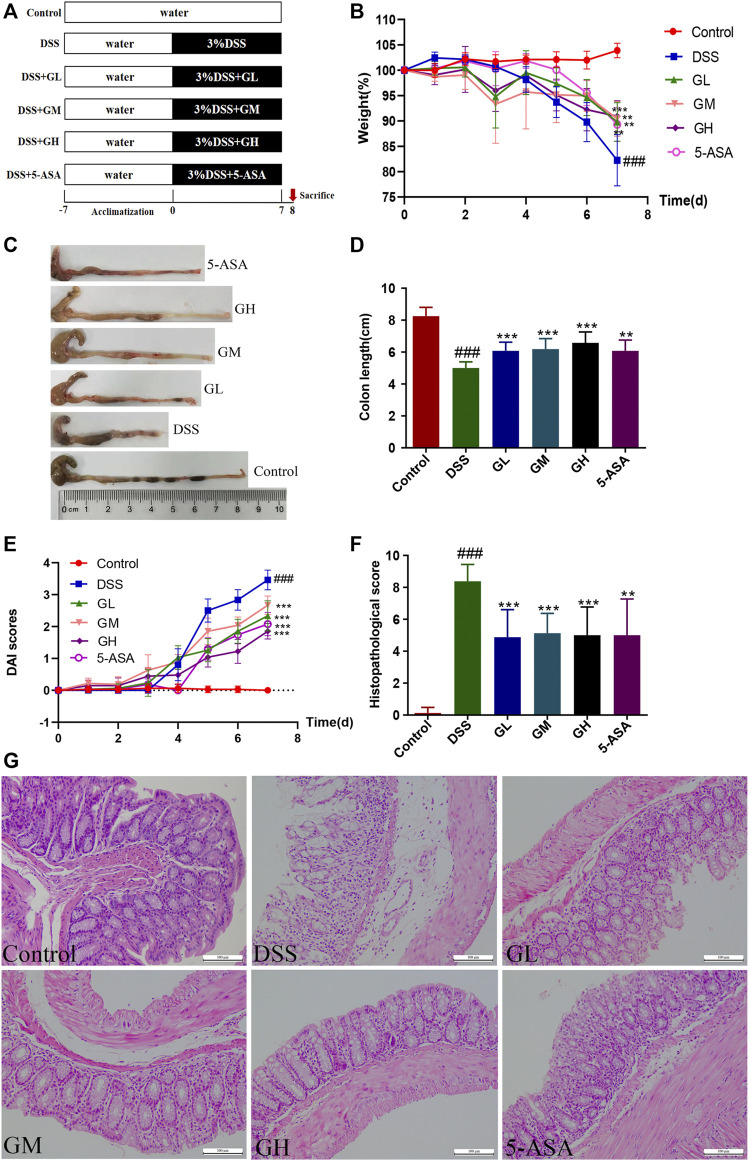
MGQD intervention ameliorated DSS-induced experimental colitis in mice. **(A)** The schematic diagram of UC model and drug treatment. **(B)** Percentage change of body weight. **(C)** Representative photograph of colons. **(D)** Statistics of colon length. **(E)** Disease activity index (DAI) score. **(F)** The histological scores of mouse colon morphology. **(G)** Representative microscopic pictures of H and E staining (amplification,×200). Results were shown as mean ± SD (n = 8–10). ###*p* < 0.001, vs control group; **p < 0.01, ***p < 0.001, vs DSS group.

The three experimental procedures were as follows:

Ⅰ To investigate the protective effect of MGQD against UC, 60 mice were stochastically divided into six groups (n = 10/group): a control group, DSS group, low-dose MGQD group (GL), medium-dose MGQD group (GM), high-dose MGQD group (GH), and 5-ASA group. Except for the normal group, all other five groups received 3% (w/v) DSS diluted in drinking water to induce acute experimental UC for 7 days (Wu et al., 2021). At the same time, each treatment group was given the corresponding dose of drugs once per day orally for seven consecutive days, namely, GL (5 g/kg), GM (10 g/kg), GH (20 g/kg) and 5-ASA (100 mg/kg) (Jing et al., 2019). Each mouse was given an intragastric dose of 10 μL/g body weight once per day. The MGQD dose used here was calculated based on the clinical dose of raw materials. Meanwhile, the normal group and DSS group were intragastrically given the same dosages of distilled water. On day 8, all mice were sacrificed under ether anesthesia, and the length of the colon was measured. The cecal contents, feces, colon tissues, spleens and mesenteric lymph nodes (MLNs) were collected for further analysis. The detailed experimental procedure is presented in Figure 1A.

**FIGURE 2:**
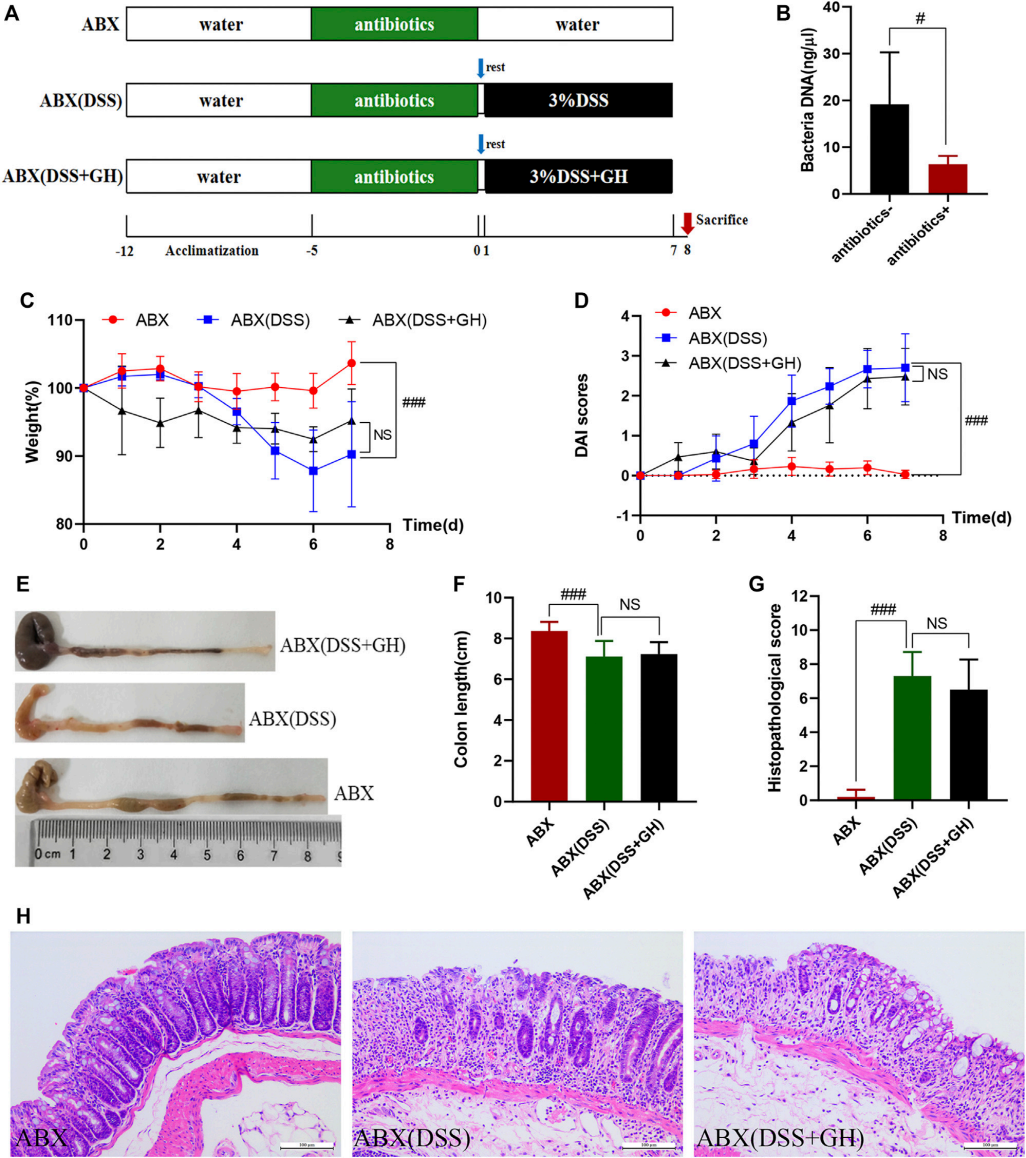
MGQD attenuated DSS-induced colonic inflammation in a gut microbiota dependent manner. **(A)** Experimental timeline. **(B)** Total DNA concentration from the gut microbiota in mice fecal samples. **(C)** Body weight change. **(D)** DAI score. **(E)** Representative photograph of colons. **(F)** Statistics of colon length. **(G)** The histological scores of mouse colon morphology. **(H)** Histological changes of the colon based on HE staining (magnification,×200). Data were expressed as mean ± SD (n = 10). #*p* < 0.05, ###*p* < 0.001 vs ABX group, NS means no statistical significance. ABX: a gut microbiota depletion group; ABX (DSS): an antibiotic-disposed UC model group; ABX (DSS + GH): an antibiotic-disposed UC model treatment group.

Ⅱ To investigate the role of intestinal flora in the improvement of MGQD in colitis, 30 mice were randomly divided into the ABX^+^DSS^−^GH^-^ group (ABX), ABX^+^DSS^+^GH^−^ group [ABX (DSS)], and ABX^+^DSS^+^GH^+^ group [ABX (DSS + GH)] with 10 mice in each group. All mice were given broad-spectrum antibiotic cocktails (1 g/L ampicillin, 1 g/L metronidazole, 0.5 g/L vancomycin and 0.5 g/L neomycin) for 5 days in advance to deplete the gut microbiota (Wu et al., 2020a). After 1 day of recovery, GH administration and UC induction were the same as those in Part Ⅰ. The mice were sacrificed under anesthesia on the 8th day. The experimental timeline is shown in Figure 2A.

**FIGURE 3:**
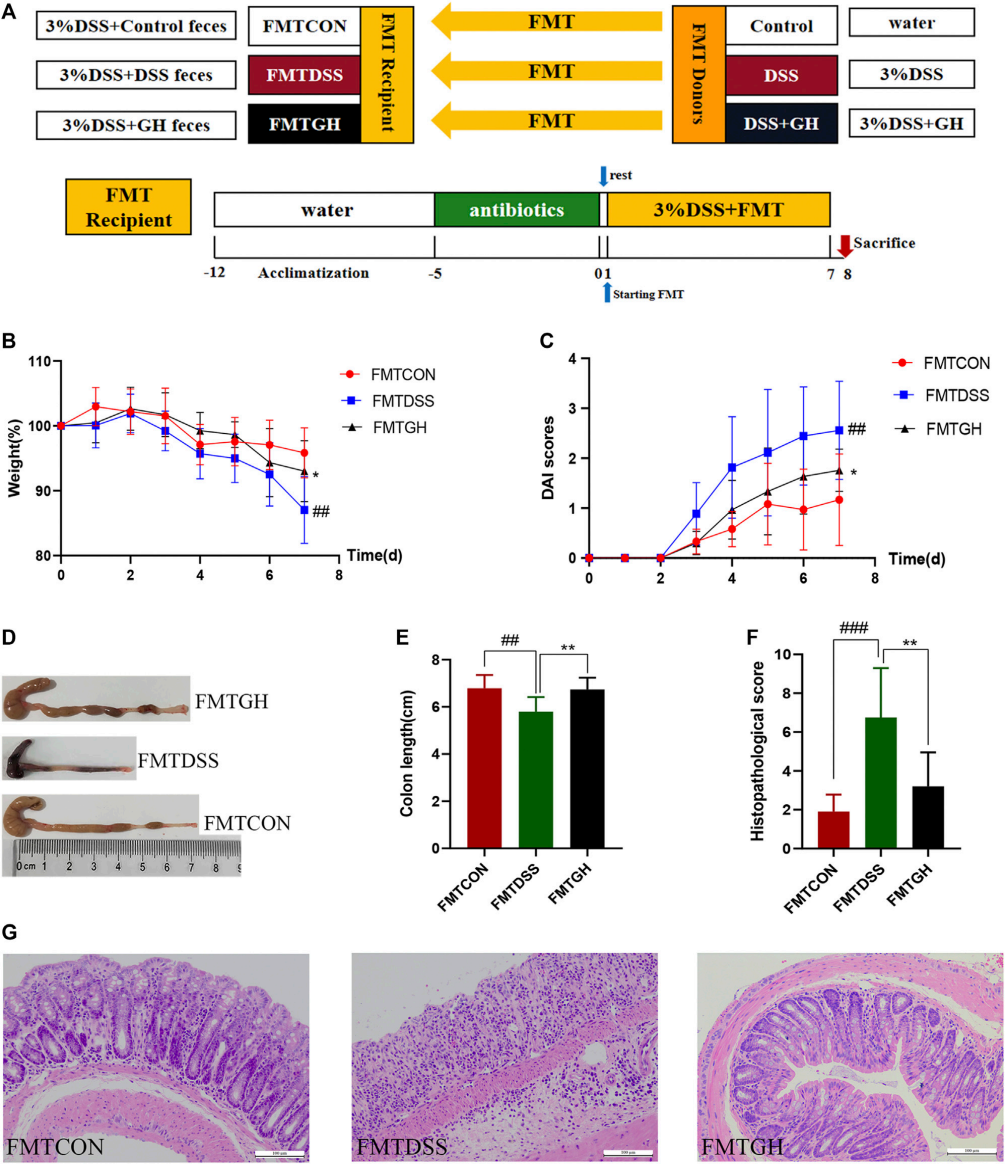
Transplantation of MGQD-altered gut microbiota recapitulated the effects of MGQD treatment on DSS-induced colitis. **(A)** Experimental design. **(B)** Body weight change. **(C)** DAI score. **(D)** Representative photograph of colons. **(E)** Statistics of colon length. **(F)** The histological scores of mouse colon morphology. **(G)** Histological changes of the colon based on HE staining (magnification,×200). Data were expressed as mean ± SD (n = 8–10). ##*p* < 0.01, ###*p* < 0.001, vs FMTCON group; *p < 0.05, **p < 0.01, vs FMTDSS group.

Ⅲ FMT was carried out according to a protocol described previously with minor modifications (Sun et al., 2020; Li et al., 2021). In brief, 24 donor mice were randomly assigned to the control group, DSS group, and DSS + GH group (n = 8/group). GH administration and induction of UC were described in the Part Ⅰ experiment. Fresh feces from each donor group were collected every day under aseptic conditions and sealed in a sterile container on ice. Weighing all collected feces and 100 mg of stools were resuspended in 1 ml of sterile PBS. After stirring, four layers of sterile gauze were used to filter the suspension. The solution was then violently mixed using a table vortex for 10 s, followed by centrifugation at 800 *g* for 3 min, and the supernatant was pooled and used as graft material within 10 min prior to gavage. Thirty recipient mice with depleted gut microbiota were divided into the FMTCON group, FMTDSS group and FMTGH group (n = 10/group) and orally administered the collected suspension from the donor CON group, DSS group and DSS + GH group for seven continuous days at a concentration of 0.1 ml/10 g body weight. Gut microbiota depletion preparation and UC induction were the same as in Part Ⅱ. The experimental timeline is shown in Figure 3A.

### 2.4 Disease Activity Index

The body weight, stool consistency, and hematochezia of each mouse were measured daily, and the DAI scores were calculated. For the parameters used for the calculation, refer to previous reports (Kihara et al., 2003).

### 2.5 Histological Analysis

The 1.5-cm distal colon was fixed in 10% neutral-buffered formalin, and 4-μm-thick sections were stained with hematoxylin and eosin (HE) according to standard procedures. Colonic pathology was evaluated based on a previously studied histological scoring system (Kihara et al., 2003).

### 2.6 Flow Cytometry Analysis

For Th17 cell staining, monocytes isolated from the spleens and MLNs were incubated with a cell stimulation cocktail (plus protein transport inhibitors) (Thermo Fisher Scientific, Waltham, MA, United States) at 37°C and 5% CO_2_ for 6–8 h. Surface staining was performed using FITC-anti-mouse CD4 (BioLegend Inc, San Diego, CA, United States). For intracellular staining, cells were fixed and permeabilized with fixation/permeabilization solution (BD Biosciences, CA, United States), and then antibodies against PE-anti-IL-17A antibody (BioLegend Inc, San Diego, CA, United States) were used. Similarly, for Treg cell staining, the cell surface markers FITC-anti-CD4^+^ (BioLegend Inc, San Diego, CA, United States) and PerCP-Cy5.5-anti-CD25^+^ (BioLegend Inc, San Diego, CA, United States) and the intranuclear staining marker AF647-anti-Foxp3+ (BioLegend Inc, San Diego, CA, United States) were used to specifically label the Treg cells. Finally, the stained cells were analyzed by flow cytometry.

### 2.7 Cytokine Determination

The levels of IL-17A, IL-21, IL-4 and TGF-*β* in the colonic tissues were detected using ELISA kits according to the manufacturer’s instructions (MultiSciences Biotech Co., Ltd, Hangzhou, China).

### 2.8 Investigation of Gut Microbiota by 16S rRNA Gene Sequencing

According to the E. Z.N.A.^®^ Soil DNA Kit (Omega Bio-Tek, Norcross, GA, United States) protocol, fecal genomic DNA was extracted from 0.1 g of frozen colon contents. A NanoDrop 2000 spectrophotometer (Thermo Fisher Scientific, Waltham, MA, United States) was used to measure the DNA concentration and purity, and DNA integrity was detected by 1% agarose gel electrophoresis. The V3-V4 region was amplified with the 16S rDNA gene universal primers 338 F (5′-ACT​CCT​ACG​GGA​GGC​AGC​AG-3′) and 806R (5′-GGACTACHVGGGTWTCTAAT-3′). The PCR amplification system was 20 μL, the PCR product was purified, and the concentration was adjusted. Sequencing was performed on an Illumina MiSeq PE300 system (MajorBio Co., Ltd, Shanghai, China).

### 2.9 Measurement of Short-Chain Fatty Acid in Cecal Contents

The SCFA standard used was a mixture of standard acetic acid, propanoic acid, isobutyric acid, butanoic acid, isovaleric acid, valeric acid, hexanoic acid and isohexanoic acid. All standards except propionic acid (Shanghai Ok-Kasei Co., Ltd.) were purchased from Sigma-Aldrich (Shanghai, China). The SCFAs were then extracted according to the manufacturer’s protocol (Majorbio Bio-Pharm Technology Co., Ltd, Shanghai, China). The analytical instrument used in this experiment to detect the extracted samples was an 8890B–5977 B GC/MSD (Agilent Technologies, Santa Clara, CA). Masshunter quantitative software (Agilent Inc, United States, version No. V10.0.707.0) was used to automatically identify and integrate the ion fragments of target short-chain fatty acids with default parameters, and manual inspection was also performed. The actual contents of SCFAs in the samples were calculated by calculating the detection concentration of each sample using the standard curve.

### 2.10 Bioinformatics and Statistical Analysis

Usearch (version 7.0) clustered operational taxon units (OTUs) with 97% identity and identified and deleted chimeric sequences. Alpha diversity index calculation were conducted using Mothur (version 1.30.2). Beta analysis was analyzed based on the Bray-Curtis, unweighted UniFrac and weighted UniFrac distance algorithms for principal coordinate analysis (PCoA). Microbial distribution was visualized using the R package (version 2.15.3) based on community composition information at the taxonomic level. The Kruskal-Wallis *H* test was used to detect the dominant bacterial community differences between the groups. Linear discriminant analysis (LDA) effect size (LEfSe) was applied to determine taxa, which can characterize each population (LDA score >3.6) to discover biomarkers (Liu et al., 2020).

### 2.11 Statistical Analysis

The significance level was determined by appropriate statistical analysis. GraphPad Prism 8.01 software was used for statistical analysis. The results were expressed as the means ± SD. Unpaired Student’s t-test was used for statistical analysis between the two groups. For more than two groups, one-way ANOVA and Bonferroni’s multiple comparison post test were used for statistical analysis. A *p* value <0.05 was recognized as statistically significant.

## 3 Results

### 3.1 Major Components in MGQD According to HPLC Analysis

The HPLC fingerprint of high concentration of MGQD extract (2 g crude herbs/mL) at the wavelength of 275 nm is shown in Supplementary Figure S1. According to the retention time and peak area of the reference standard, five components (puerarin, baicalin, wogonin, berberine chloride, and palmatine chloride) in MGQD were identified. After calculation, the contents of puerarin, baicalin, wogonin, berberine chloride and palmatine chloride were about 12.21, 17.18, 0.76, 10.25 and 2.67 mg/ml in high concentration of MGQD extract (2 g crude herbs/mL), respectively.

### 3.2 MGQD Intervention Mitigated DSS-Induced Mouse Colitis

In this study, the therapeutic efficacy of MGQD was evaluated in DSS-induced colitis mice. The experiment was carried out according to the timeline shown in Figure 1A. After 1 week of adaptive feeding, mice in the GL, GM, GH groups and 5-ASA groups were administered the corresponding treatment intragastrically from day 1 to day 7, and the process of DSS induction was synchronized. The normal group and the DSS model group were given the same dose of distilled water orally. Our results showed that in comparison with the normal group, the DSS group suffered significant weight loss and gross bloody stool. In contrast, the reduced body weight percentages of mice induced by DSS recovered significantly in the mice treated with GL, GM, GH, and 5-ASA (Figure 1B). In addition, the colon length in the DSS model group was significantly shortened compared to that in the control group. However, treatment with MGQD and 5-ASA significantly alleviated colon shortening (Figures 1C,D). The DAI score, an indicator of colitis severity calculated from weight loss, stool consistency, and gross bleeding, was dramatically elevated in the DSS group but significantly decreased in all dosages of MGQD and 5-ASA groups (Figure 1E). Furthermore, all doses of MGQD and 5-ASA remarkably improved histopathological changes in the colon, such as crypts destruction, loss of epithelial and goblet cells, and infiltration of a large number of inflammatory cells, and displayed significantly lower histopathological scores in comparison with the DSS model group (Figures 1F,G), indicating that both MGQD and 5-ASA showed profound effects on alleviating symptoms of colitis in the mice. Furthermore, the therapeutic effect of MGQD at the three doses was almost comparable to that of the 5-ASA group, and there was no remarkable discrepancy in the abovementioned parameters between the GL, GM, and GH groups.

### 3.3 MGQD Intervention Alleviated Colitis in a Gut Microbiota-Dependent Manner

To investigate whether the ameliorative effect of MGQD on DSS-induced colitis is related to intestinal microbiota, we utilized a mixture of quadruple antibiotics to eliminate the intestinal microflora of mice for 5 days in advance according to previous literature (Wu et al., 2020b; Li et al., 2021) (Figure 2A). After antibiotic exposure, significantly lower total DNA levels of the intestinal microbiota were observed in the feces of antibiotic-exposed mice, implying that most of the intestinal microbiota was essentially eliminated (Figure 2B). Interestingly, the mitigative effect of MGQD seemed to be abrogated when the intestinal flora was depleted in mice, as evidenced by the ABX (DSS) and ABX (DSS + GH) groups showing striking similarities in weight loss percentage (Figure 2C), DAI score (Figure 2D), colon length and gross lesions (Figures 2E, F), and colon histological changes (Figures 2G, H), indicating no significant difference between the two groups. The above results revealed that MGQD treatment ameliorated the inflammatory damage of the colonic mucosa resulting from DSS in a manner dependent on the gut microbiota.

### 3.4 Transplantation of MGQD-Altered Microbiota Recapitulated the Effects of MGQD Treatment on DSS-Induced Colitis

To further illustrate the protective effect of MGQD on DSS-induced colitis mediated by the gut microbiota, the FMT method was employed (Figure 3A). For better colonization, all recipient mice were given a cocktail of antibiotics prior to FMT to create conditions for intestinal microbiome depletion. Transferring fecal microbiota from control donors and GH-treated donors to DSS mice, namely, the FMTCON and FMTGH groups, both showed significantly attenuated colonic inflammation compared with mice receiving fecal microbiota from DSS-treated donors (FMTDSS), as manifested by the decreased weight loss percentage and DAI score and increased colon length. HE staining of the colon in the FMTGH and FMTCON groups both displayed less inflammatory cell infiltration, a relatively complete colon structure, less mucosal damage and a lower histological score than those in the FMTDSS group (Figures 3B–G). In conclusion, our results proved that the microbiota altered by MGQD effectively alleviated colitis induced by DSS, and fecal transplantation of MGQD-altered microbiota recreated the protective effect of MGQD, suggesting that the intestinal microbiota was responsible for the relieving effect of MGQD on intestinal inflammation.

### 3.5 MGQD Intervention Affected the Structure of the Gut Microbiota

**FIGURE 4:**
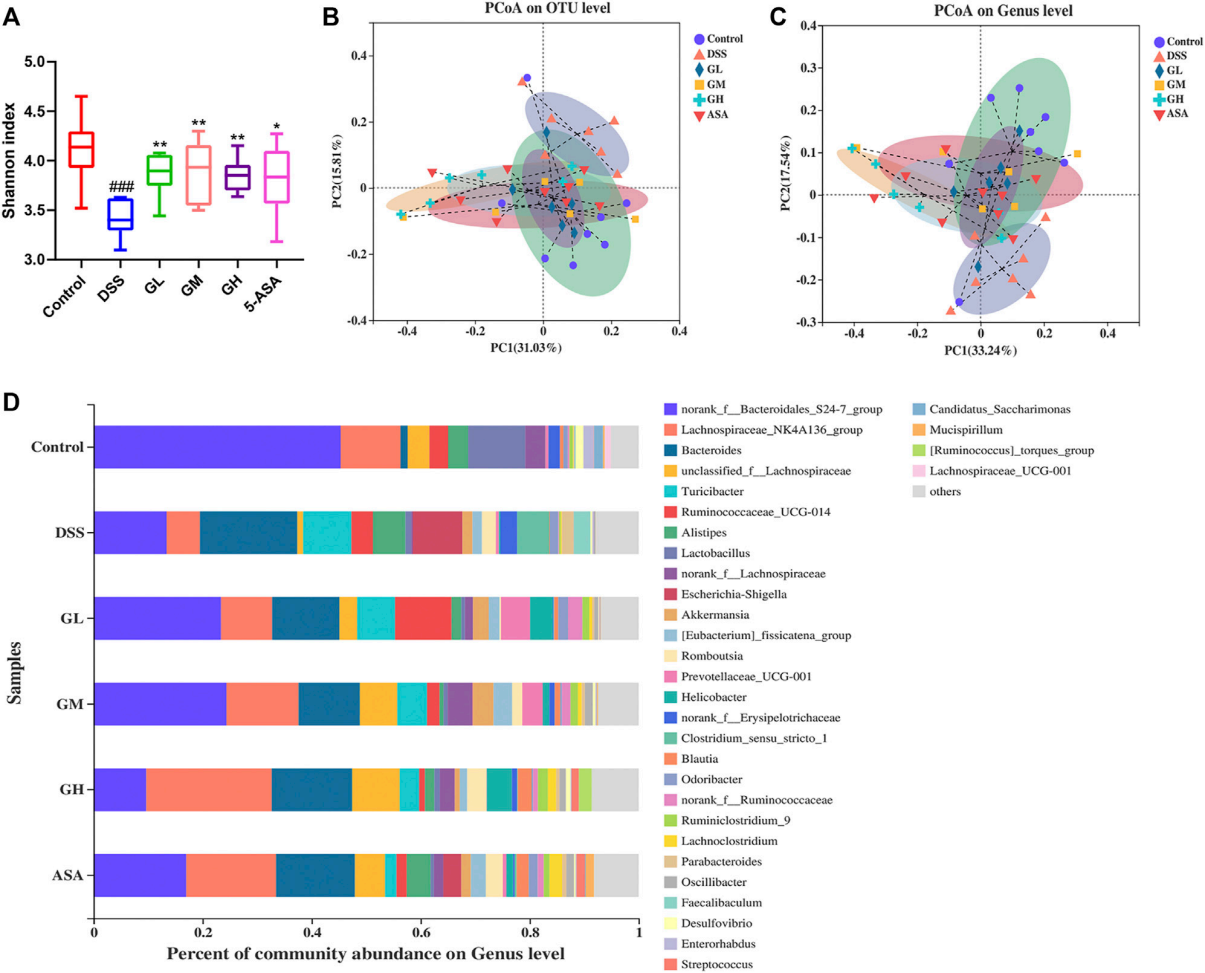
MGQD treatment significantly altered the gut microbiota in colitic mice. **(A)** Alpha diversity was estimated by using Shannon index. **(B)** and **(C)** Principal coordinate analysis (PCoA) based on Weighted-Unifrac distances among six groups at the OTU level and the genus level. Each symbol represents an individual mouse. **(D)** The bar plot of community composition on the Genus level. ###*p* < 0.001, vs control group; *p < 0.05, **p < 0.001, vs DSS group.

Given that dysregulation of the intestinal microbiota constitutes a central feature of IBD, we conducted 16S rRNA sequencing analysis in fecal bacterial DNA extracted from Con, DSS, GL, GM, GH and 5-ASA group mice to explore whether MGQD treatment changed the profile of the fecal microbiome challenged by DSS. We first noted that mice treated with all doses of MGQD and 5-ASA had a significantly higher Shannon index, an indicator of *α* diversity in the gut microbiome, compared with that of the DSS group (Figure 4A). Additionally, *β*-diversity analysis was used to assess the discrepancies between microbial communities. PCoA based on Weighted UniFrac distance showed that the DSS model group and the control group were aggregated separately, indicating that the composition and structure between the control group samples and that of the DSS model group were apparently different, whereas the distance of all treatment groups was closer to that of the control group in comparison with that of the DSS model group (Figures 4B, C). These results suggested that MGQD altered the diversity reduction of intestinal microorganisms and the structure of microbial communities challenged by DSS.

To further clarify the landscape of the bacterial community among the six groups, we then explored the impact of MQGD on the gut microbiome and its relative abundance. In terms of differences in species composition at the genus level, we found that some taxonomic microbiota exhibited a relatively higher abundance in the DSS-treated group, including *Bacteroides*, *Turicibacter, Escherichia-Shigella*, *Alistipes*, and *Clostridium_sensu_stricto_1,* while displaying a lower abundance of SCFA-producing bacteria, such as Lachnospiraceae*_NK4A136_group* and *Lactobacillus* than in the control group. Remarkably, all MGQD-treated and 5-ASA groups significantly reversed the relative abundances of *Escherichia-Shigella* and *Clostridium_Sensu_Stricto_1* to normal levels. Sequences that could not be classified were designated as “no rank” (Figure 4D). The above results were also confirmed by the Kruskal-Wallis *H* test (Supplementary Figure S2). The taxonomic composition of the six groups were also compared at the level of phylum, class, order and family (Supplementary Figures S3, 4).

**FIGURE 5:**
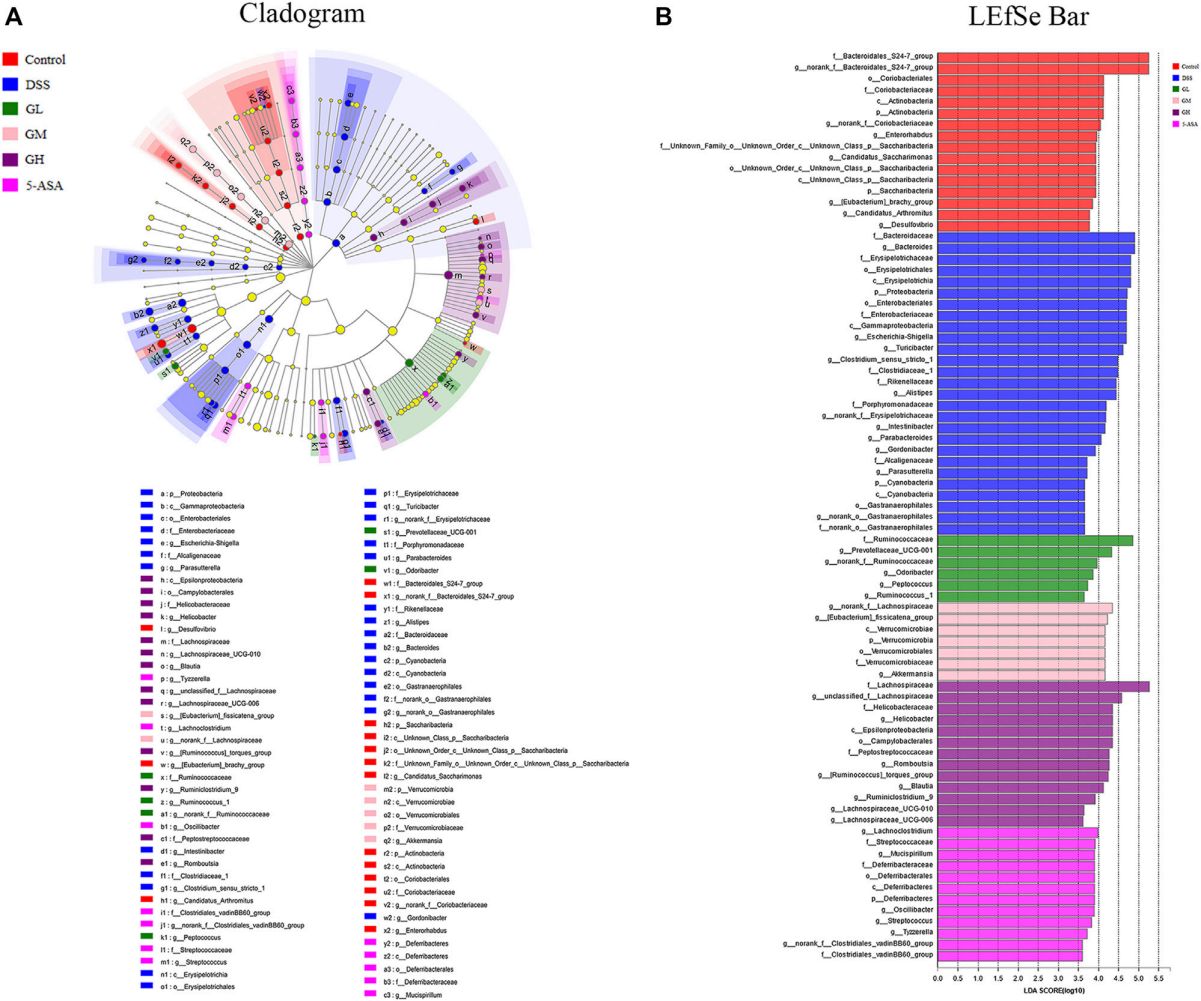
Screening of differentially abundant microbial taxa using linear discriminant analysis (LDA) coupled with effect size (LEfSe) analysis. **(A)** Taxonomic cladogram obtained from LEfSe analysis of 16S sequences. Yellow dots indicate no statistical significance. **(B)** Distribution histogram based on LDA, with a log LDA score above 3.6. The LDA histogram showed significant differences in species, which were all greater than the preset value. The color of the histogram represents each group, and the length of the histogram can express the degree of influence of species with significant differences between different groups.

Subsequently, LEfSe was performed to identify significant differences in the predominant bacterial community from phylum to genus among the six groups that may serve as a biomarker for colitis (LDA>3.6, *p* < 0.05). As shown in Figures 5A, B, Bacteroidaceae (from the family to the genus *Bacteroides*), Erysipelotrichaceae (from the class to the family) and *Escherichia coli-Shigella* (from the phylum *Proteobacteria* to the genus) were the core strains leading to the imbalance of intestinal flora in the DSS group. Nevertheless, the family Ruminococcaceae, the *g_norank_f__*Lachnospiraceae, and the family Lachnospiraceae exhibited comparative enrichment in the GL, GM, and GH groups, respectively, which might be related to the effect of anticolitis mediated by MGQD. In conclusion, the application of MGQD strikingly influenced the diversity and structure of the gut microflora.

### 3.6 Transfer of MGQD-Treated Fecal Microbiota Modulated the Composition of Intestinal Microbiota in DSS-Induced Mice

**FIGURE 6:**
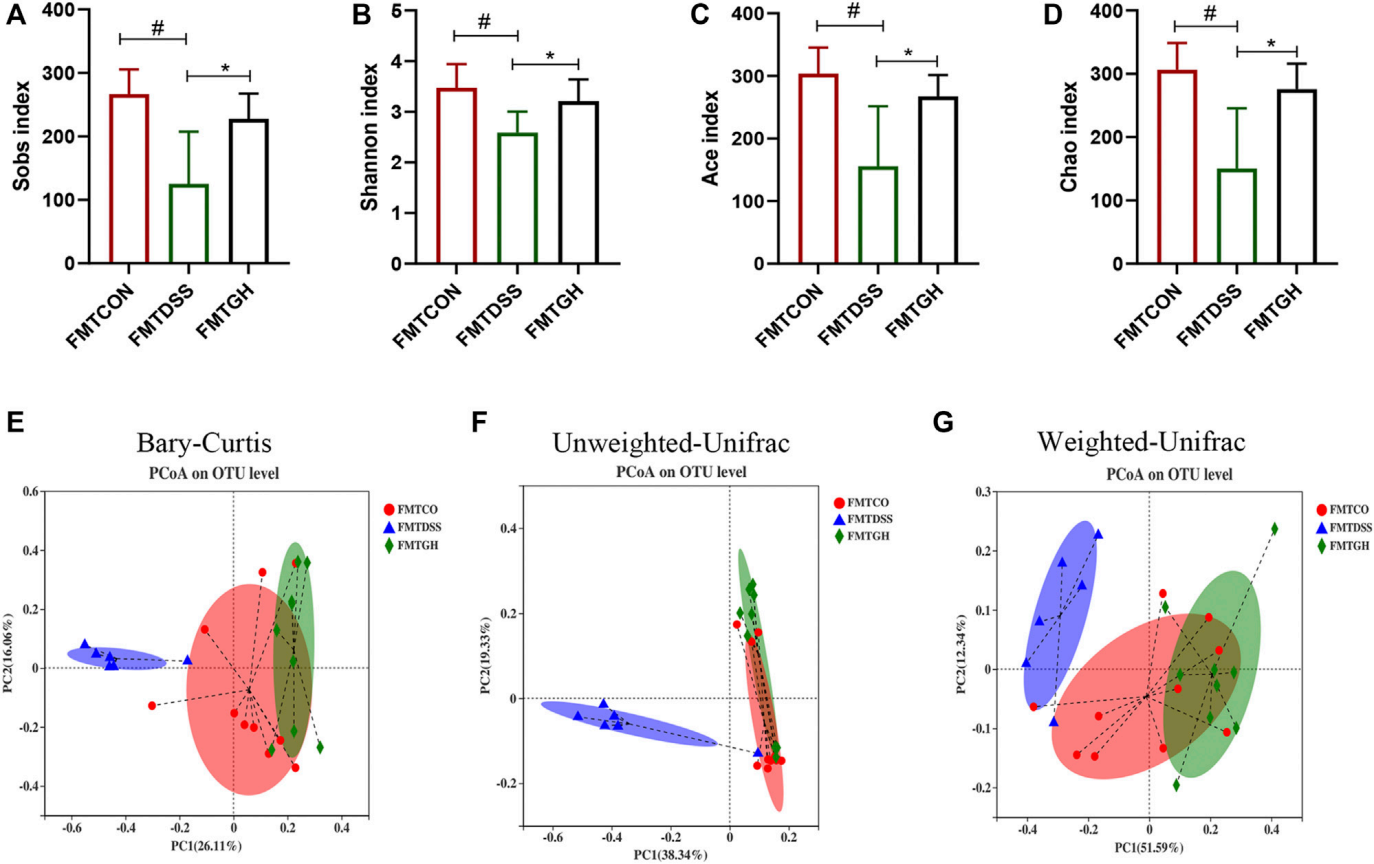
FMT changed the gut microbial diversity in colitis mice. **(A)** Sobs index. **(B)** Shannon index. **(C)** Ace index. **(D)** Chao index. **(E)** PCoA using Bray-Curtis metric distances of beta diversity. **(F)** PCoA using Unweighted-UniFrac of beta diversity. **(G)** PCoA using Weighted-UniFrac of beta diversity.

Emerging evidence has shown that FMT can prevent UC through modification of the gut microbiota (Moayyedi et al., 2015; Goyal et al., 2018; Burrello et al., 2019). To obtain stronger evidence, we similarly clarified the intestinal microbiome profile by sequencing the V3-V4 region of bacterial 16S rRNA from colon contents isolated from FMT receptor mice. Consistent with the alterations in the diversity and composition of the microbial community in the control and MGQD intervention groups, mice in the FMTCON and FMTGH groups both harbored significantly higher microbial diversity than those in the FMTDSS group, as evidenced by increased Sobs, Shannon, ACE and Chao indices on OTU abundance (Figures 6A–D). To examine alterations in intestinal microbiota composition, PCoA based on the Bray-Curtis distance, Unweighted UniFrac and Weighted UniFrac algorithm analysis showed a statistically significant separation of microbiota composition between the FMTCON and FMTDSS groups and a closer distance between the FMTGH group and the FMTCON group (Figures 6E–G).

**FIGURE 7:**
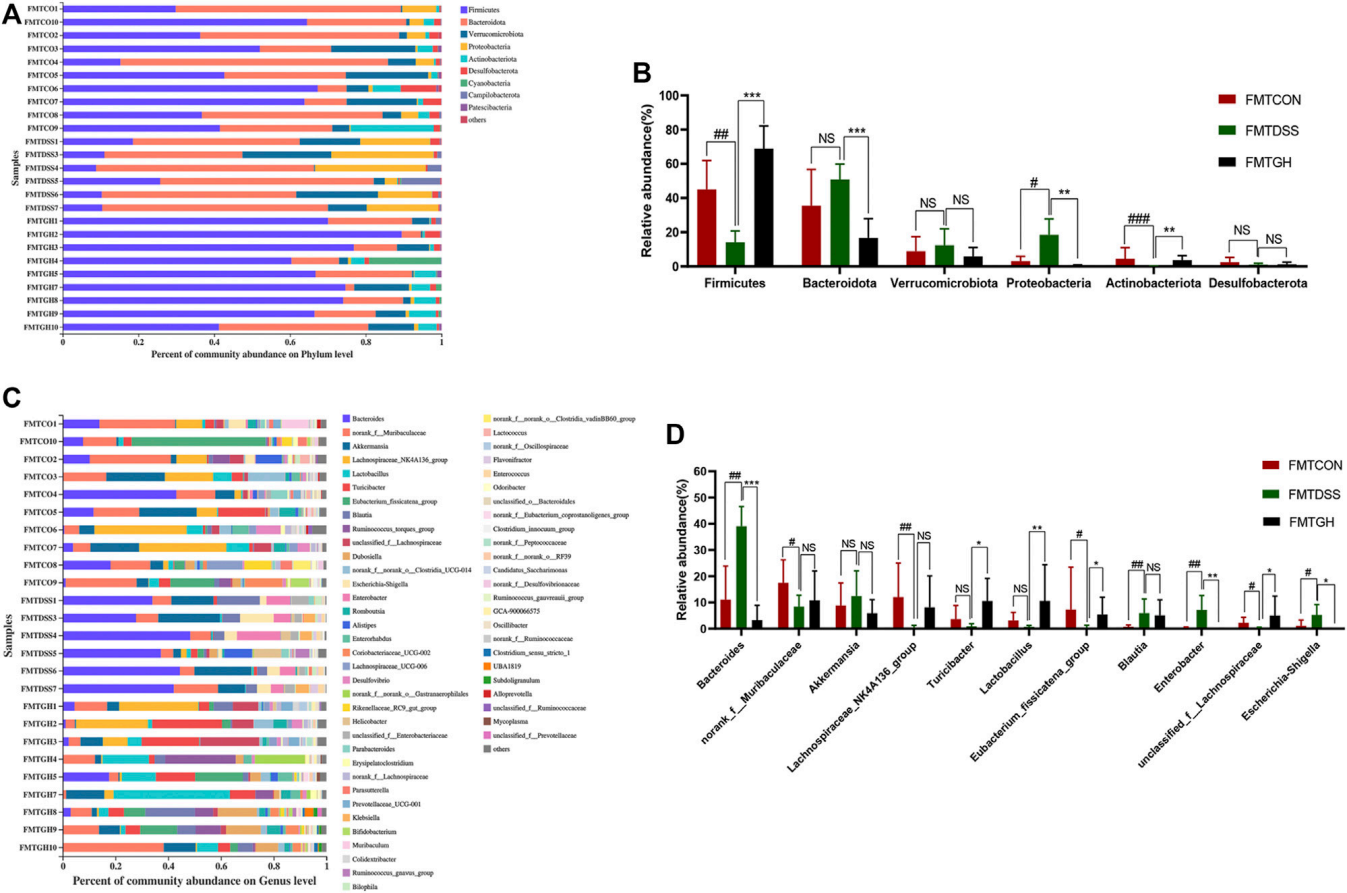
FMT altered the gut microbiota composition among different groups. **(A)** The bar plot of microbiota compositions at the phylum level among three groups and **(B)** Significant differences in the abundance of phyla with high abundance (top 6). **(C)** The bar plot of microbiota compositions at the genus level among three groups and **(D)** Significant differences in the abundance of genus with high abundance (top 11). Data were expressed as mean ± SD (n = 6–10). #*p* < 0.05, ##*p* < 0.01, ###*p* < 0.001, vs FMTCON group; *p < 0.05, **p < 0.01, ***p < 0.001 versus the FMTDSS group. NS means no statistical significance.

Taxa-based analysis revealed significant differences in intestinal microbial composition at the phylum and genus levels. At the phylum level, the dominant species of colonic microbiota in the three groups were *Firmicutes*, *Bacteroidetes*, *Verrucobacteria*, *Proteobacteria*, and *Actinobacteria*. Compared with the FMTCON group, the relative abundance of *Firmicutes* and *Actinobacteria* decreased significantly, and the relative abundance of *Proteobacteria* and *Bacteroidetes* was augmented significantly in the FMTDSS group, but the FMTGH group could significantly reverse this phenomenon (Figures 7A, B). Detailed information on the relative abundance of class, order, and family microbial communities is given in Supplementary Figure S5. At the genus level, *Bacteroides*, *Akkermansia*, *Blautia, Enterobacter* and *Escherichia-Shigella* levels were relatively higher in the FMTDSS group, while *norank_f_Muribaculaceae*, *Lachnospiraceae_NK4A136_group*, *Turicibacter, Lactobacillus*, *Eubacterium_fissicatena_group*, and *unclassified_f__Lachnospiraceae* levels were relatively lower than those observed in the FMTCON group. Pleasingly, the FMTGH group normalized the relative abundance of all the bacteria mentioned above to a large extent that were induced by DSS (Figures 7C, D).

**FIGURE 8:**
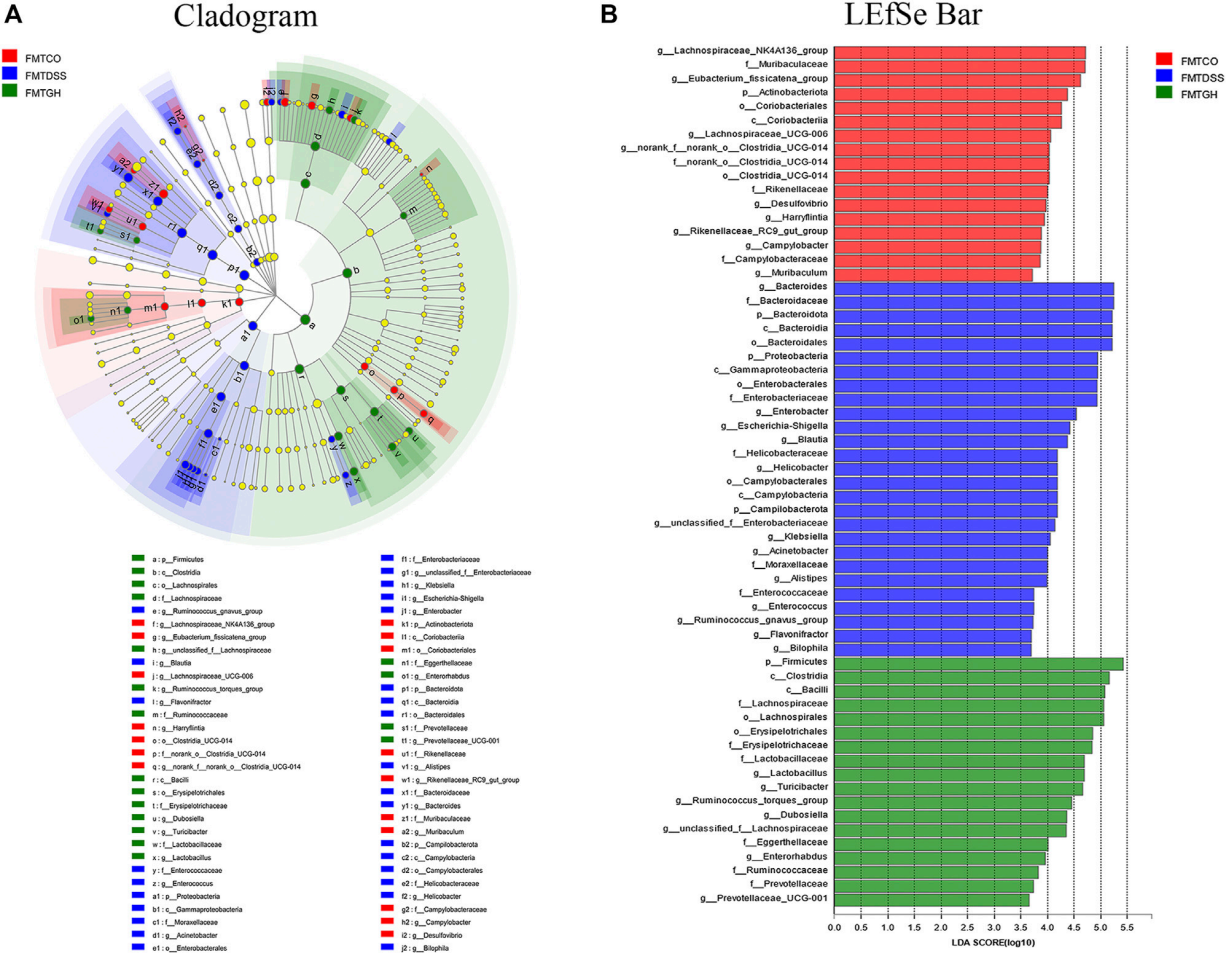
Screening of differentially abundant microbial taxa using linear discriminant analysis (LDA) coupled with effect size (LEfSe) analysis. **(A)** Cladogram obtained from LEfSe analysis with presenting various levels (from phylum to genus) from inner to outer rings. **(B)** Biomarker taxons generated from LEfSe analysis. The criteria for feature selection is log LDA score >3.6.

Subsequently, we also used LEfSe to compare high-dimensional categories and identify the predominant bacterial communities from the three groups. Our results showed that *Bacteroides* (from the phylum to the genus) was the core bacterial species that caused the imbalance of the intestinal flora in the FMTDSS group. In the FMTCON group, genus *Lachnospiraceae_NK4A136_group* and family *Muribaculaceae* were the most enriched with an LDA score of 4.72 (*p* = 0.007). *Firmicutes* was the most enriched in the FMTGH group with the highest LDA score of 5.163 (*p* = 0.003) (Figure 8). In general, transfer of MGQD-treated fecal bacteria significantly compensated for the decreased diversity and discordance of the intestinal flora caused by DSS.

### 3.7 MGQD Intervention Increased Metabolite SCFAs

**FIGURE 9:**
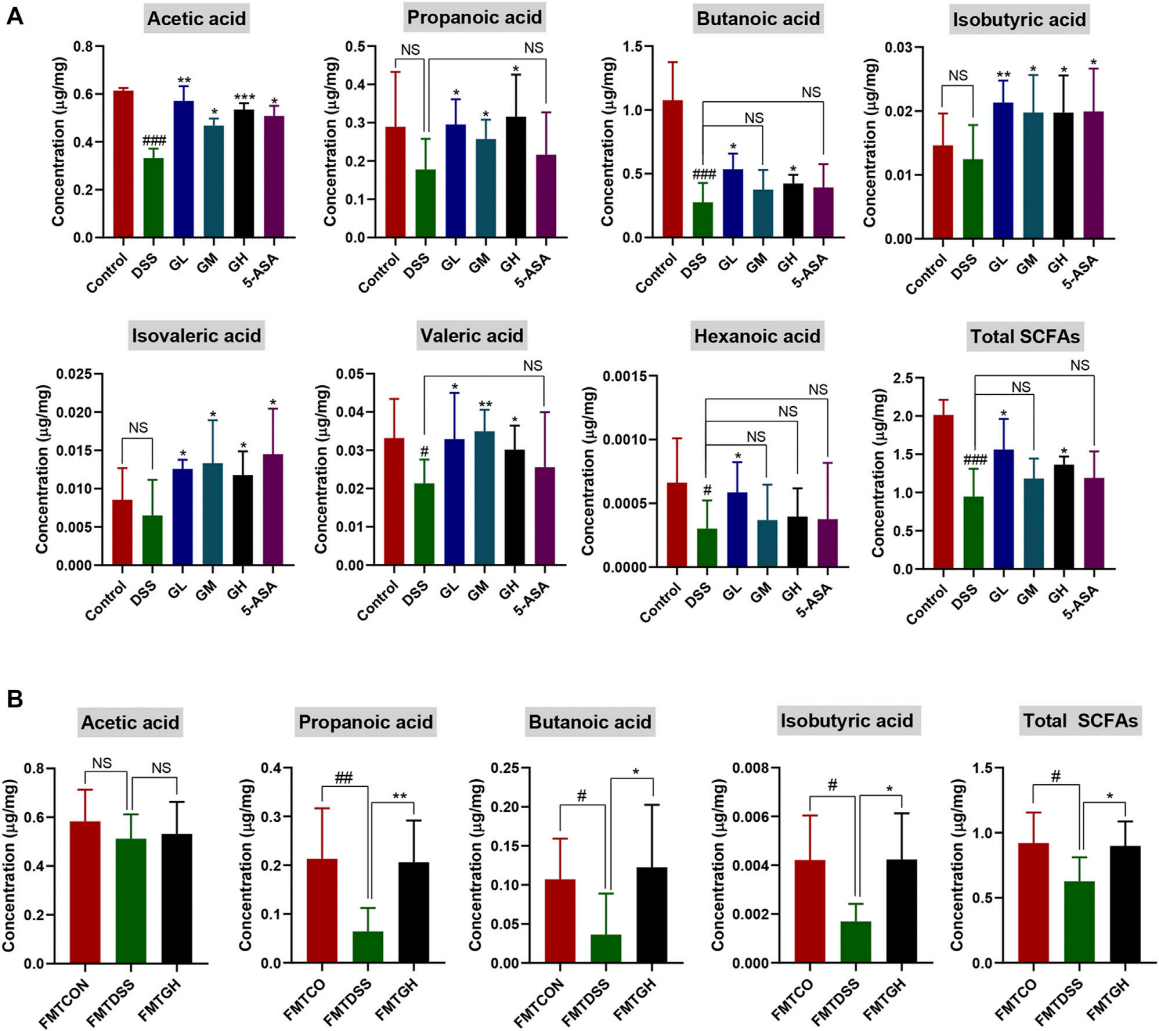
MGQD treatment and FMT enhanced the content of microbial metabolites SCFAs in DSS-treated mice. **(A)** The concentration of SCFAs, including acetic acid, propanoic acid, butanoic acid, isobutyric acid, isovaleric acid, valeric acid, hexanoic acid and Total SCFAs in cecal contents among six groups. Results were shown as mean ± SD (n = 5–9). #*p* < 0.05, ###*p* < 0.001, vs control group; *p < 0.05, **p < 0.01, ***p < 0.001, vs DSS group. **(B)** The concentration of SCFAs, including acetic acid, propanoic acid, butanoic acid, isobutyric acid and Total SCFAs in cecal contents among FMT groups. Results were shown as mean ± SD (n = 5–10). #*p* < 0.05, ##*p* < 0.01, vs FMTCON group; *p < 0.05, **p < 0.01, vs FMTDSS group. NS means no statistical significance.

In view of the key role of SCFAs in microbial-host interactions and IBD pathogenesis, we next explored whether MGQD had an impact on the production of SCFAs in colitis mice. The level of SCFAs in the cecal contents was measured by targeting metabolomics. As shown in Figure 9A, compared with the control group, the contents of acetic acid, butanoic acid, valeric acid, hexanoic acid and total SCFAs in the cecal contents of the DSS model group mice were sharply decreased. Compared with the control group, the contents of propanoic acid, isobutyric acid and isovaleric acid were decreased in the DSS group, but there was no significant difference. In comparison with DSS treatment alone, GL significantly improved the levels of the seven SCFAs mentioned above and the total SCFAs. Compared with the DSS group, the contents of these seven SCFAs and total SCFAs were increased in the GM group, but there were no statistically significant differences in butyric acid, hexanoic acid or total SCFAs. Compared with the DSS model group, the GH group had significantly increased contents of the other 6 SCFAs and total SCFAs except for hexanoic acid. 5-ASA dramatically enhanced the contents of acetic acid, isobutyric acid and isovaleric acid, and the contents of propanoic acid, butyric acid, valeric acid, hexanoic acid and total SCFAs were also observed to increase, but there was no significant difference. No significant difference was observed in isohexanoic acid among the 6 groups (data not shown).

To further clarify whether the enhancement of metabolite SCFAs was due to alterations in intestinal microbiota after MGQD administration, targeted metabolomics analysis was also carried out among the FMT groups. The cecal contents of the mice treated with FMTDSS showed decreased levels of acetic acid, propanoic acid, butyric acid, isobutyric acid, and total SCFAs compared with those of the FMTCON group, but there was no significant difference in acetic acid between the FMTCON and FMTDSS groups. Similarly, compared with the FMTDSS group, the FMTGH group exhibited augmented contents of acetic acid, propanoic acid, butanoic acid, isobutyric acid and total SCFAs, but there was no significant difference in acetic acid between the FMTGH and FMTDSS groups (Figure 9B). No significant differences were observed among the three groups in terms of hexanoic acid, isohexanoic acid, isovaleric acid and valeric acid (data not shown). In summary, these results indicated that the anti-colitis properties of MGQD may be associated with regulating the structure and composition of the intestinal bacterial community, which were related to the increased level of microbial metabolite SCFAs.

### 3.8 MGQD Intervention Suppressed Proinflammatory Cytokines and Promoted Anti-Inflammatory Cytokines in DSS-Induced Colitis

**FIGURE 10:**
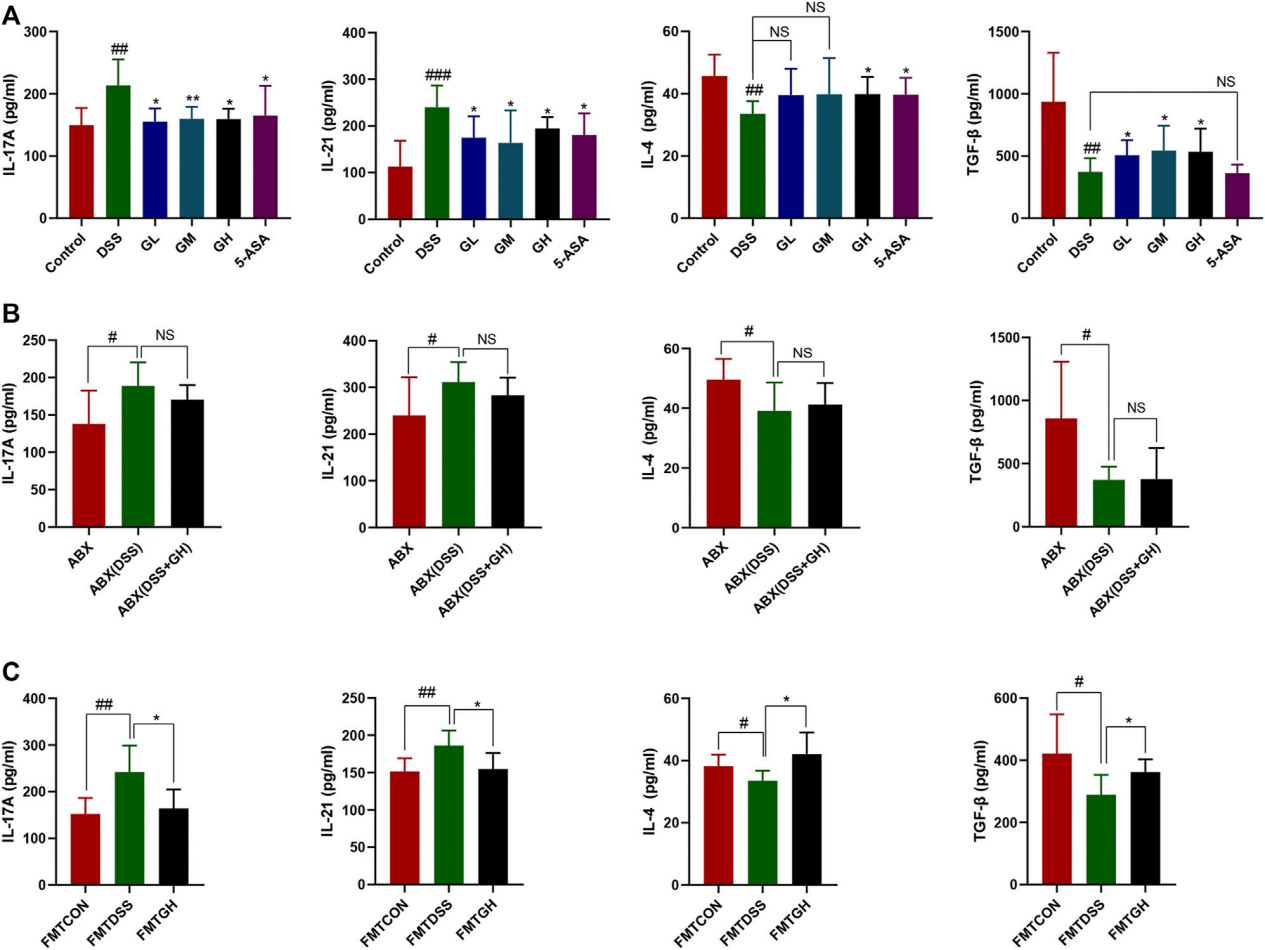
MGQD and FMT attenuated inflammatory response in colonic tissues of mice with DSS-induced colitis. **(A)** The expression levels of pro-inflammation cytokines IL-17A, IL-21 and anti-inflammation cytokines IL-4 and TGF-*β* were determined in colon tissue homogenate using ELISA methods among six groups. Results were shown as mean ± SD (n = 5–10). ##*p* < 0.01, ###*p* < 0.001, vs control group; *p < 0.05, **p < 0.01, vs DSS group. **(B)** IL-17A, IL-21, IL-4 and TGF-*β* cytokines levels in colon tissue homogenate were measured by ELISA between gut microbiota depletion groups. Results were shown as mean ± SD (n = 6–10). #*p* < 0.05, vs ABX group. **(C)** IL-17A, IL-21, IL-4 and TGF-*β* cytokines levels in colon tissue homogenate were measured by ELISA between FMT groups. Results were shown as mean ± SD (n = 5–10). #*p* < 0.05, ##*p* < 0.01, vs FMTCON group; *p < 0.05, vs FMTDSS group. NS means no statistical significance.

The expression levels of the inflammatory cytokines IL-4, TGF-*β*, IL-17A and IL-21 in the colon tissues of each group were quantitatively analyzed by the corresponding ELISA kits to investigate the inflammatory response to MGQD treatment. As shown in Figure 10A, compared with the normal group, the expression of the anti-inflammatory cytokines IL-4 and TGF-*β* was dramatically reduced in the DSS model group, while the expression of the proinflammatory cytokines IL-17A and IL-21 was significantly increased. Compared with the DSS model group, the content of TGF-*β* was significantly increased in all dosages of MGQD groups, and the level of IL-4 was significantly increased only in the GH and 5-ASA groups. The content of IL-17A in all dosages of MGQD groups and in the 5-ASA group was significantly decreased, and the concentrations of IL-21 in the GL, GH, and 5-ASA groups were significantly decreased in comparison with that of the DSS group. The abovementioned cytokines in colon tissue were also detected in the intestinal microbial depletion groups. Notably, IL-17A, IL-21, IL-4 and TGF-*β* showed similar levels, with no significant difference between the ABX (DSS) and ABX (DSS + GH) groups (*p* > 0.05) (Figure 10B).

Targeted metabonomics showed that FMTGH increased the metabolite SCFAs; thus, we hypothesized that FMT could also have an impact on the expression of cytokines. As expected, colonic tissues in the FMTCON and FMTGH groups both had lower levels of IL-21 and IL-17A but higher levels of TGF-*β* and IL-4 than colonic tissues in the FMTDSS group with a significant difference (Figure 10C). In conclusion, MGQD significantly alleviated colitis by downregulating the levels of proinflammatory cytokines and upregulating anti-inflammatory cytokines.

### 3.9 MGQD Intervention Shaped Intestinal Immune Responses by Restoring Treg/Th17 Balance

**FIGURE 11:**
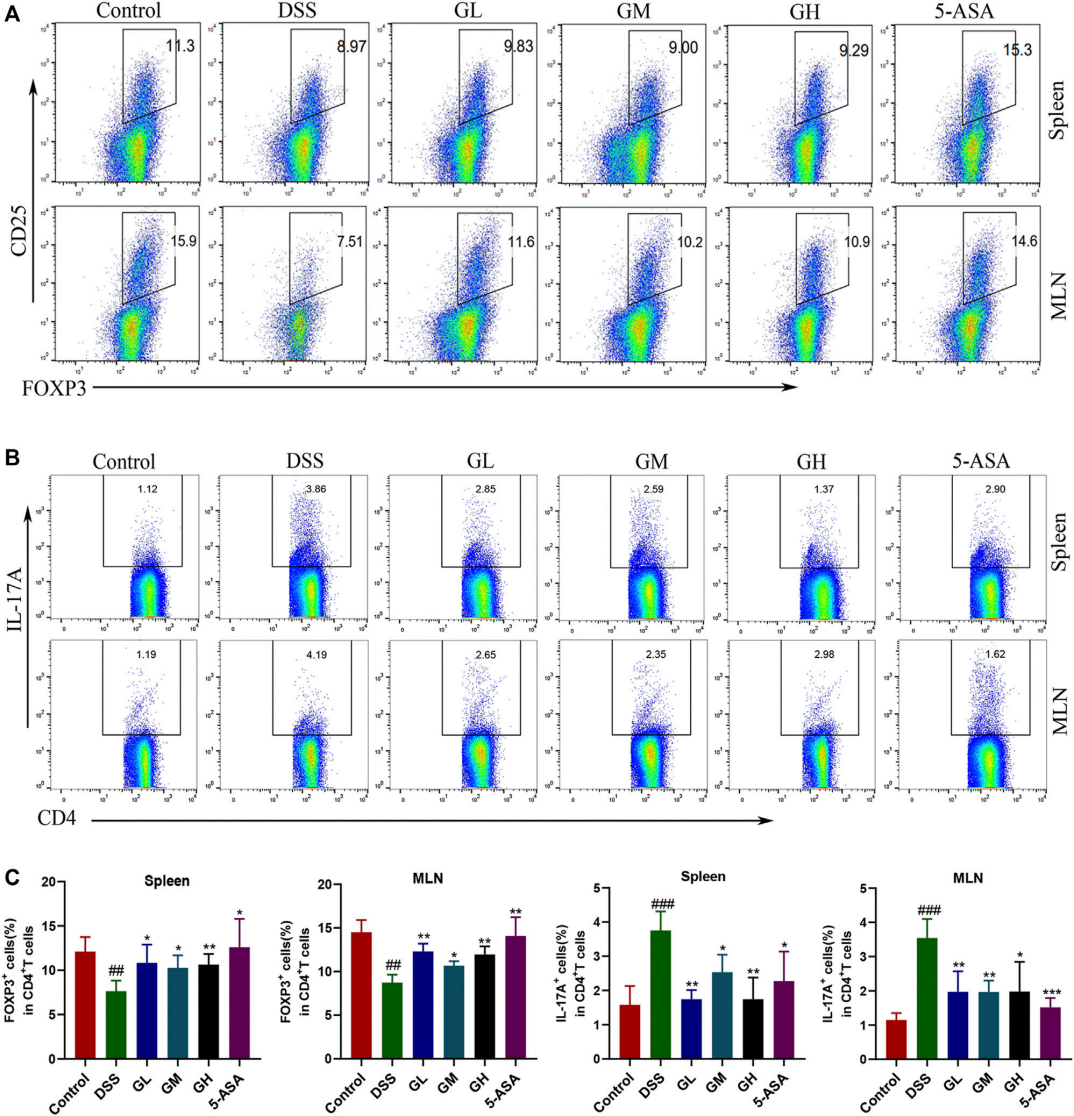
MGQD traetment regulated the proportion of Treg cells and Th17 cells in splenocytes and mesenteric lymph nodes (MLNs) in UC mice. **(A)** Effect of MGQD on the proportion of CD4^+^CD25 + Foxp3+ (Treg) cells in the spleen and MLNs of mice with colitis were analyzed by flow cytometry. **(B)** Effect of MGQD on the proportion of CD4+IL-17A+ (Th17) cells in the spleen and MLNs of mice with colitis. **(C)** Quantitative analysis of Treg and Th17 cells in splenocytes and MLNs. Data were expressed as mean ± SD (n = 3–5). ##*p* < 0.01, ###*p* < 0.001, vs control group; *p < 0.05, **p < 0.01, vs DSS group.

Considering that the Treg/Th17 imbalance plays an important role in the maintenance of intestinal microecosystem homeostasis and intestinal immune function, flow cytometry was used to detect T cell response phenotypes in spleens and MLNs to explore the effect of MGQD intervention on the Treg/Th17 balance. Compared with the normal group, the ratio of CD4+IL-17A+/CD4+ lymphocytes in the spleens and MLNs in the DSS model group was significantly increased, while the ratio of CD4^+^CD25 + Foxp3+/CD4+ lymphocytes was significantly decreased, but all dosages of MGQD treatment groups and the 5-ASA group significantly reversed this phenomenon (Figure 11). In conclusion, MGQD administration selectively promoted the frequency of Treg cells and suppressed the proportion of Th17 cells against DSS-induced colitis to maintain intestinal homeostasis.

### 3.10 MGQD Intervention Restored Treg/Th17 Balance in a Gut Microbiota-Dependent Manner

**FIGURE 12:**
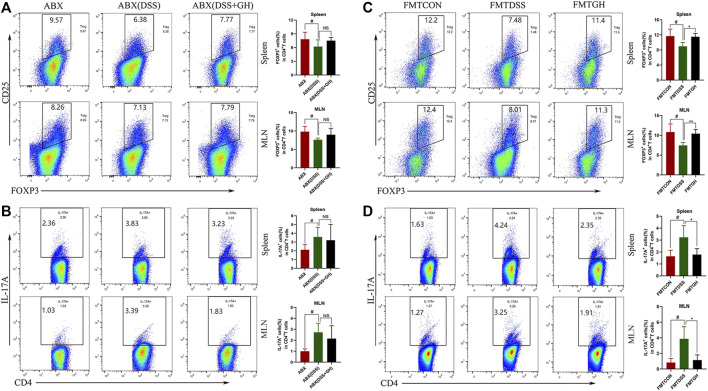
The frequency of Treg cells and Th17 cells in mice splenocytes and MLNs after gut microbiota depletion and FMT. **(A)** Treg cells in the spleen and MLNs from ABX treatment groups were detected by flow cytometry and bar charts of the percentage of Treg cells were presented. **(B)** Representative plot and quantitative analysis of Th17 cells in the splenocytes and MLNs of mice from ABX treatment groups. Data were expressed as mean ± SD (n = 3-5 for MLNs samples and n = 5–10 for spleen samples). #*p* < 0.05, vs ABX group. **(C)** Representative plot and graph analysis of Treg cells in mice splenocytes and MLNs from FMT groups were displayed. **(D)** The ratio of Th17 cells produced in murine splenocytes and MLNs were determined by flow cytometry and bar charts of the percentage of Th17 cells were presented. Data were expressed as mean ± SD (n = 3–5). #*p* < 0.05, vs FMTCON group; *p < 0.05, vs FMTDSS group. NS means no statistical significance.

To further confirm whether the restoration of the Treg/Th17 balance after MGQD administration relied on the intestinal microbiota, the phenotypes of single-cell suspends isolated from the spleens and the MLNs of mice in the ABX model experiment were also analyzed by flow cytometry. Interestingly, neither the percentage of Treg cells nor the percentage of Th17 cells in the spleens and MLNs showed significant differences between the ABX (DSS + GH) and ABX (DSS) groups (Figures 12A,B). Phenotypic analysis of the frequency of Treg and Th17 cells in the spleens and MLNs was also performed in the FMT experiment. Compared with the FMTCON group, the spleens and MLNs of the FMTDSS group showed a decreased frequency of Treg cells but a higher percentage of Th17 cells. In sharp contrast, the FMTGH group had a higher percentage of Treg cells but a lower proportion of Th17 cells than the FMTDSS group. The results showed that transferring MGQD-treated fecal microbiota reversed the imbalance of Th17 and Treg cells in UC mice (Figures 12C,D). These results demonstrated that the intestinal microflora modified by MGQD administration was associated with the modulation of the Treg/Th17 balance. In conclusion, MGQD regulated the Treg/Th17 balance in a gut microbiota-dependent manner.

## 4 Discussion

In the present study, we adopted a mouse model of colitis induced by DSS, a widely used classical chemical inducer (Wirtz et al., 2017), and our results demonstrated that MGQD exerted a significant protective effect on the colitis mice, as evidenced by strikingly decreased weight loss, DAI score, shortened colon length, histological score, decreased proinflammatory cytokines and increased anti-inflammatory cytokines. The gut microbiota showed richer microbial diversity and an altered flora profile after intervention with MGQD, which promoted the production of SCFAs, thereby improving the Treg/Th17 balance in the spleens and MLNs. Most importantly, intestinal bacterial depletion and FMT experiments further provided stronger evidence that these mechanisms can be attributed to its modification of the gut microbiota. Overall, we demonstrate for the first time that MGQD has a profound impact on modifying the structure of the intestinal microbiota, particularly the abundance of bacteria-generating SCFAs, leading to the recovery of SCFAs production, the reversal of the imbalance of Treg/Th17 cells and ultimately the reconstruction of tissue homeostasis.

To evaluate the effect of MGQD on UC, phenotypic examination of mice with induced colitis confirmed that colitis resulted in loss of body weight and colon length, increased DAI scores, a disordered intestinal gland structure, infiltration of inflammatory cells, destruction of intestinal crypts, and reduction of goblet cells, but these effects were markedly reversed by all doses of MQGD and 5-ASA treatment, indicating that MGQD intervention significantly hindered the progression of colitis in the mice, thereby abating colonic inflammation and damage. Additionally, the disorders mentioned above in the intestinal microenvironment have previously been associated with pathogenic taxa, such as *Escherichia-Shigella* and *Proteobacteria*, enriched in inflammatory conditions and contribute to disease progression (Ni et al., 2017). Previous evidence has also suggested that disruption of the equilibrium of the intestinal microbiota plays a vital role in the etiopathogenesis of IBD (Honda and Littman, 2016; Belkaid and Harrison, 2017; Britton et al., 2019; Lee and Chang, 2021). Considering that the multiple effects of intestinal flora and GQD have a good anti-colitis effect, studies on the potential mechanism between exogenous fecal microbiota intervention after MGQD treatment and the regulation of Treg/Th17 cells have rarely been reported from the perspective of intestinal flora receiving MGQD for IBD treatment. Hence, we hypothesized that the gut microbiota may participate in the protective effect of MGQD on colitis. To this end, we then performed the ABX method to deplete the gut microbiota. However, no significant differences were detected between the ABX (DSS) and ABX (DSS + GH) groups in terms of body weight percentage, DAI score, colon length or tissue damage, or expression of inflammatory cytokines (e.g., IL-17A, IL-21, TGF-*β* and IL-4). Therefore, mice with depleted microbiota were also susceptible to DSS-induced colitis, and the alleviating effect of MGQD on the mice was abolished. This suggests that MGQD treatment could not rescue the depletion of beneficial bacterial communities in colitis. We then conducted FMT to further confirm these phenomena. Encouragingly, compared with the FMTDSS group, mice with reconstituted microbiota in the FMTGH group manifested alleviated symptoms of colitis and intestinal damage. After FMT, the protective effect of MGQD on colon inflammation reappeared. The FMTDSS group and FMTGH group showed significant differences in the severity of colitis. Therefore, alterations in the gut microbiota regulated by MGQD administration were responsible for relieving colitis.

The gut microbiota plays a core role in maintaining immune homeostasis and affects chronic inflammation in humans and animals (Ni et al., 2017). Christina et al. demonstrated a correlation between the presence or absence of intestinal microbiota and the severity of inflammation in germ-free mice (Hernández-Chirlaque et al., 2016). In addition, several studies have shown that decreased microbial richness, increased ecological imbalance index, and higher individualized microbial instability are closely associated with the risk of IBD (Lepage et al., 2011; Britton et al., 2019). Herein, 16S gene rRNA sequencing was utilized to identify alterations in gut microbial diversity and composition after MGQD intervention. A decrease in microbial diversity is common in IBD, and the microbial community composition of the DSS-induced UC model group was apparently different from that of the normal group. Concordant with these findings, our data also demonstrated that a significantly decreased Shannon index was obviously observed in the DSS model mice but was effectively reversed by MGQD supplementation and 5-ASA. In terms of beta diversity, PCoA showed obvious cluster separation between the mice in the MGQD groups and the mice in the DSS group but a smaller distance between the MGQD groups and the normal group, indicating that MGQD treatment markedly normalized the biological community structure. The decrease in intestinal microbial diversity associated with ecological imbalance may be due to changes in the balance between symbiotic microorganisms and potentially pathogenic microorganisms (Ott et al., 2004; Sepehri et al., 2007; Hirano et al., 2018). Our study showed that some taxonomic microbiota exhibited a higher relative abundance in the DSS model group, including *Bacteroides*, *Escherichia-Shigella*, *Alistipes, Turicibacter* and *Clostridium_sensu_stricto_1,* while displaying a lower relative abundance of SCFA-producing bacteria, such as *Lactobacillus* and *Lachnospiraceae_NK4A136_group* than that in the control group, but some of them tended to be normalized to a certain extent somewhat following MGQD treatment. Next, LEfSe analysis revealed that the family *Ruminococcaceae*, the *g_norank_f__Lachnospiraceae*, and the family *Lachnospiraceae*, the bacteria boosted to generate SCFAs, were the predominant bacteria in the GL, GM and GH groups, respectively. In addition, the intestinal flora profiles of the DSS receptor (FMTDSS) and MGQD receptor (FMTGH) mice were similar to those of the DSS model group and MGQD-treated mice, respectively. This confirmed the role of MGQD in the regulation of intestinal microbiota.

SCFAs are the major metabolites of the intestinal microbiome and are beneficial for colon health and integrity. Emerging evidence has suggested that reduced concentrations of SCFAs have been observed in IBD patients and DSS-induced colitis mouse models (Arpaia et al., 2013; Smith et al., 2013; Zeng and Chi, 2015; Gonçalves et al., 2018; Sun et al., 2020). Taking the predominant bacteria in the MGQD groups associated with the metabolism of SCFAs into consideration, we evaluated the concentration of SCFAs in the cecal samples. Our results showed that MGQD administration markedly facilitated the production of microbial-derived SCFAs. In addition, the same trend was evident in the FMT experiment.

Accumulating studies have shown that metabolic factors from external and internal factors shape the abundance and activity of Treg cells. In addition, metabolites derived from symbiotic microbiota, such as SCFAs, have been shown to affect Treg homeostasis and function in GALT (Geuking et al., 2011; Zeng and Chi, 2015). Furthermore, dietary supplementation with SCFAs alone increased Treg percentages in antibiotic-exposed mice and could even relieved colitis symptoms (Furusawa et al., 2013). It is worth noting that ATP derived from symbiotic microorganisms promotes intestinal Th17 production, suggesting that microbiome metabolism controls the differentiation of the Th17 lineage in GALT (Atarashi et al., 2008). The finding of specific T cell subpopulations in microbial-dependent induction indicates that different T cell subpopulations may distinguish different symbiotic microbial antigens (Zeng and Chi, 2015). Treg/Th17 axis imbalance is an important feature of IBD, and IBD is aggravated in the case of dysfunction of the Treg compartment, highlighting the functional relationship between Tregs and microbial homeostasis (Liu et al., 2020; Fan et al., 2021). Our study showed that MGQD treatment significantly suppressed the generation of Th17 cells and simultaneously promoted the differentiation of Treg cells inhibited by DSS, as evidenced by an increase in the frequency of CD4^+^CD25 + Foxp3+ T cells and a decrease in the percentage of CD4+IL-17A + cells compared with the DSS group, further confirming the causal relationship between colon microbiota, metabolism and inflammation. Most importantly, our data also showed that the unbalanced Treg/Th17 axis could be significantly restored by transferring the fecal microbiota of MGQD-treated mice, which may be related to the regulatory effect of MGQD on the gut microbiota, as we observed that this effect could be entirely counteracted when we depleted the gut microbiota using antibiotic cocktails. These data indicated that MGQD reversed the Treg/Th17 balance in a manner dependent on the gut microbiota. Additionally, the level of metabolite SCFAs in mice transplanted with GH-treated fecal microbiota increased sharply. Collectively, our results showed that intestinal microbiota (especially SCFA-generating microbiota) and related metabolite SCFAs were responsible for the profound ameliorative effect of MGQD.

The multiple therapeutic mechanisms of MGQD may be closely associated with its active components as identified by HPLC. Some of these monomers have been reported to have remarkable ameliorative effects in UC models. Puerarin has been shown to reconstruct the mucus layer, regulate mucin-utilizing bacteria, and inhibit inflammation and oxidative stress to alleviate UC in animal models (Wu et al., 2020a; Jeon et al., 2020). Baicalin has been shown to alleviate UC by regulating Treg/Th17 balance, intestinal flora, short-chain fatty acids and the IKK/IKB/NF-KB signaling pathway (Shen et al., 2019; Zhu et al., 2020). Palmatine can improve the colitis induced by DSS in mice by inhibiting tryptophan metabolism and regulating intestinal microbiota (Zhang et al., 2018). Berberine can alleviate UC by regulating intestinal microflora and modulating the Treg/Th17 balance (Cui et al., 2018). Wogonoside protects mice from experimental colitis by inhibiting the activation of NF-*κ*B and the NLRP3 inflammasome (Sun et al., 2015).

In view of the fact that the results of the study can be used as a guideline for the treatment of diseases, it is important to fully discuss the risks of using excessively high doses in the field of ethnopharmacology (Heinrich et al., 2020). As previously reported, the mouse dose in our study was converted according to the body surface area and the corresponding clinically prescribed dose for a 60 kg human body (66 g (raw herbs)/60 kg/d) (Song et al., 2021). Therefore, 10 g crude herbs/kg is almost equivalent to the human therapeutic dose, 5 g crude herbs/kg and 20 g crude herbs/kg are almost 0.5-fold and 2-fold of the human therapeutic dose, respectively. The doses of MGQD extract used in mice in this study were also consistent with that in most previous studies, that is, the dosage of decoction in rodents reached crude drug g/kg/d (Luo et al., 2019; Ma et al., 2021; Song et al., 2021). However, the standardization of bioactive compounds in MGQD and future studies at lower doses levels to evaluate the anti-UC potential of MGQD decoction are worthy of further exploration.

Taken together, our study demonstrates for the first time that the TCM prescription MGQD is a promising modulator of the intestinal microbiota, through which MGQD can remodel the intestinal microbiota, maintain the abundance of SCFA-producing bacteria, reestablish immune and tissue homeostasis, and thus prevent colitis in mice. These results highlight the role of MGQD in regulating intestinal microbiota composition and alleviating the intestinal inflammatory response, and targeting specific microbial species may have unique therapeutic prospects for ulcerative colitis. However, the underlying mechanisms by which MGQD antagonizes the DSS effect to normalize the intestinal microbiome composition and facilitate the survival or expansion of SCFA-producing bacteria and whether MGQD also exerts anti-inflammatory effects *in vitro* in a gut microbiota-dependent manner need to be further investigated.

## 5 Conclusion

**FIGURE 13:**
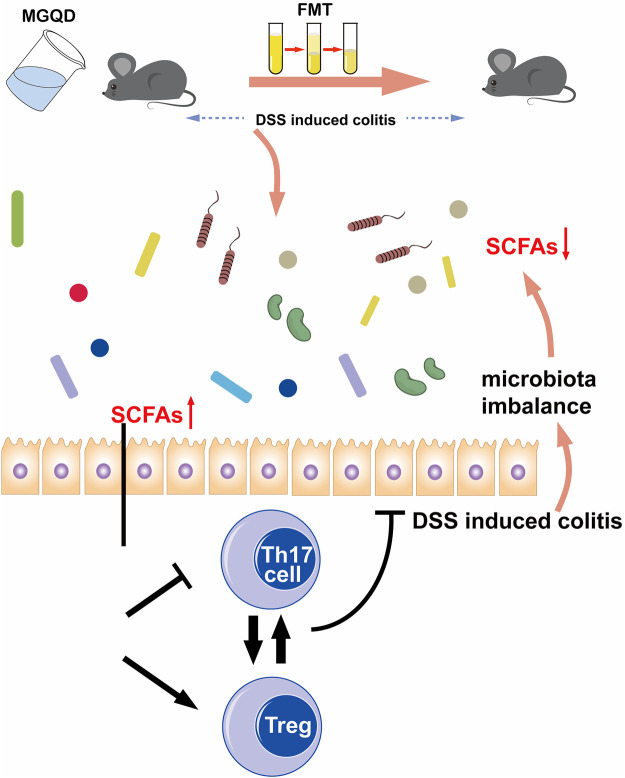
Schematic illustration of the protective mechanisms of MGQD against DSS-induced experimental colitis via restoring Treg/Th17 balance modulated by enhanced microbiota-generated SCFAs production.

In conclusion, our data suggest that the improvement of colon inflammation by MGQD administration is dependent on intestinal flora. The potential protective mechanism is related to the restored Treg/Th17 balance modulated by enhanced microbiota-generated SCFA production (Figure 13).

## Data Availability

The original contributions presented in the study are publicly available. This data can be found here: https://www.ncbi.nlm.nih.gov/bioproject/, PRJNA760298.

## Glossary

ATP: adenosine triphosphate
DAI: disease activity index
DSS: dextran sodium sulfate
ELISA: enzyme-linked immunosorbent assay
FMT: fecal microbiota transplantation
FMTCON: fecal microbiota transplantation from control mice
FMTDSS: fecal microbiota transplantation from DSS mice
FMTGH: fecal microbiota transplantation from GH treated mice
FOXP3: forkhead box protein P3
GALT: gut associated lymphoid tissue
GQD: Gegen Qinlian decoction
HE: haematoxylin and eosin
HPLC: High-Performance Liquid Chromatography
IBD: inflammatory bowel disease
IKK: IkappaB kinase
IL-6: interleukin-6
IL-10: interleukin-10
IL-17A: interleukin-17A
IL-21: interleukin-21
Jak2: janus activating kinase 2
LDA: linear discriminant analysis
LEfSe: linear discriminant analysis of effect size
MGQD: modified Gegen Qinlian decoction
MLNs: mesenteric lymph nodes
NF-κB: nuclear factor kappa-B
NLRP3: NOD-like receptor protein 3
OTUs: operational taxonomic units
PcoA: principal coordinates analysis
SCFA: short-chain fatty acids
Stat3: signal transducer and activator of transcription 3
TCM: traditional Chinese medicine
TGF-*β*: transforming growth factor-*β*
Th17: T helper 17
Treg: regulatory T
UC: ulcerative colitis
