# Supplementary material for: Modified Gegen Qinlian Decoction Regulates Treg/Th17 Balance to Ameliorate DSS-Induced Acute Experimental Colitis in Mice by Altering the Gut Microbiota
Source: Front Pharmacol. 2021 Nov 4;12:756978. doi: 10.3389/fphar.2021.756978 (PMC8601377; doi:10.3389/fphar.2021.756978)

Treg-Spleen

Control

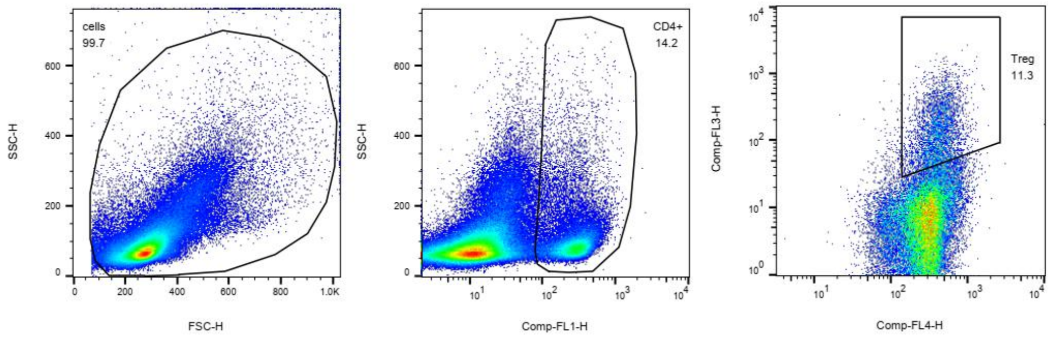

DSS

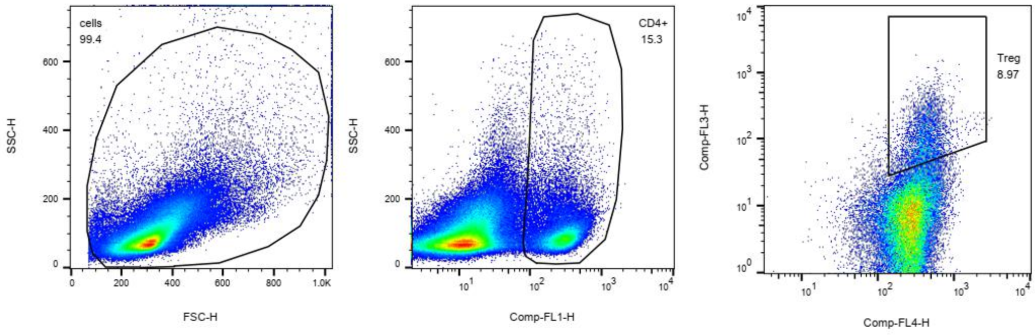

GL

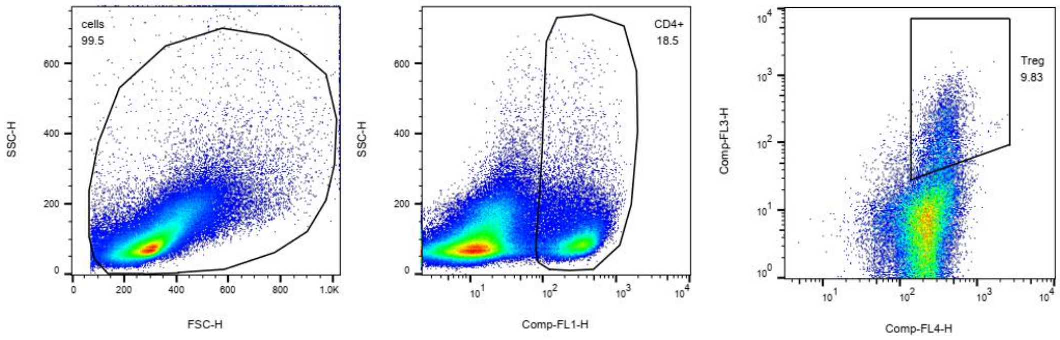

GM

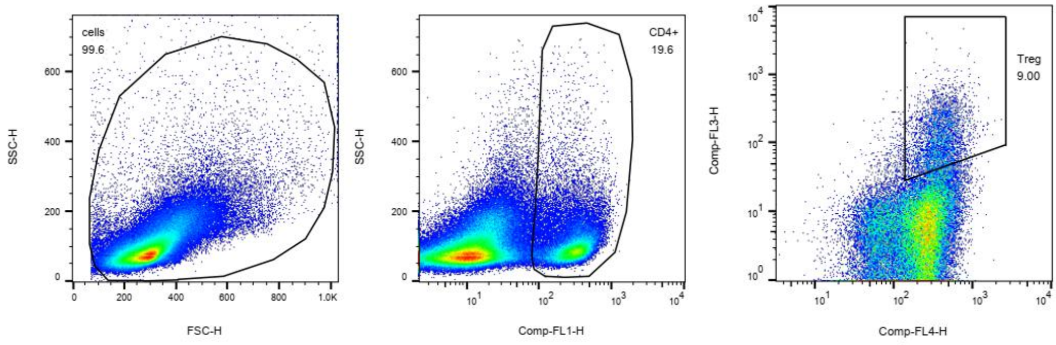

GH

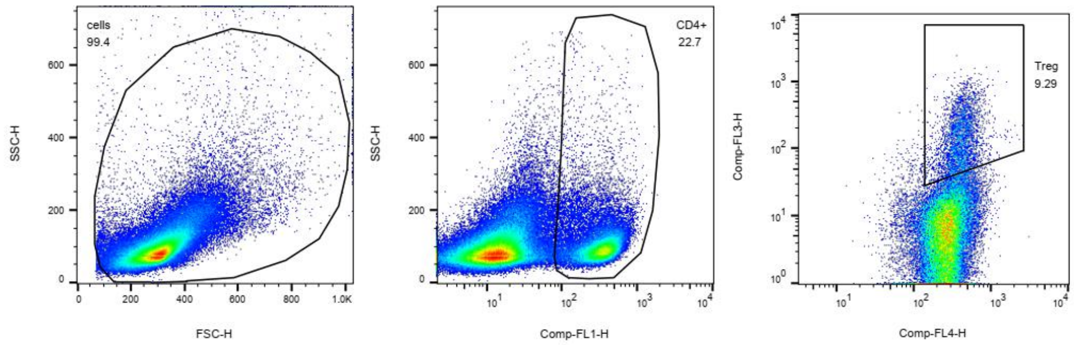

5-ASA

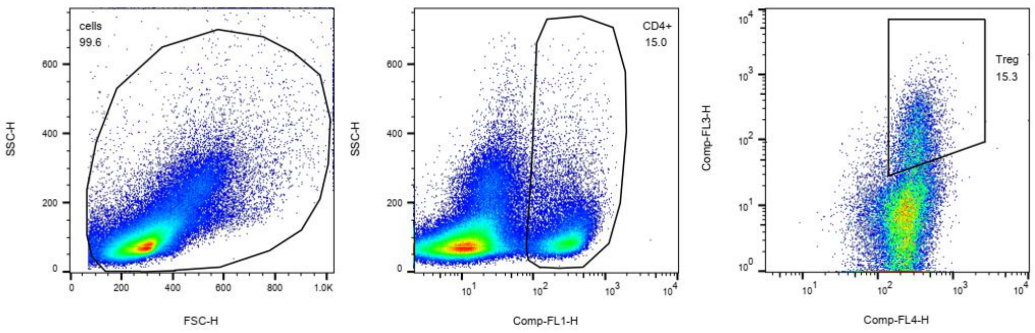

Treg-MLN

Control

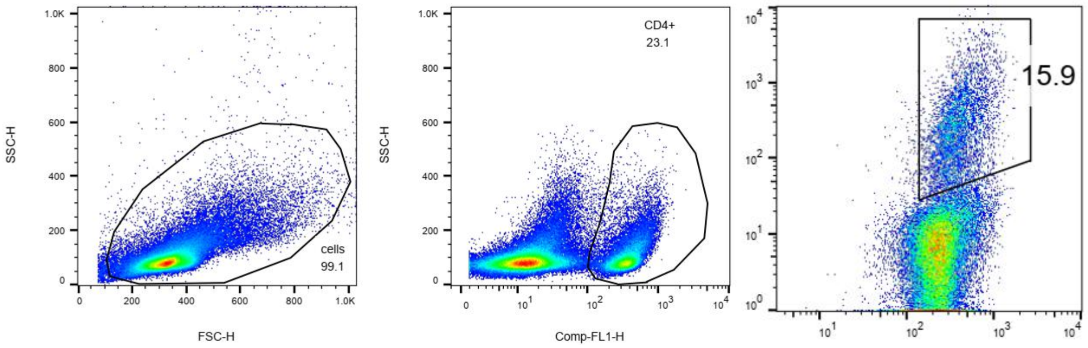

DSS

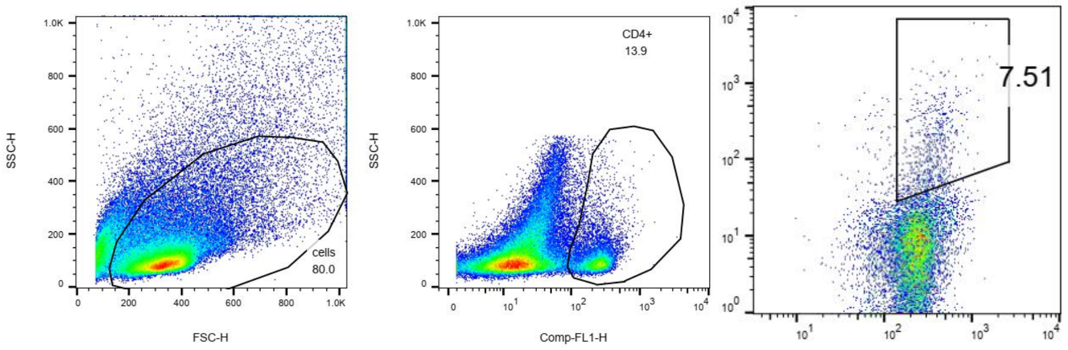

GL

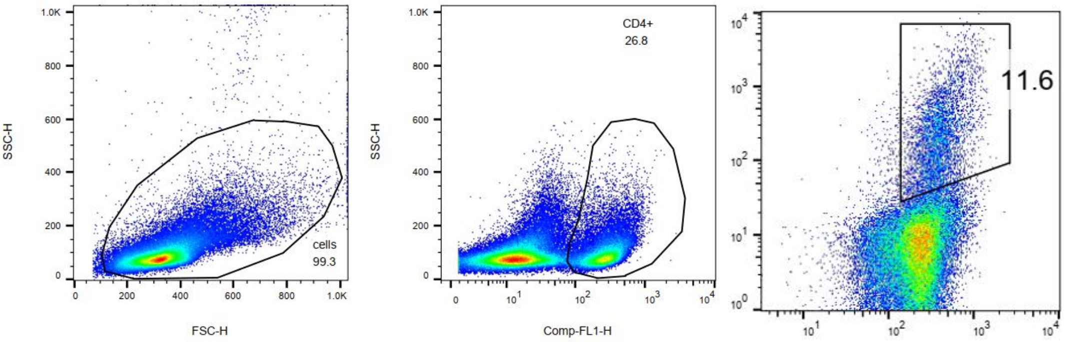
SSC-H
FSC-H
cells
99.3
CD4+
26.8
Comp-FL1-H
11.6

GM

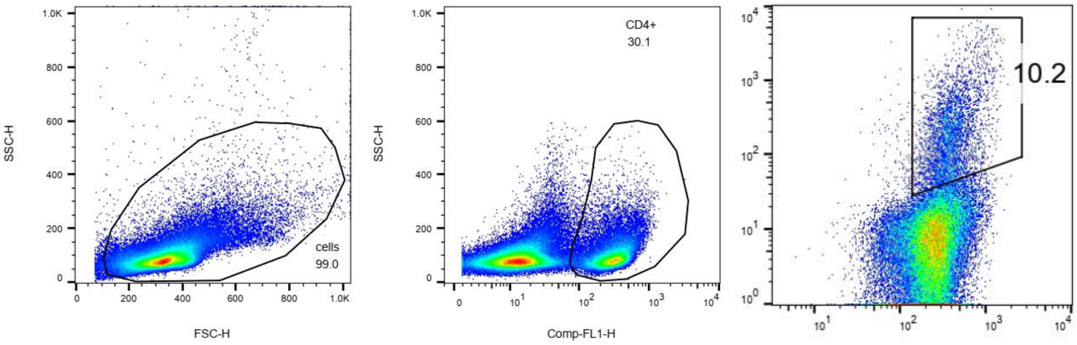
SSC-H
FSC-H
cells
99.0
CD4+
30.1
Comp-FL1-H
10.2

GH

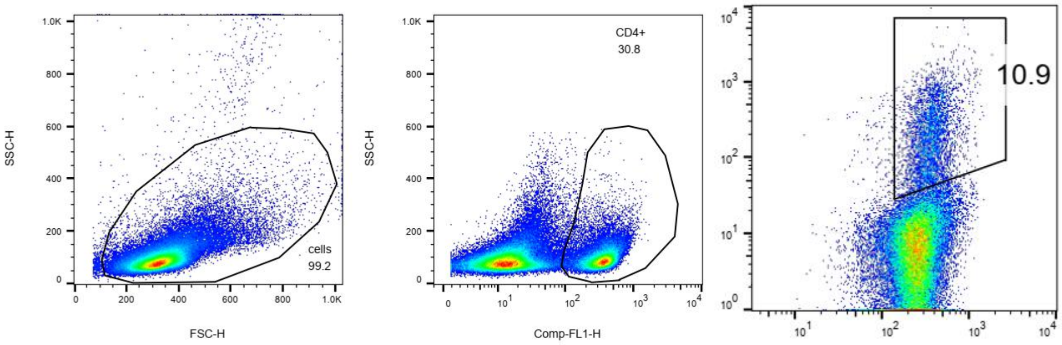
SSC-H
FSC-H
cells
99.2
CD4+
30.8
Comp-FL1-H
10.9

5-ASA

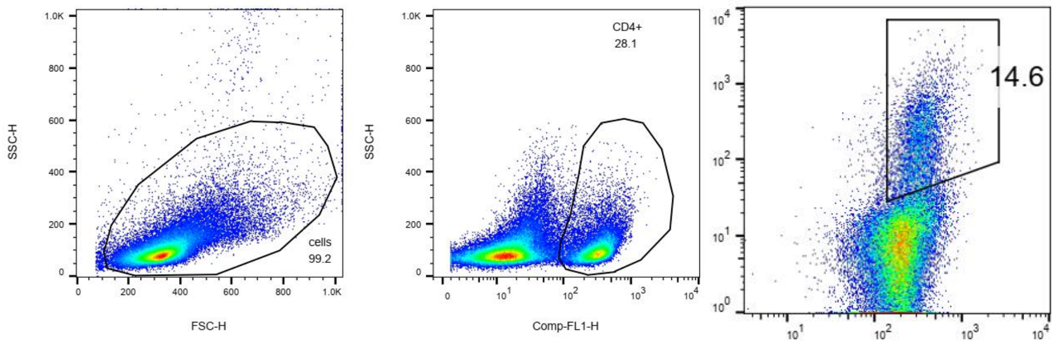
SSC-H
FSC-H
cells
99.2
CD4+
28.1
Comp-FL1-H
14.6

Th17-Spleen

Control

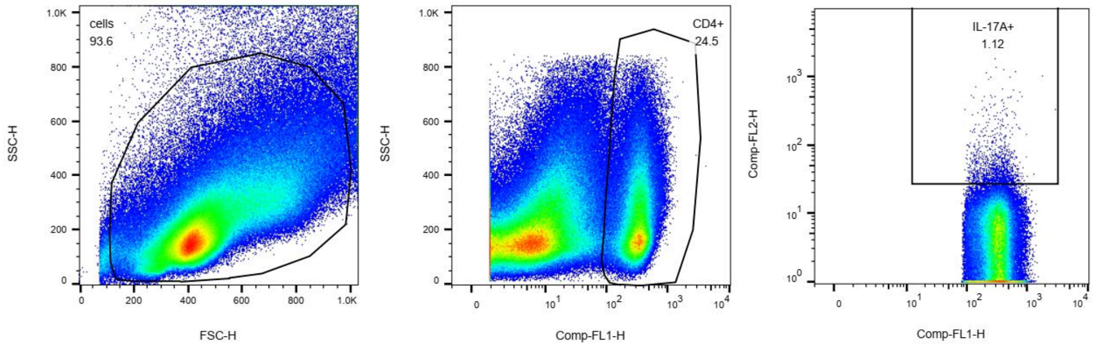

DSS

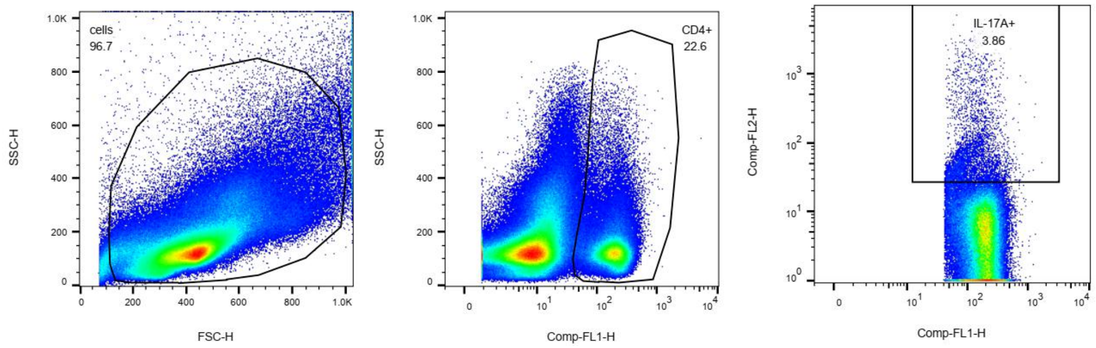

GL

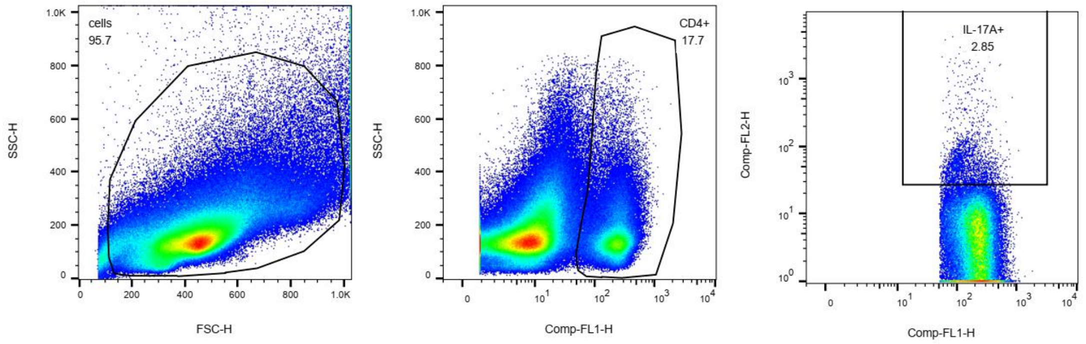

GM

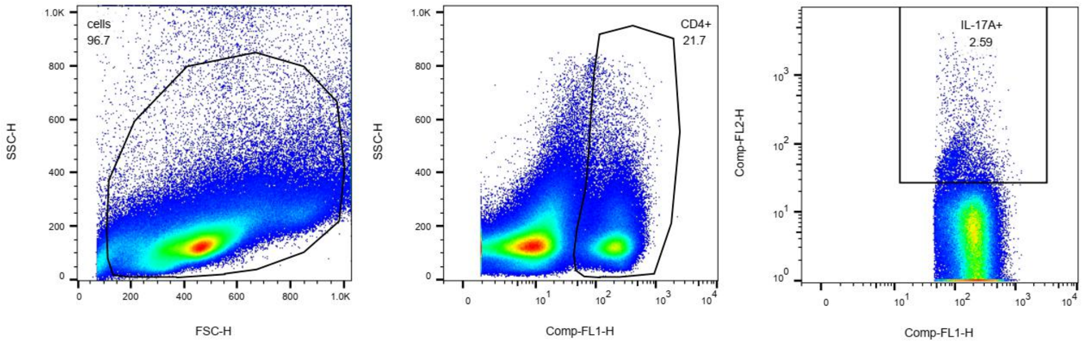

GH

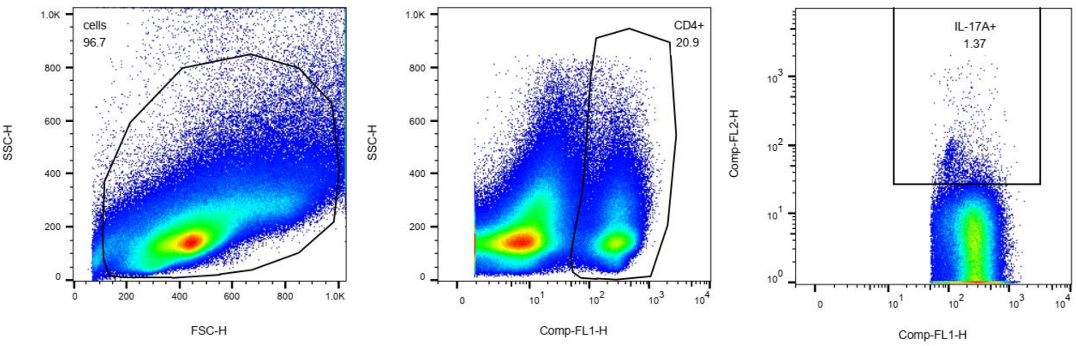

5-ASA

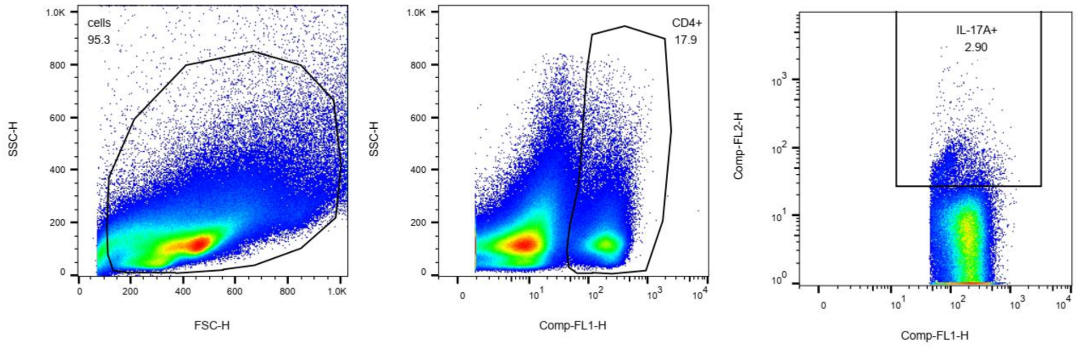

Th17-MLN

Control

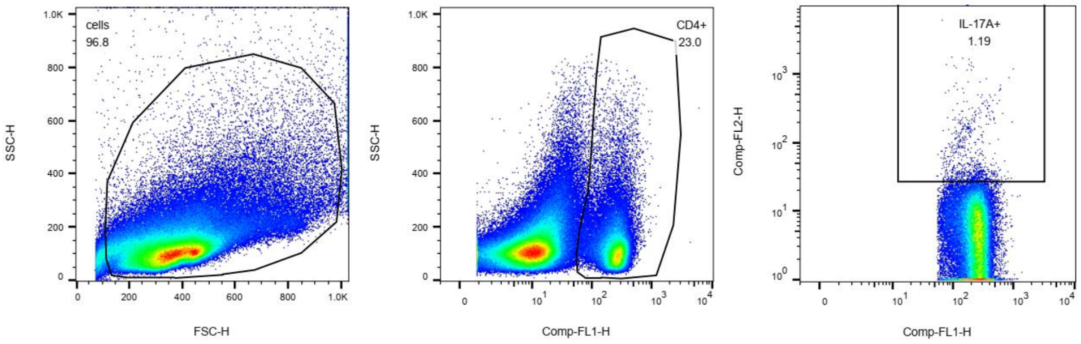

DSS

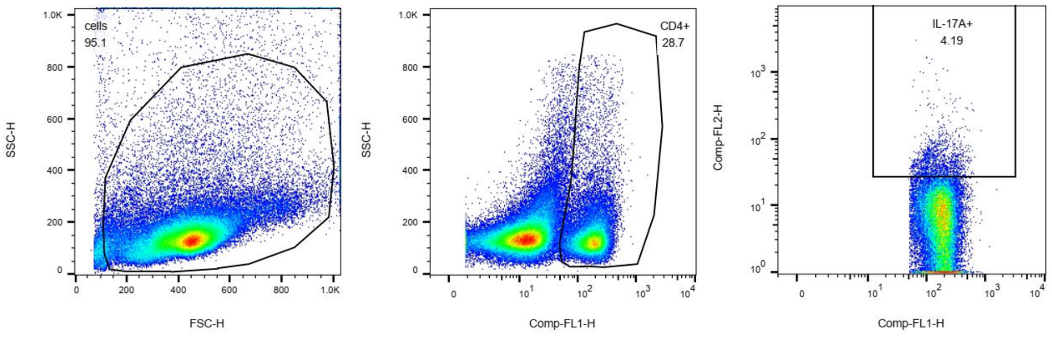

GL

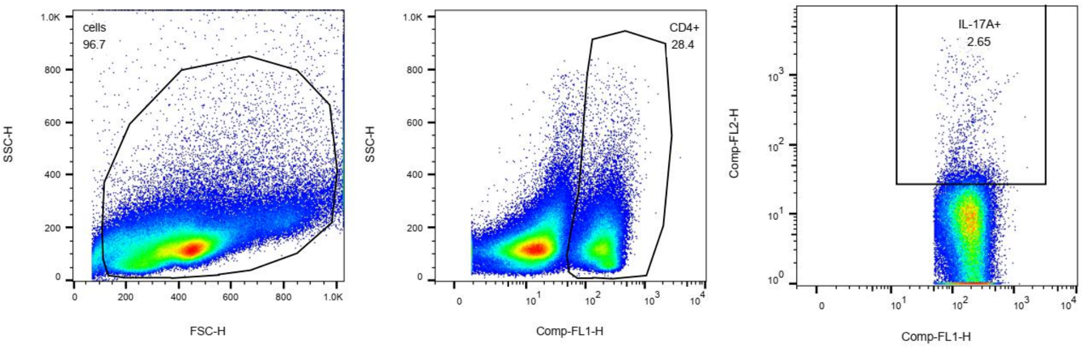
cells
96.7
SSC-H
FSC-H
CD4+
28.4
SSC-H
Comp-FL1-H
IL-17A+
2.65
Comp-FL2-H
Comp-FL1-H

GM

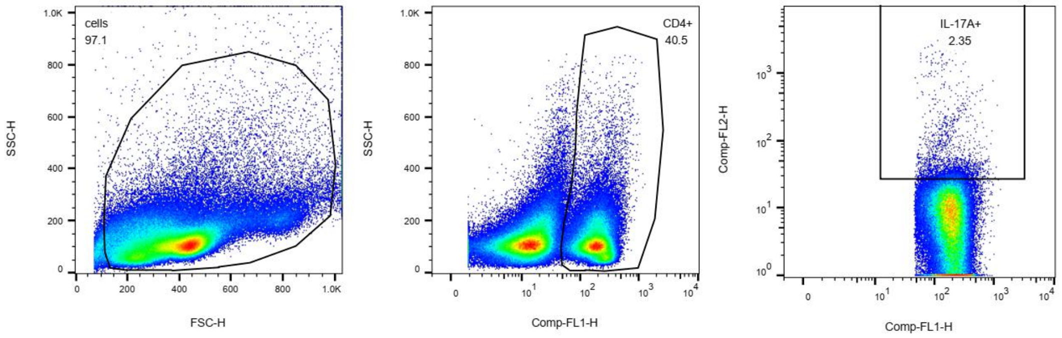
cells
97.1
SSC-H
FSC-H
CD4+
40.5
SSC-H
Comp-FL1-H
IL-17A+
2.35
Comp-FL2-H
Comp-FL1-H

GH

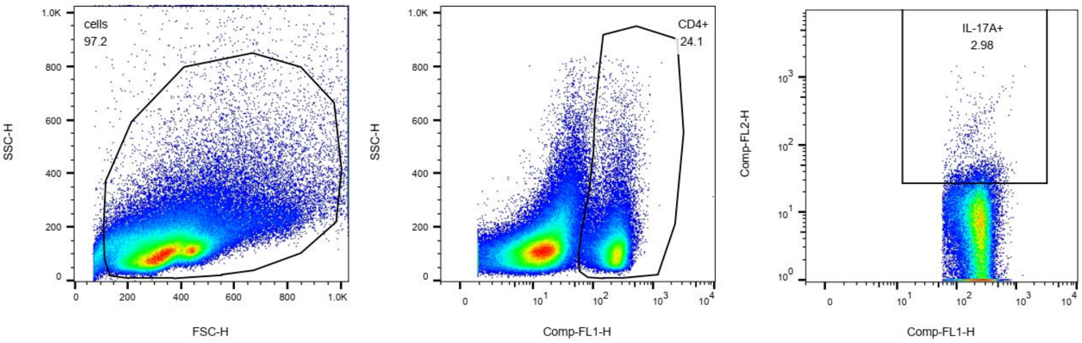
cells
97.2
SSC-H
FSC-H
CD4+
24.1
SSC-H
Comp-FL1-H
IL-17A+
2.98
Comp-FL2-H
Comp-FL1-H

5-ASA

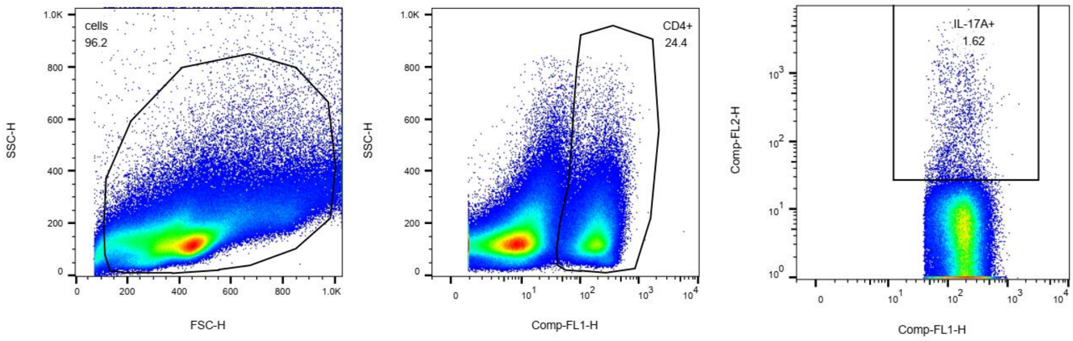
cells
96.2
SSC-H
FSC-H
CD4+
24.4
SSC-H
Comp-FL1-H
IL-17A+
1.62
Comp-FL2-H
Comp-FL1-H

Treg-Spleen

ABX

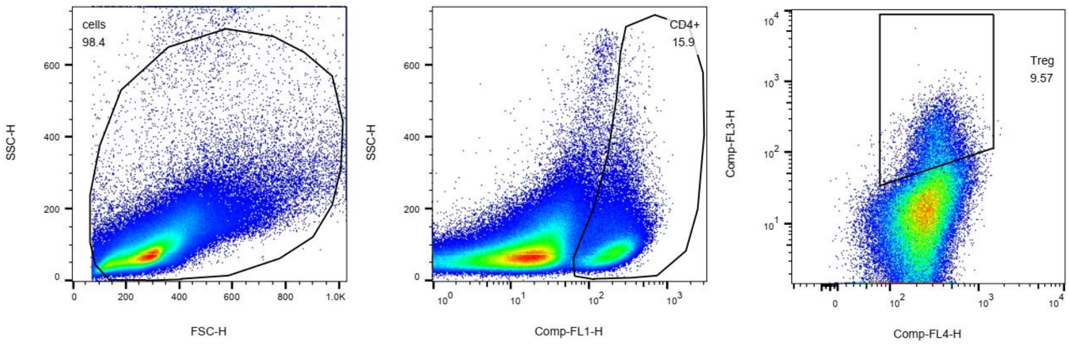

ABS(DSS)

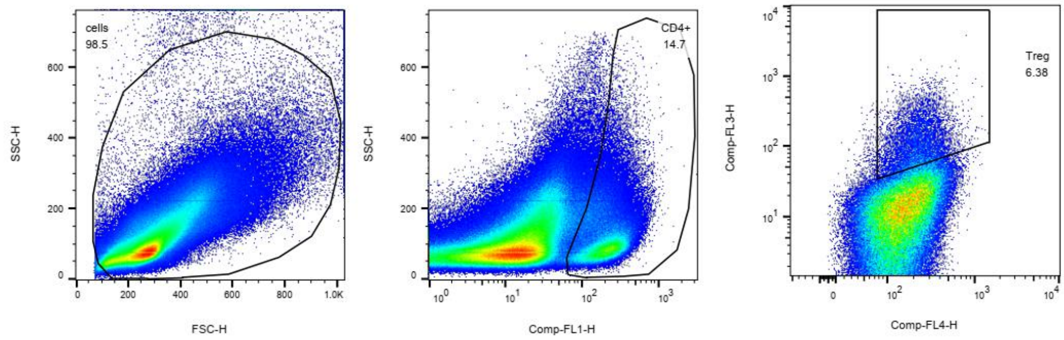

ABX(DSS+GH)

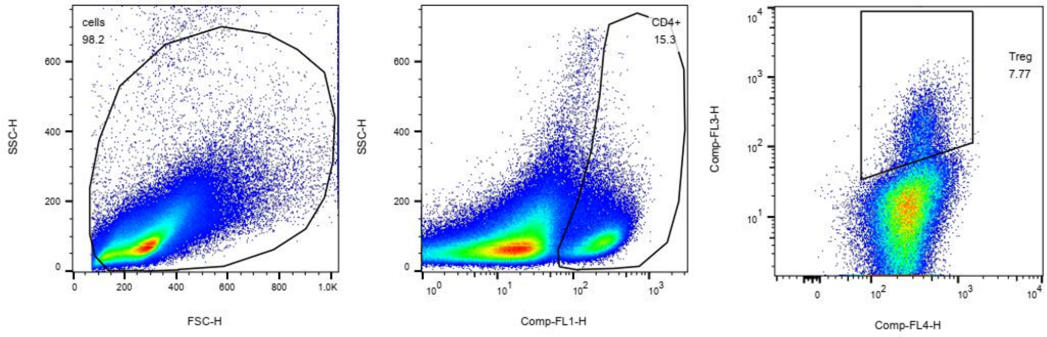

Treg-MLN

ABX

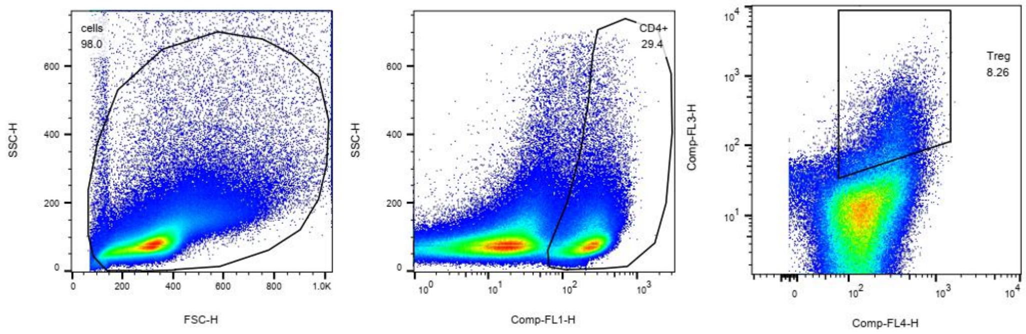

ABX(DSS)

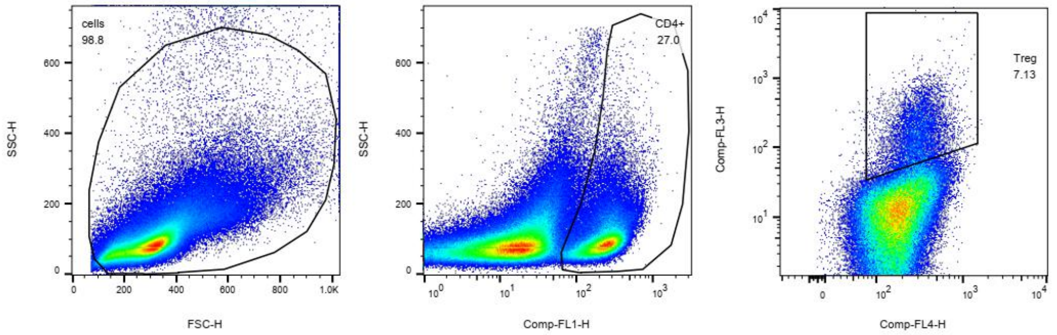

ABX(DSS+GH)

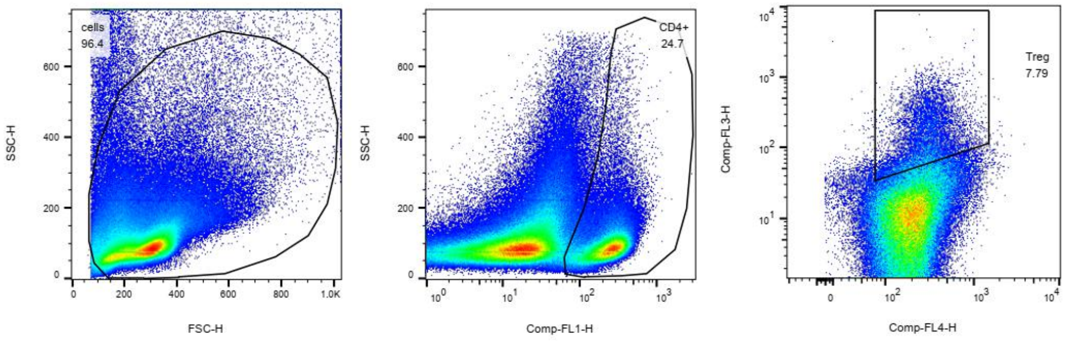

Th17-Spleen

ABX

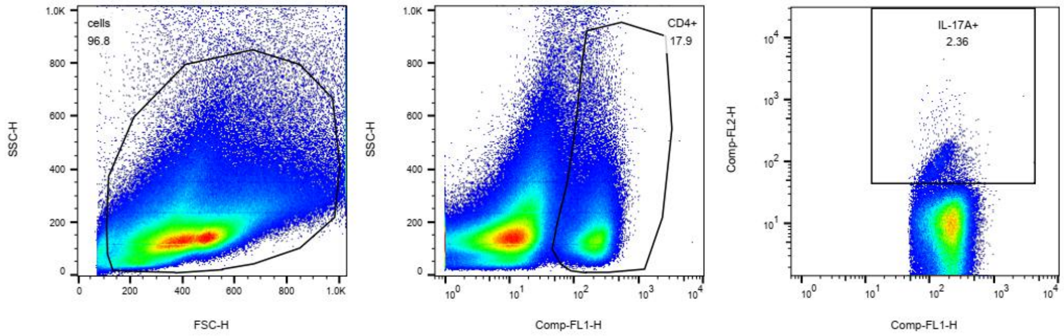

ABX(DSS)

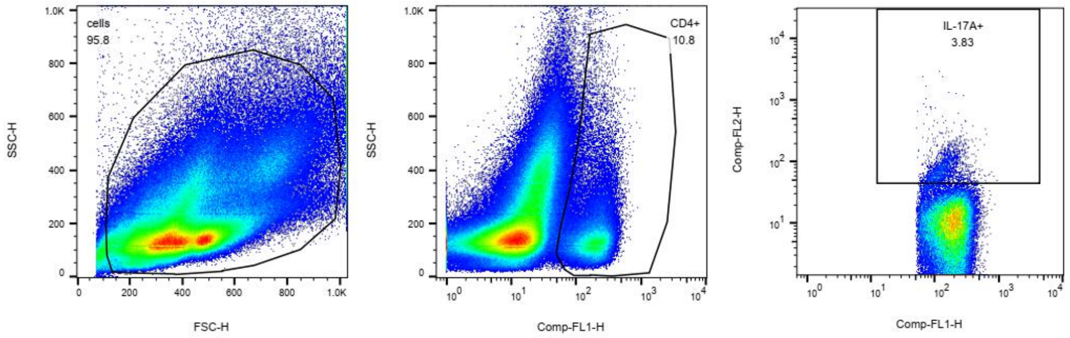

ABX(DSS+GH)

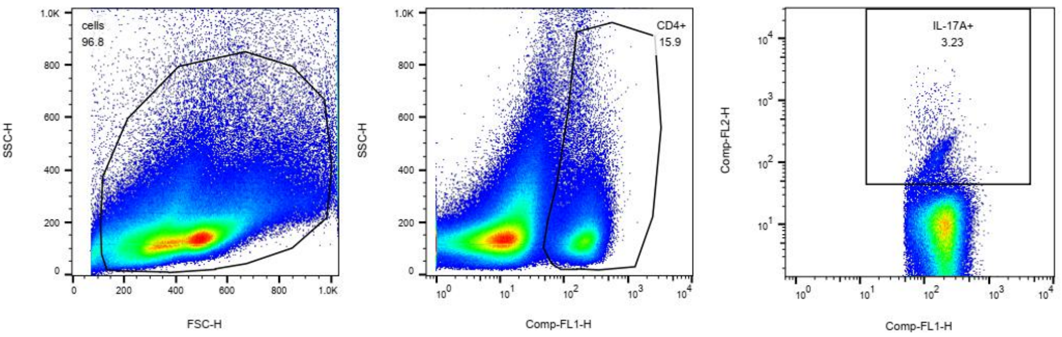

Th17-MLN

ABX

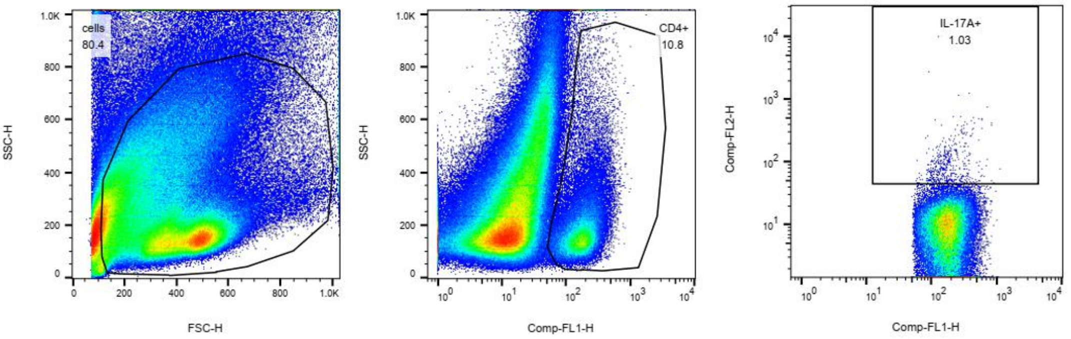

ABX(DSS)

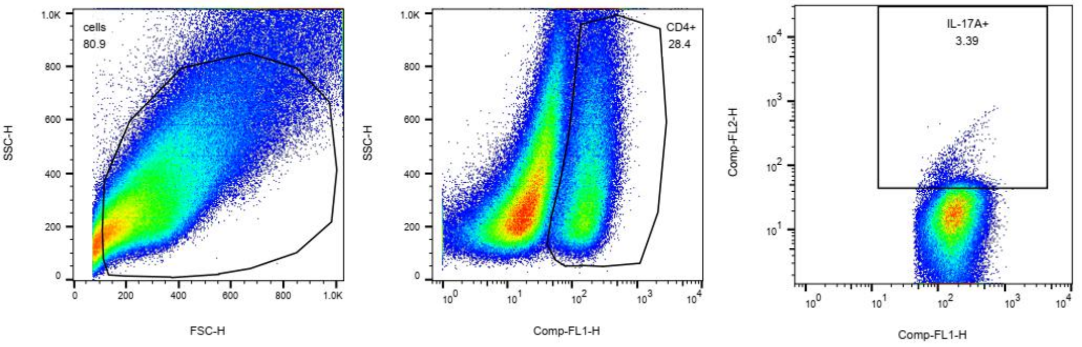

ABX(DSS+GH)

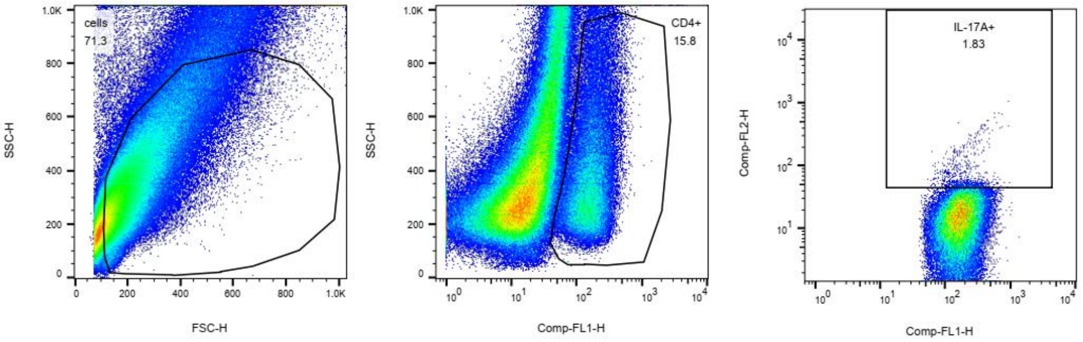

Treg-Spleen

FMTCON

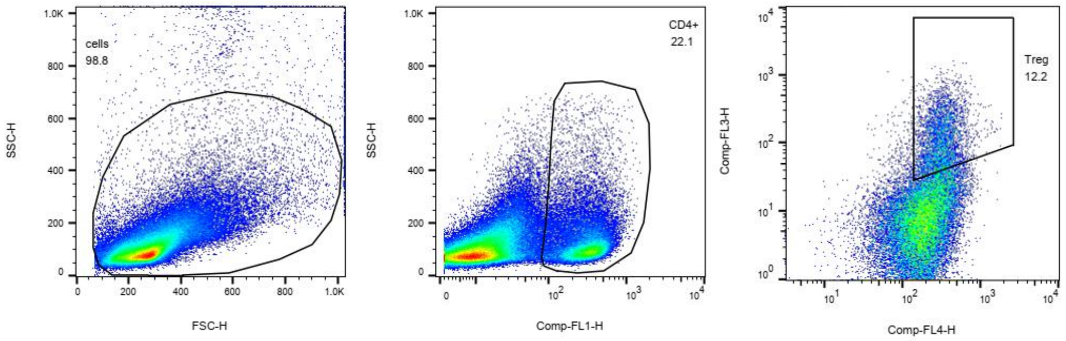

FMTDSS

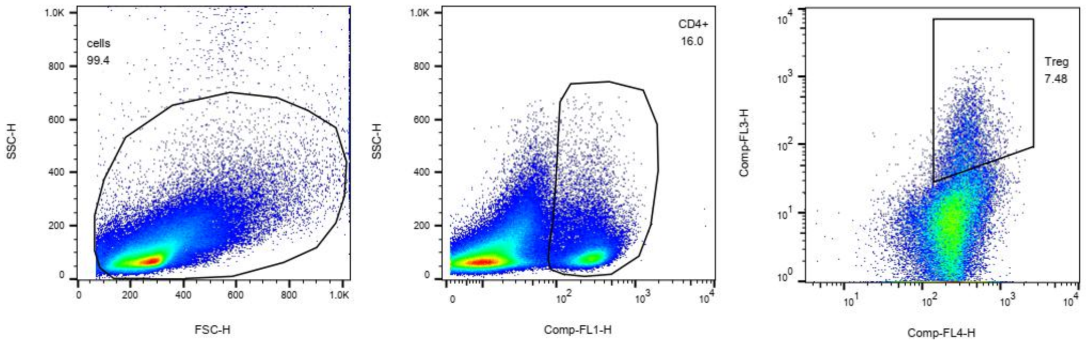

FMTGH

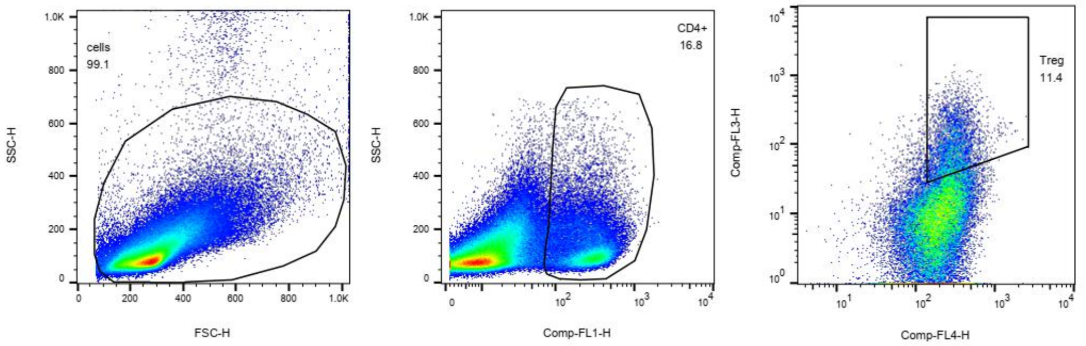

Treg-MLN

FMTCON

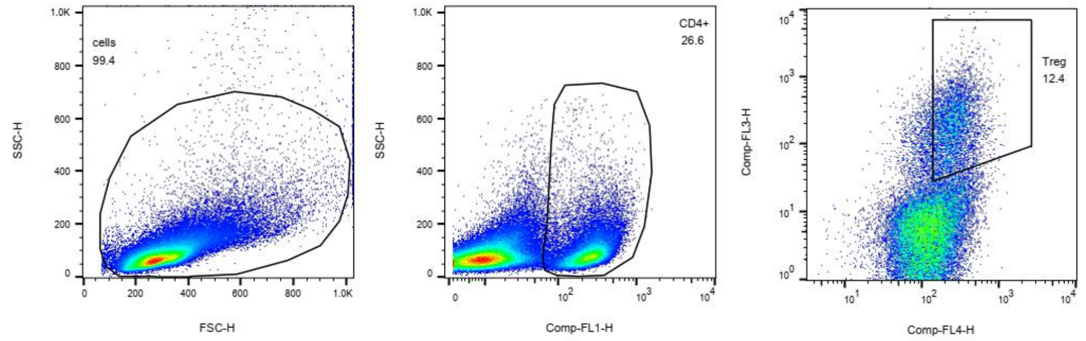

FMTDSS

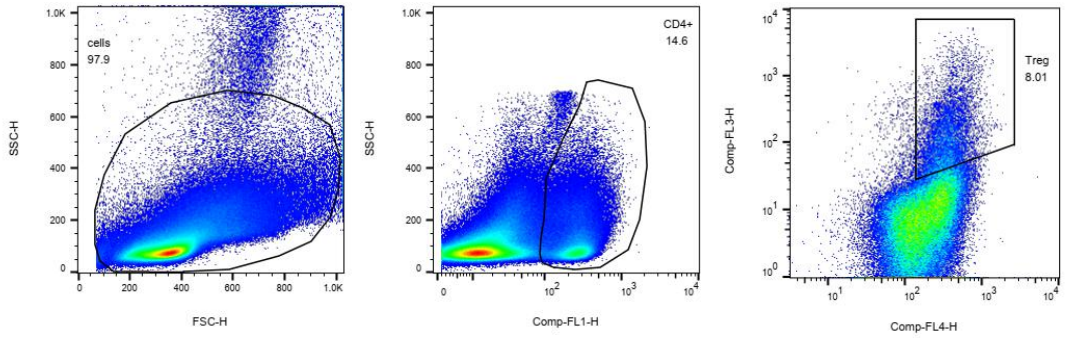

FMTGH

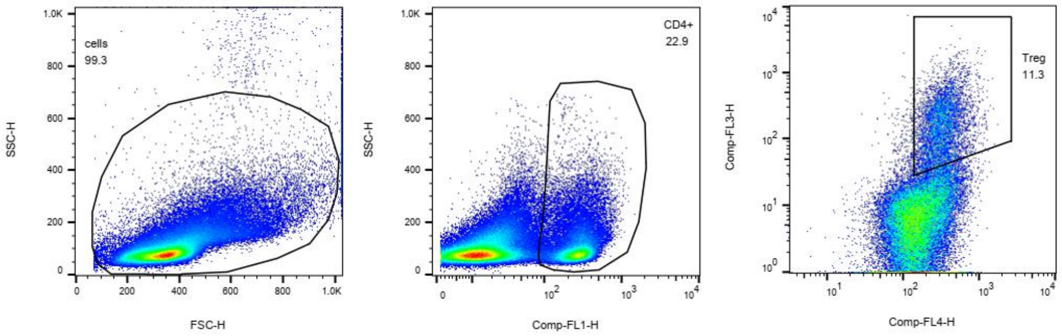

Th17-Spleen

FMTCON

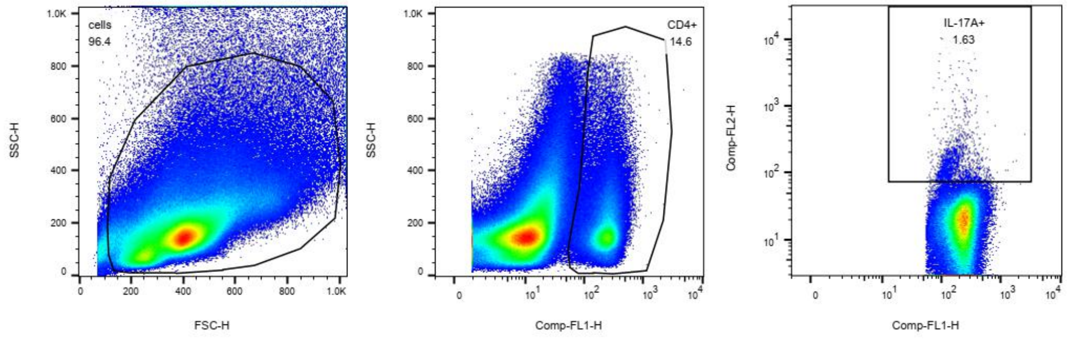

FMTDSS

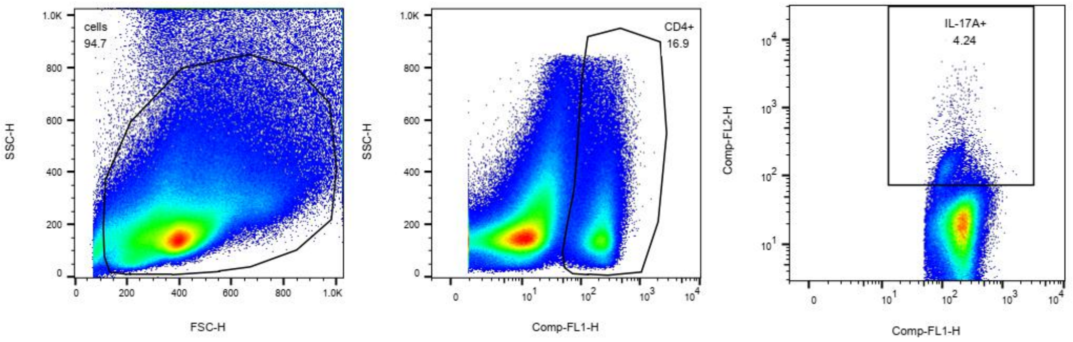

FMTGH

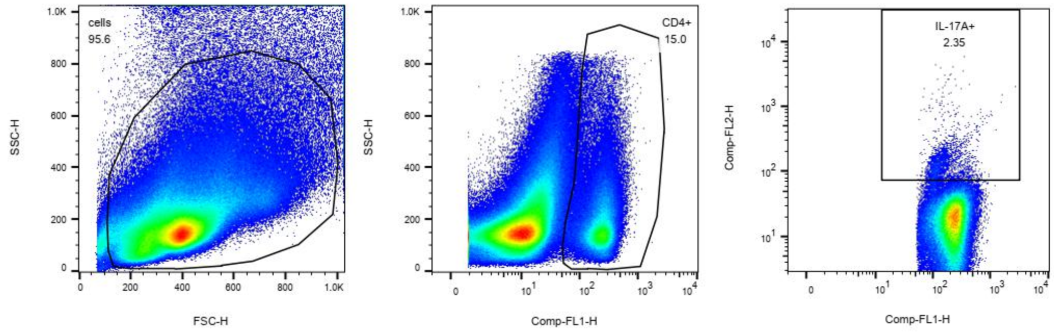

Th17-MLN

FMTCON

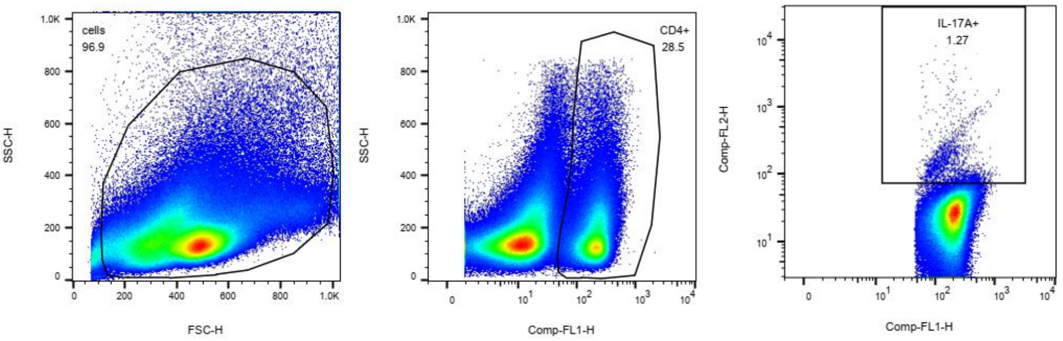

FMTDSS

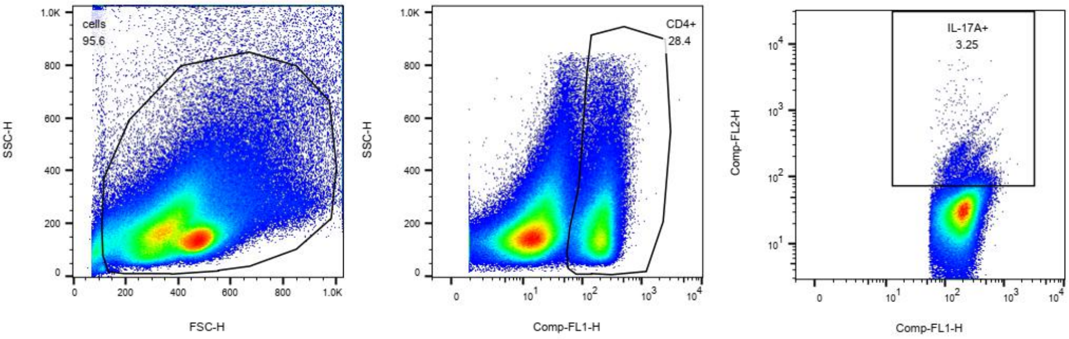

FMTGH

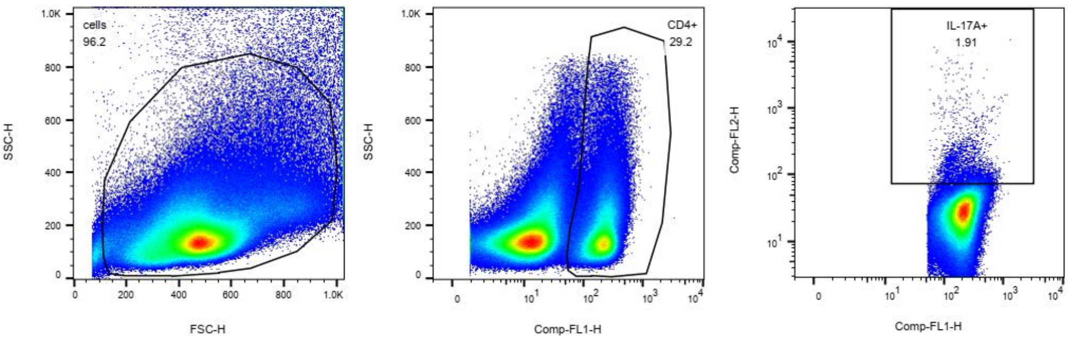

Supplement: Supplementary file 1 [file DataSheet2.pdf]
