# Supplementary figures and images for: Modified Gegen Qinlian Decoction Regulates Treg/Th17 Balance to Ameliorate DSS-Induced Acute Experimental Colitis in Mice by Altering the Gut Microbiota
Source: Front Pharmacol. 2021 Nov 4;12:756978. doi: 10.3389/fphar.2021.756978 (PMC8601377; doi:10.3389/fphar.2021.756978)

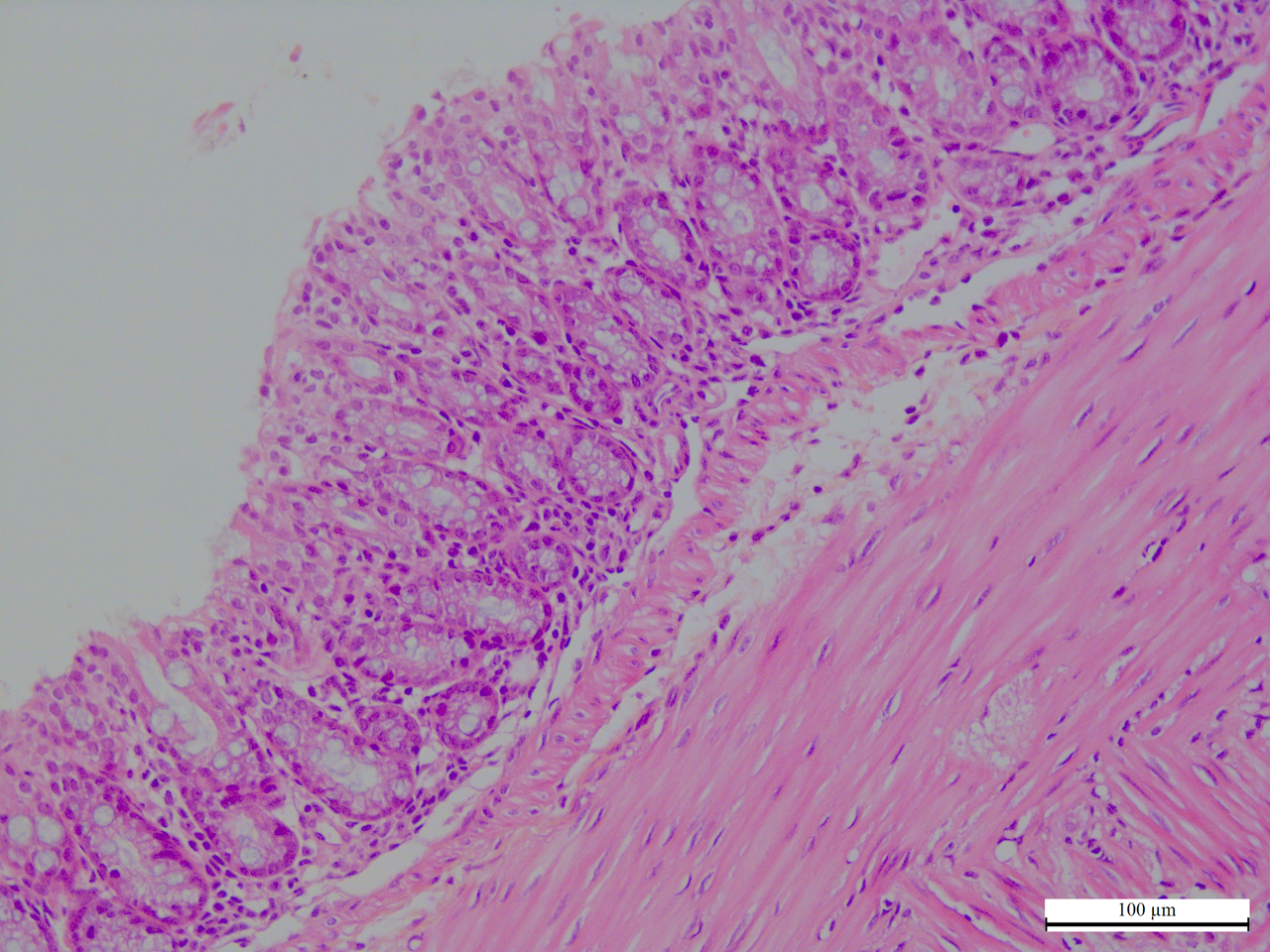

Supplement: Supplementary file 3 [file Image6.TIF]

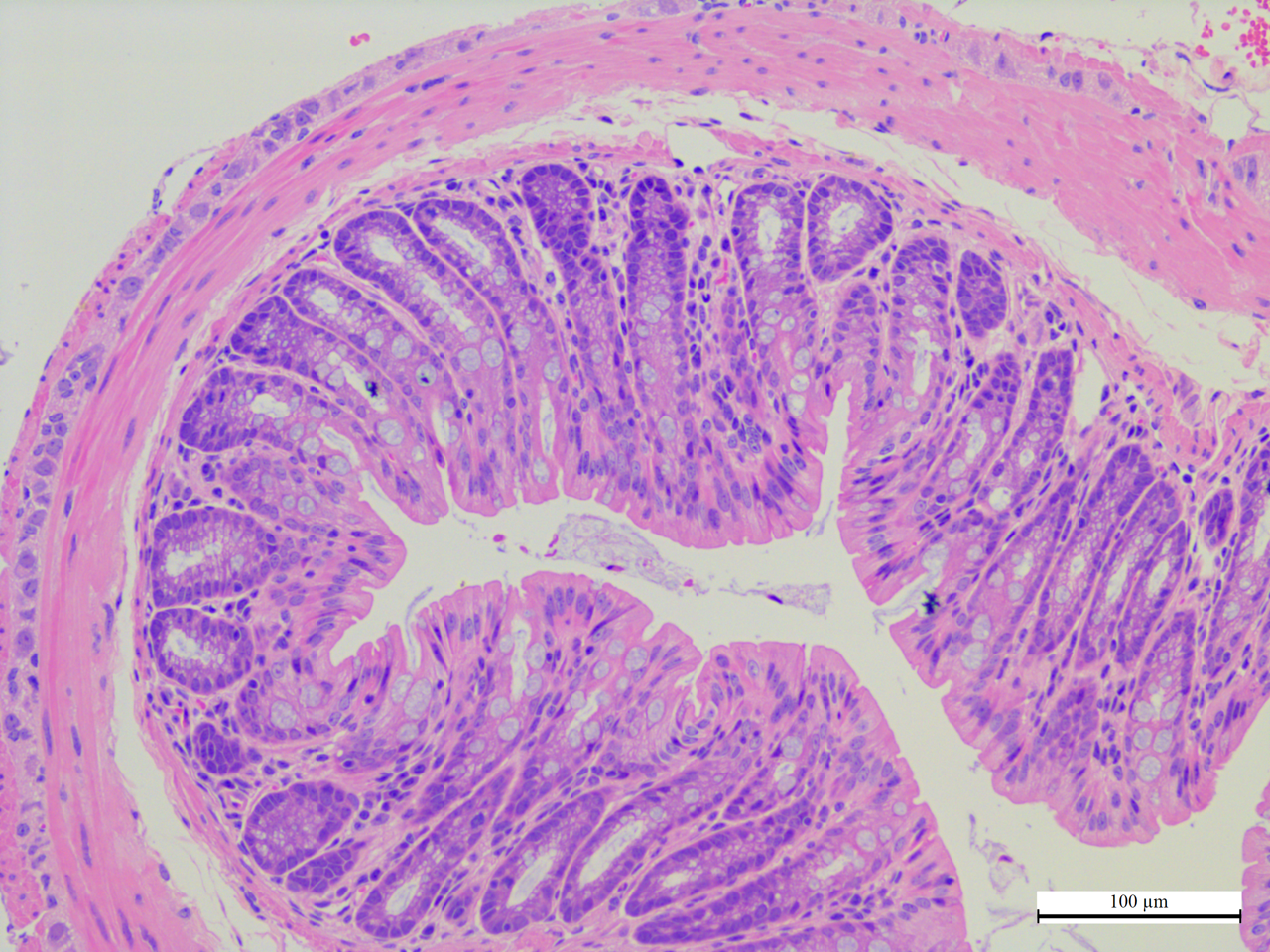

Supplement: Supplementary file 4 [file Image14.TIF]

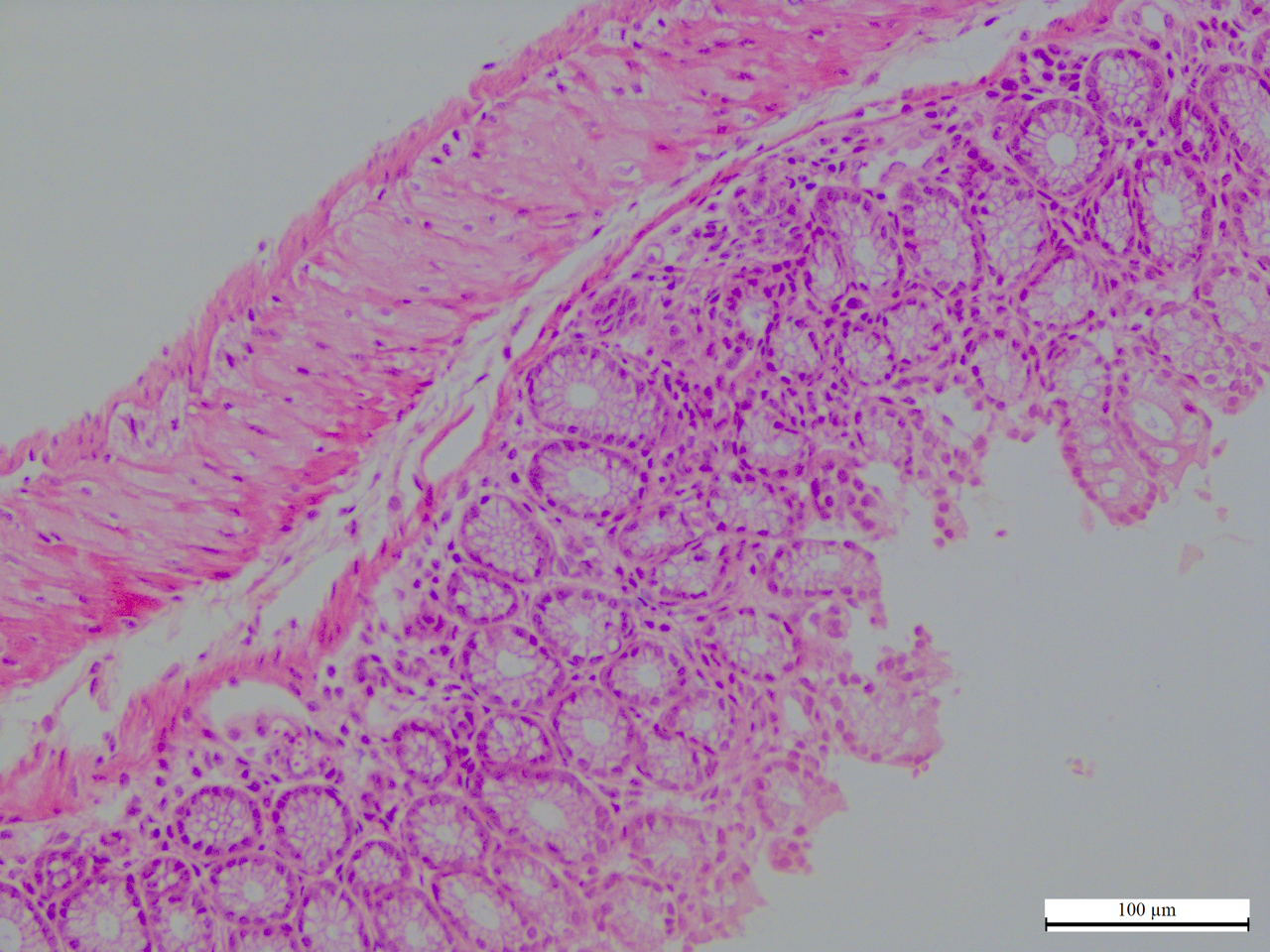

Supplement: Supplementary file 6 [file Image3.TIF]

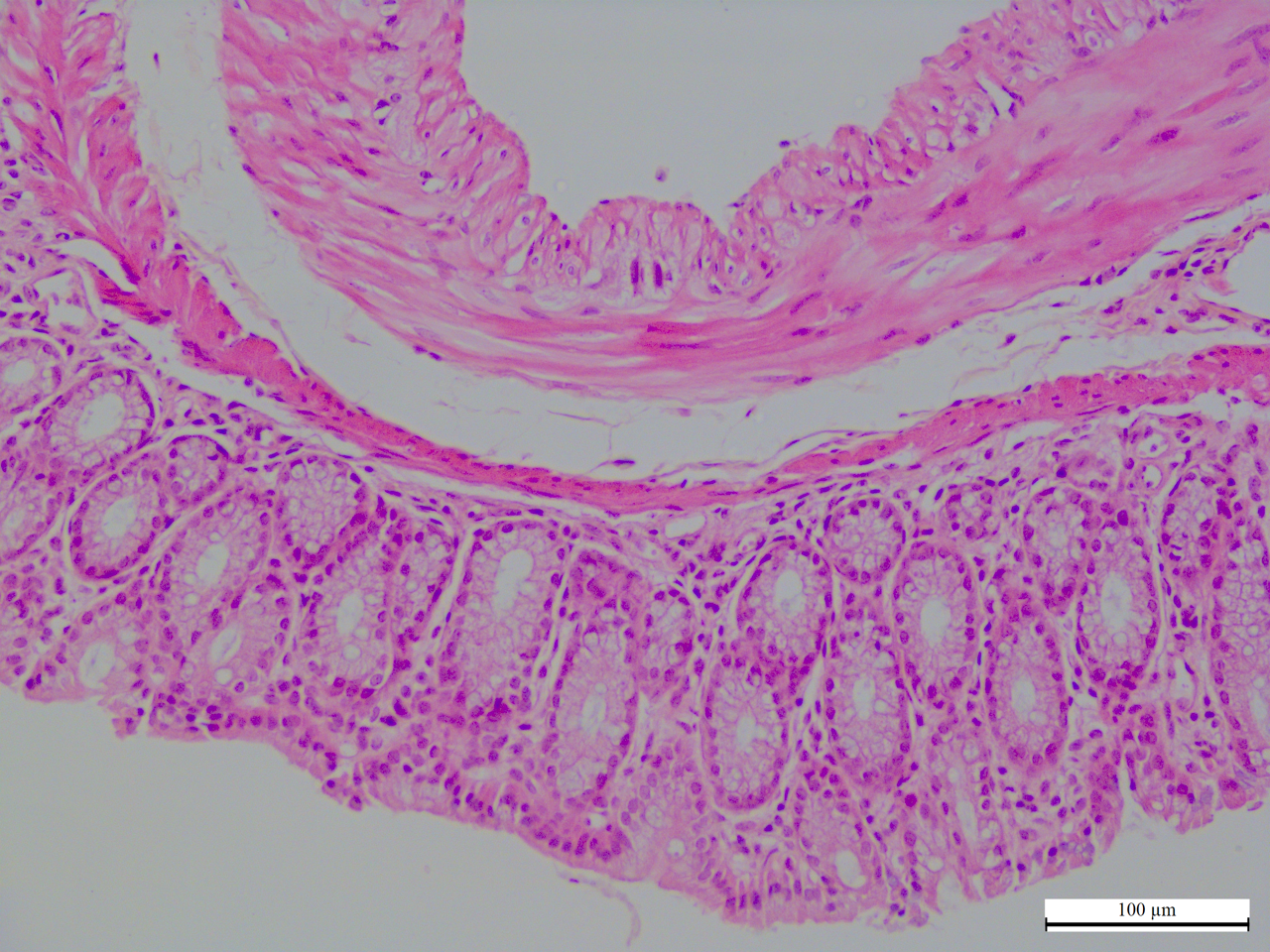

Supplement: Supplementary file 8 [file Image4.TIF]

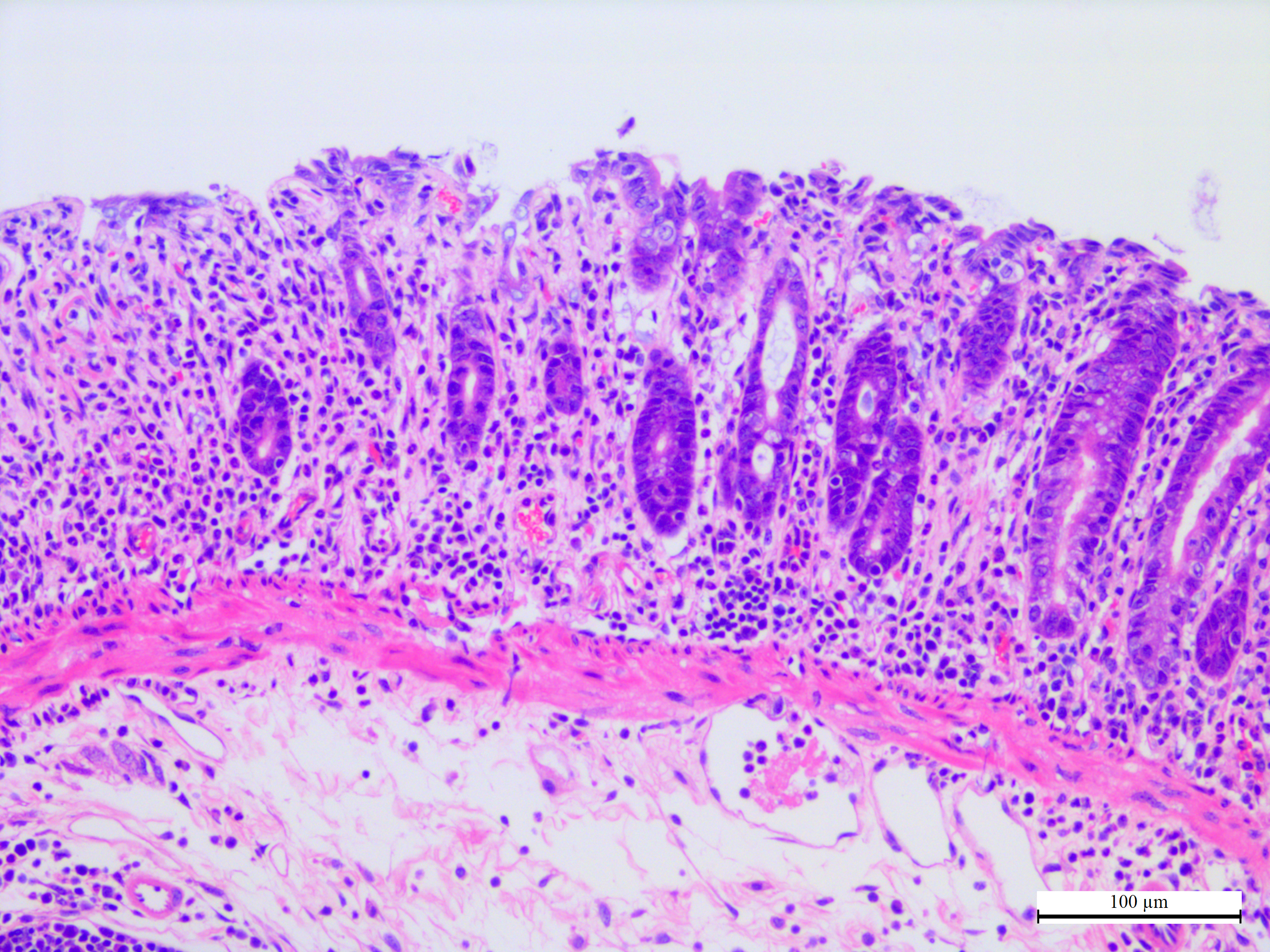

Supplement: Supplementary file 9 [file Image9.TIF]

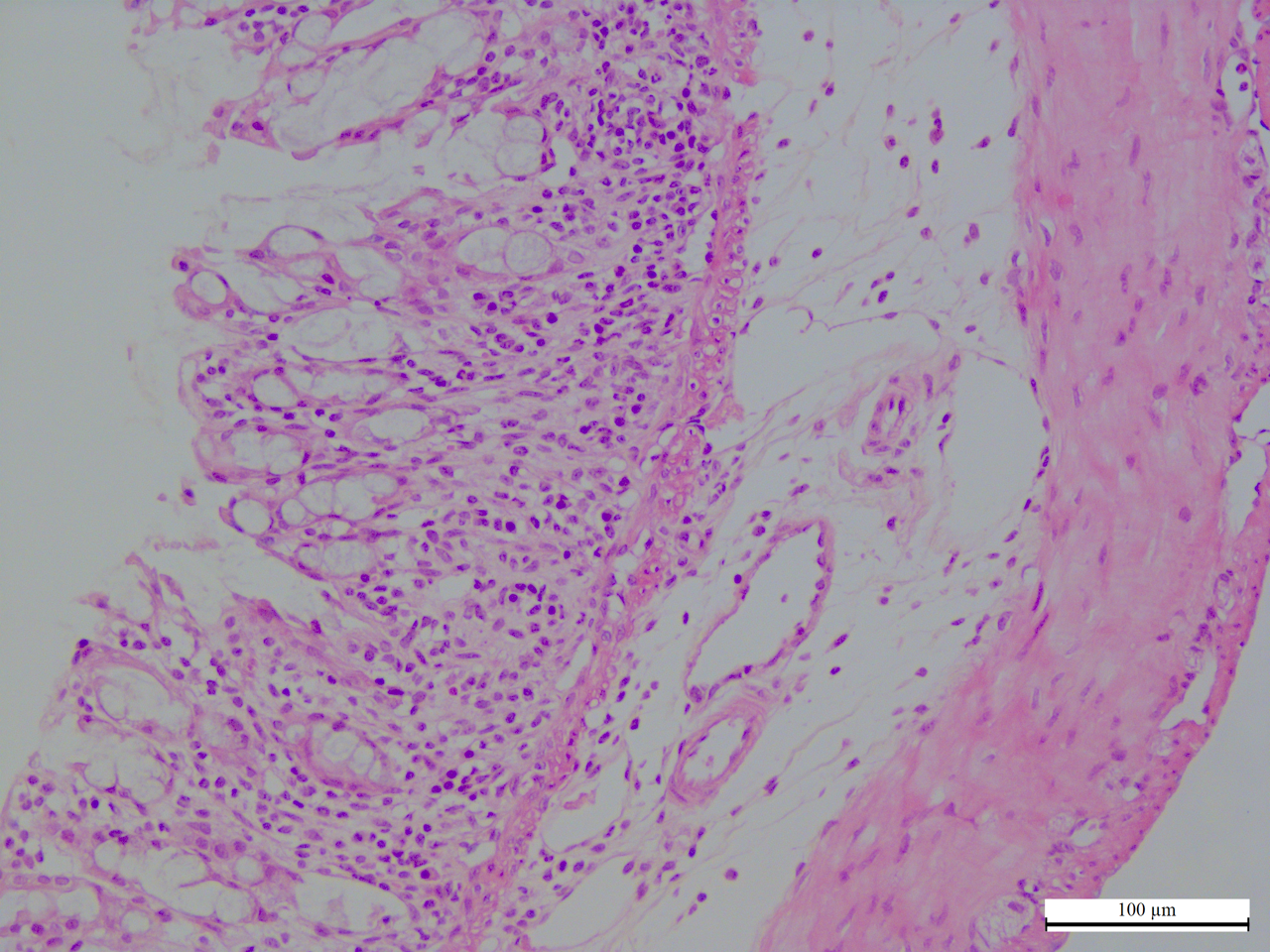

Supplement: Supplementary file 10 [file Image2.TIF]

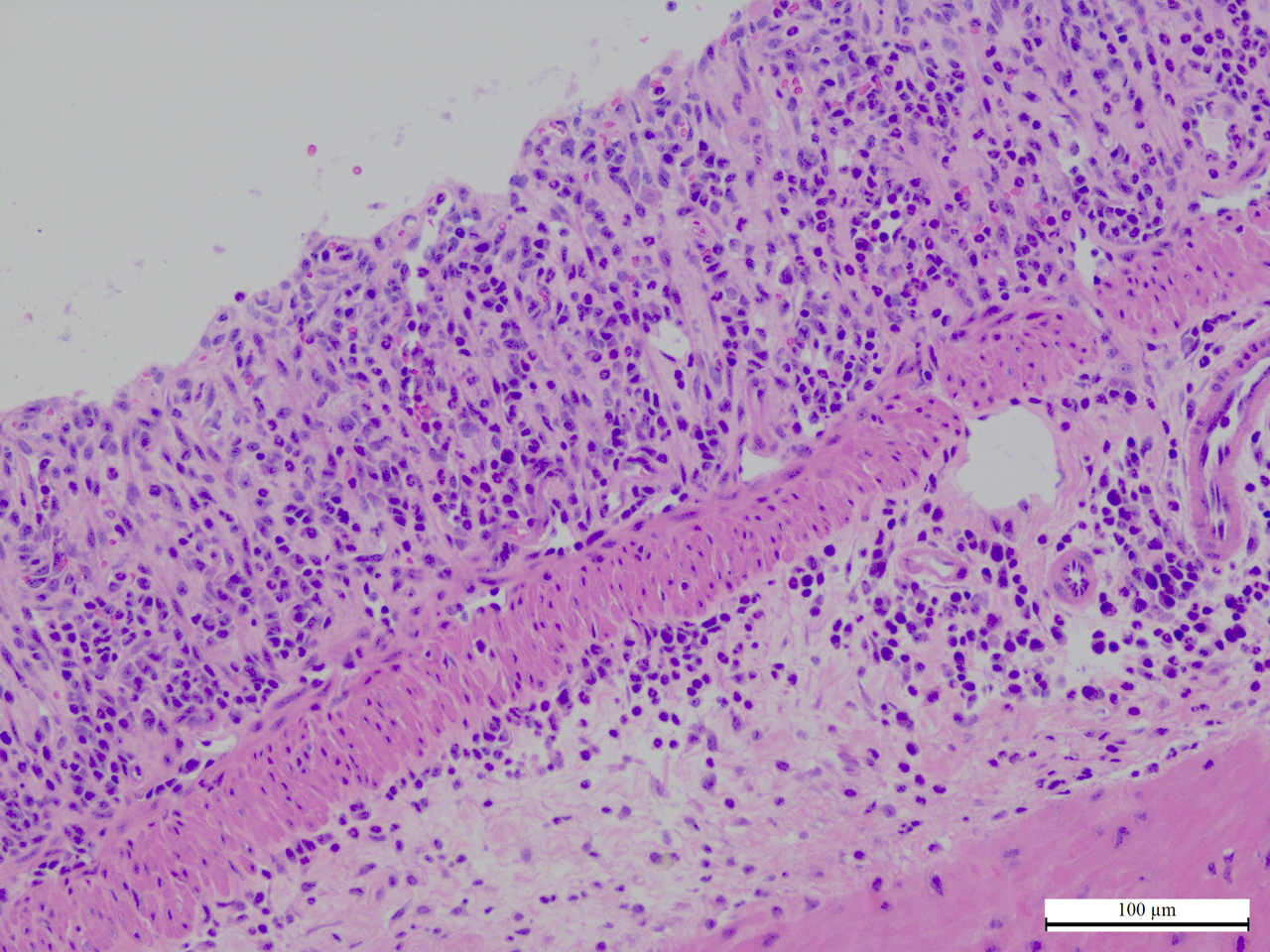

Supplement: Supplementary file 11 [file Image13.TIF]

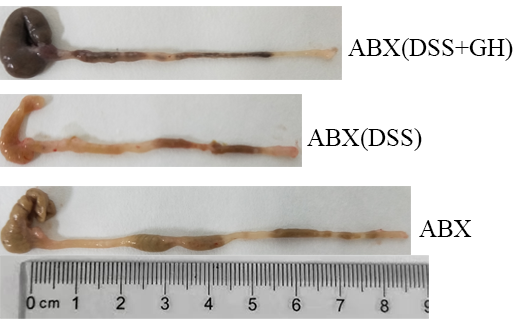

Supplement: Supplementary file 12 [file Image11.TIF]

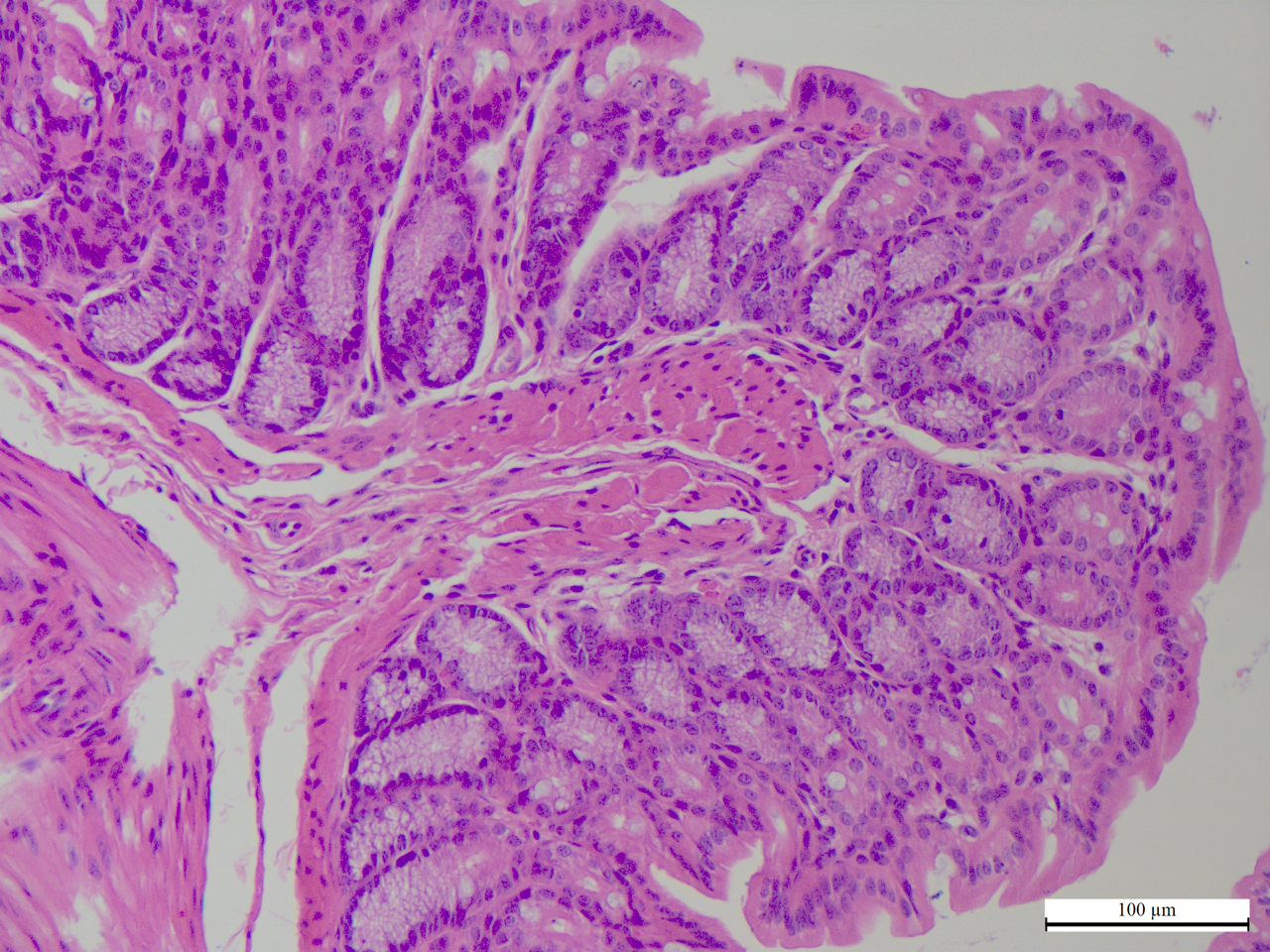

Supplement: Supplementary file 13 [file Image1.TIF]

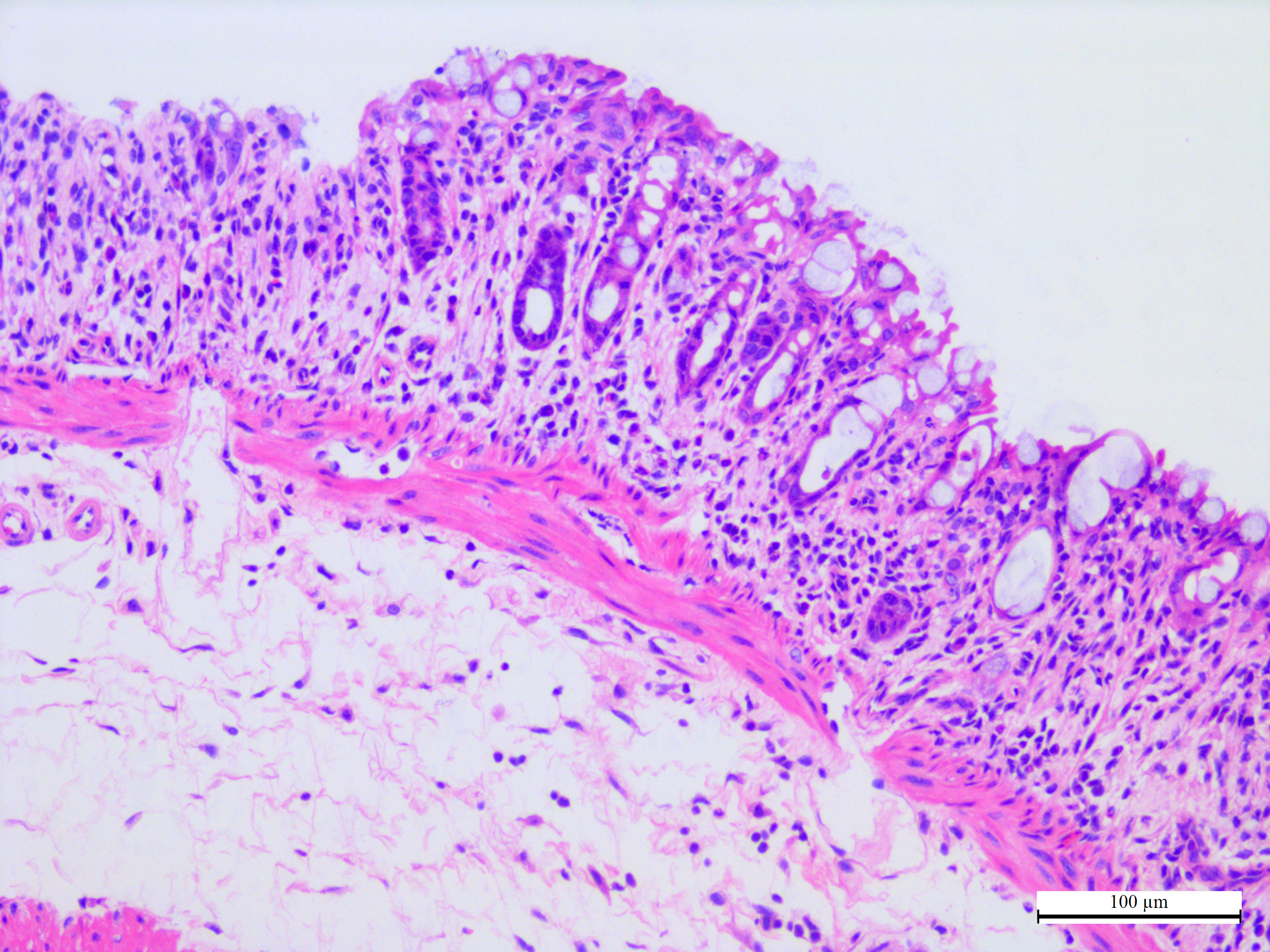

Supplement: Supplementary file 14 [file Image10.TIF]

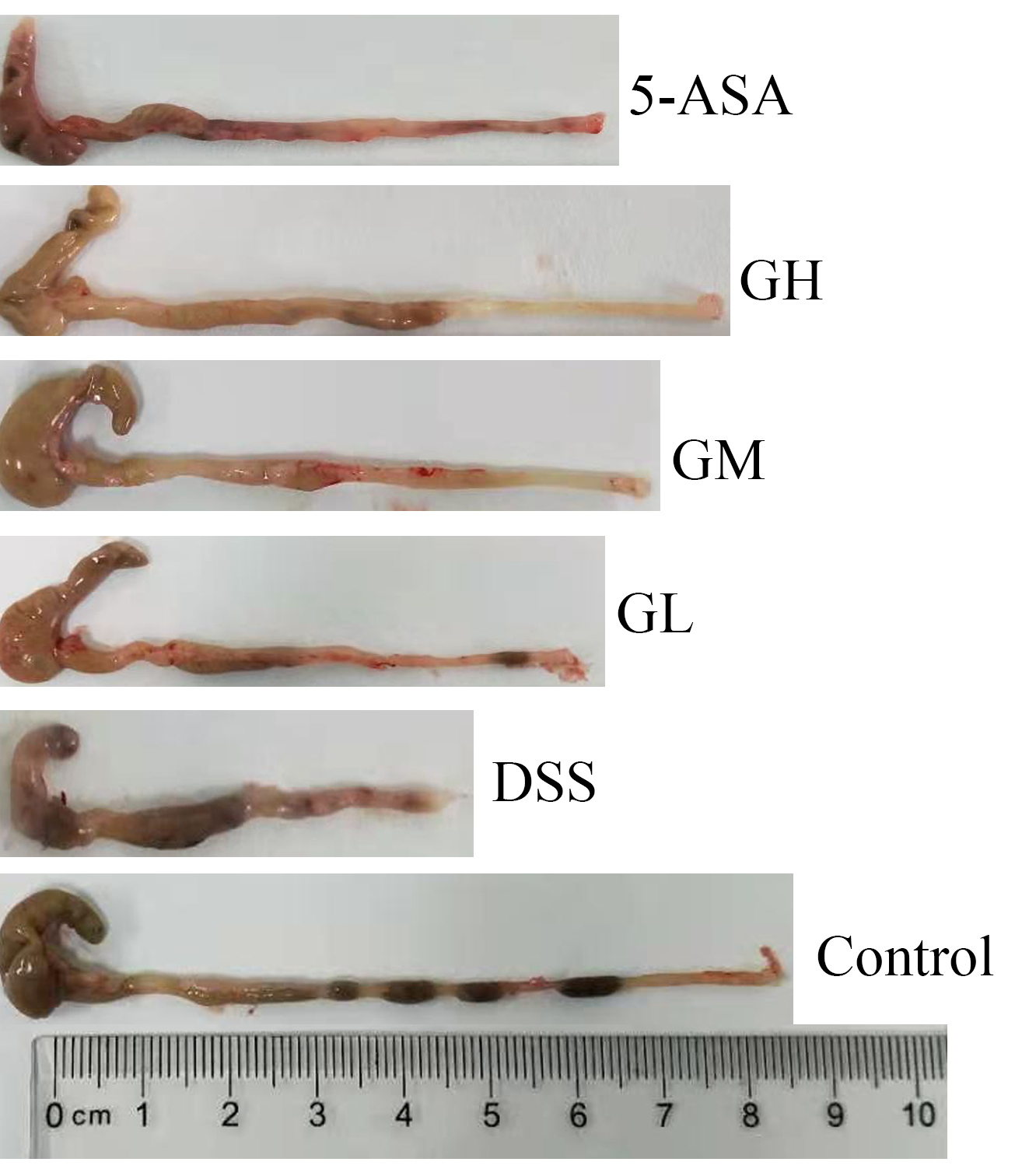

Supplement: Supplementary file 15 [file Image7.TIF]

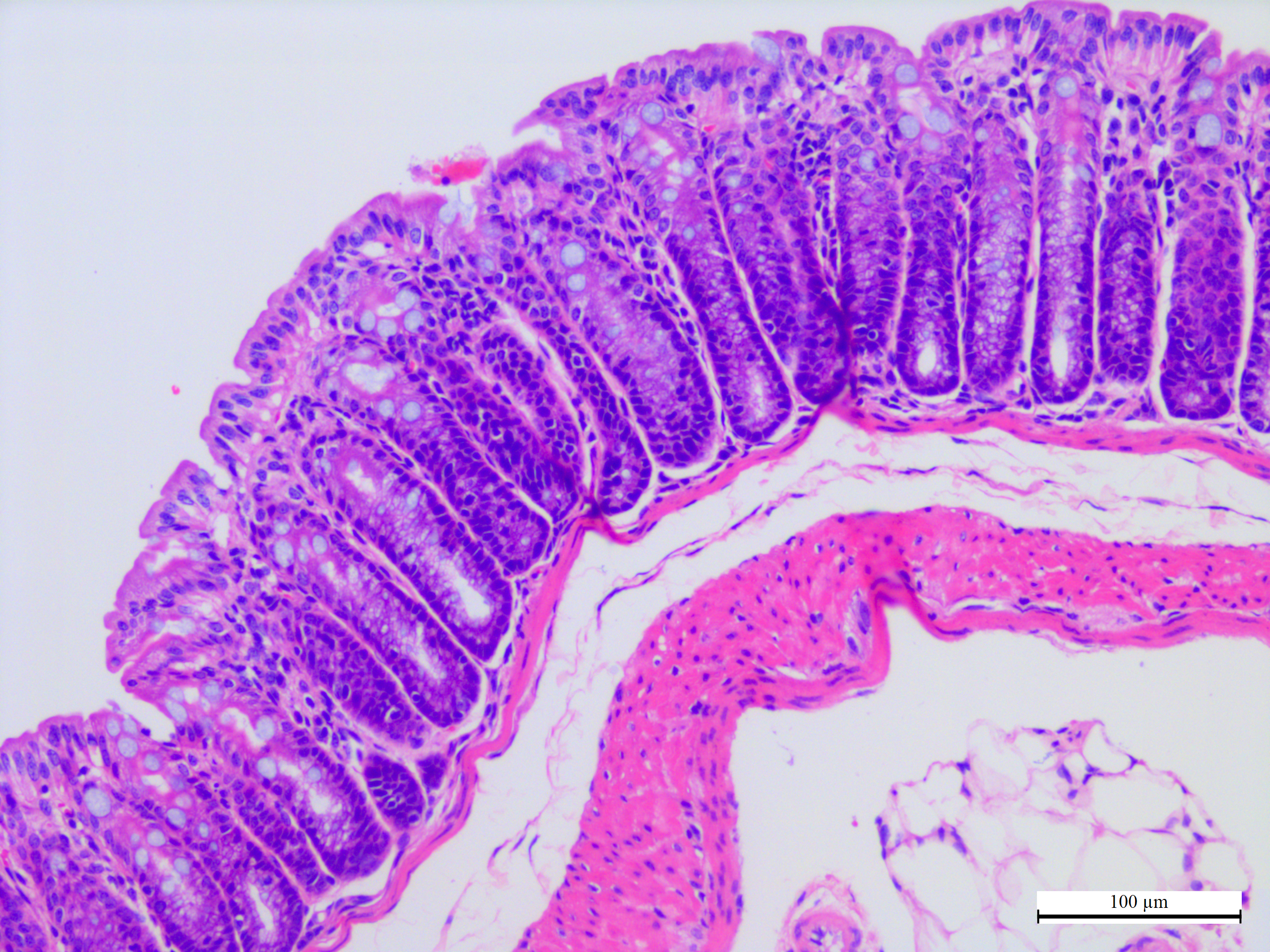

Supplement: Supplementary file 18 [file Image8.TIF]

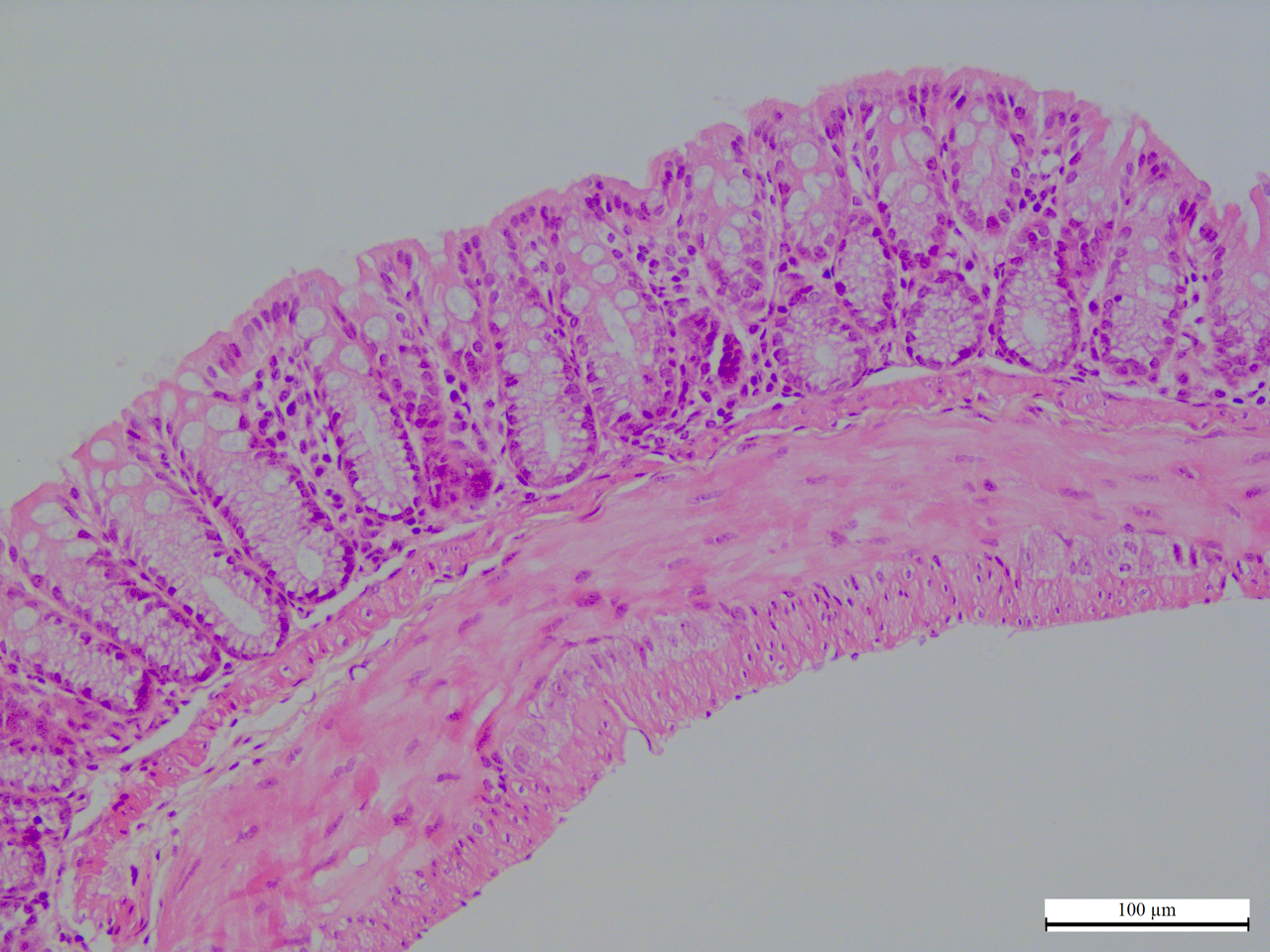

Supplement: Supplementary file 20 [file Image5.TIF]

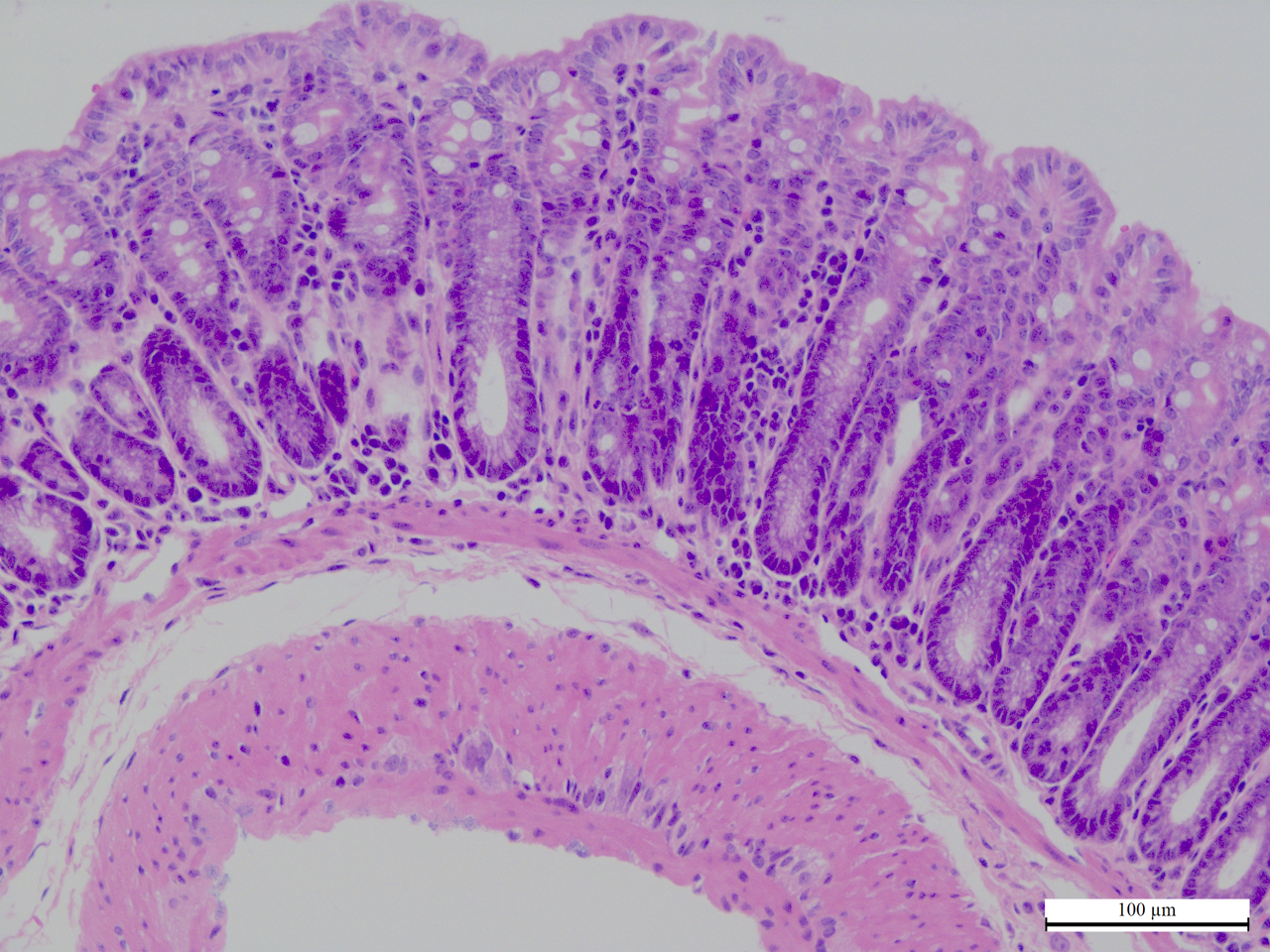

Supplement: Supplementary file 21 [file Image12.TIF]
