# Supplementary material for: Modified Gegen Qinlian Decoction Regulates Treg/Th17 Balance to Ameliorate DSS-Induced Acute Experimental Colitis in Mice by Altering the Gut Microbiota
Source: Front Pharmacol. 2021 Nov 4;12:756978. doi: 10.3389/fphar.2021.756978 (PMC8601377; doi:10.3389/fphar.2021.756978)
Supplement: Supplementary file 17 [file DataSheet1.PDF]

Supplementary Materials

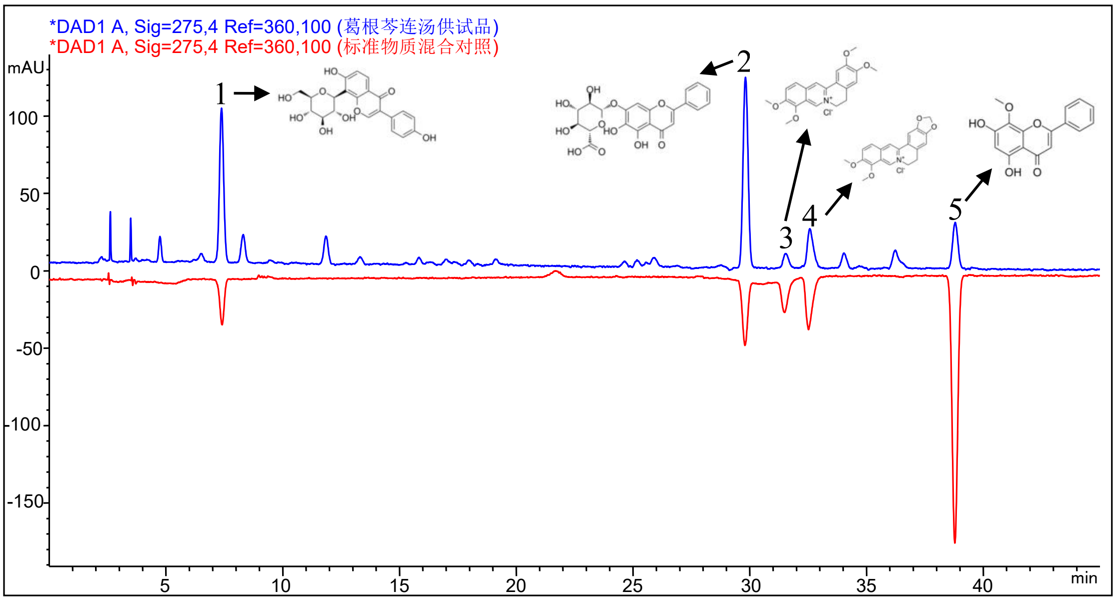

1=Puerarin; 2=Baicalin; 3=Palmatine hydrochloride; 4=Berberine hydrochloride; 5=Wogonin

Supplementary Figure 1: High performance liquid chromatography (HPLC) profiles of the main active components of modified Gegen Qinlian decoction.

A

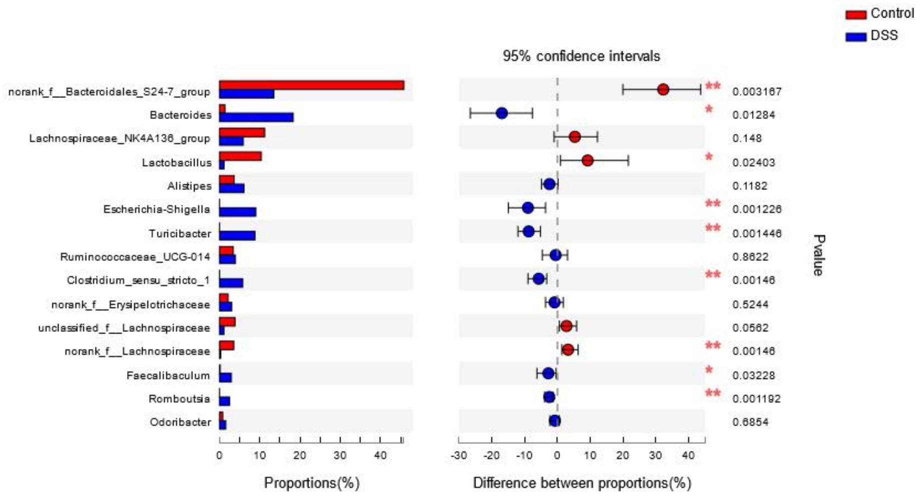
Control
DSS
95% confidence intervals
norank_f__Bacteroidales_S24-7_group
Bacteroides
Lachnospiraceae_NK4A136_group
Lactobacillus
Alistipes
Escherichia-Shigella
Turicibacter
Ruminococcaceae_UCG-014
Clostridium_sensu_stricto_1
norank_f__Erysipelotrichaceae
unclassified_f__Lachnospiraceae
norank_f__Lachnospiraceae
Faecalibaculum
Romboutsia
Odoribacter
0.003167
0.01284
0.148
0.02403
0.1182
0.001226
0.001446
0.8622
0.00146
0.5244
0.0562
0.00146
0.03228
0.001192
0.6854
P value
Proportions(%)
Difference between proportions(%)

B

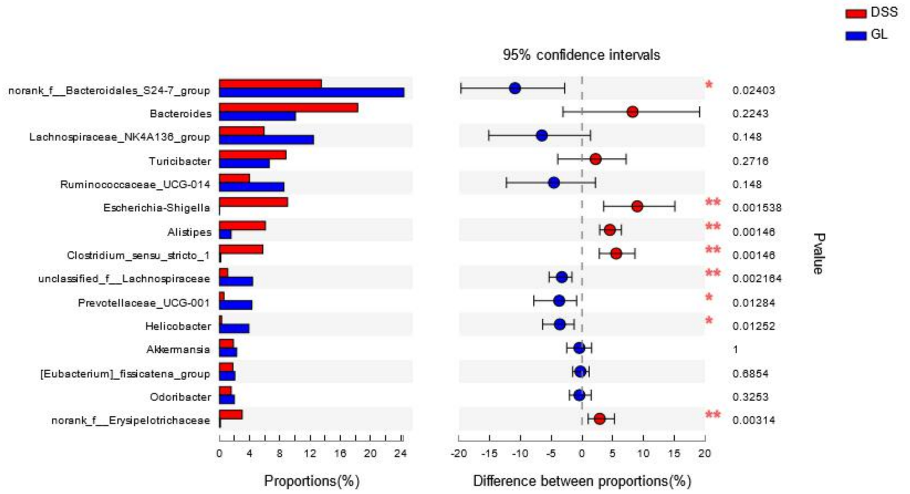
DSS
GL
95% confidence intervals
norank_f__Bacteroidales_S24-7_group
Bacteroides
Lachnospiraceae_NK4A136_group
Turicibacter
Ruminococcaceae_UCG-014
Escherichia-Shigella
Alistipes
Clostridium_sensu_stricto_1
unclassified_f__Lachnospiraceae
Prevotellaceae_UCG-001
Helicobacter
Akkermansia
[Eubacterium]_fissicatena_group
Odoribacter
norank_f__Erysipelotrichaceae
0.02403
0.2243
0.148
0.2716
0.148
0.001538
0.00146
0.00146
0.002164
0.01284
0.01252
1
0.6854
0.3253
0.00314
P value
Proportions(%)
Difference between proportions(%)

C

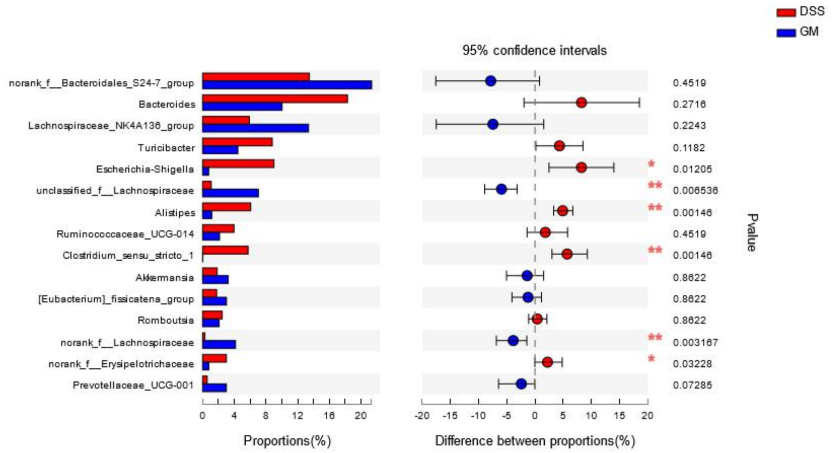
DSS
GM
95% confidence intervals
norank_f__Bacteroidales_S24-7_group
Bacteroides
Lachnospiraceae_NK4A136_group
Turicibacter
Escherichia-Shigella
unclassified_f__Lachnospiraceae
Alistipes
Ruminococcaceae_UCG-014
Clostridium_sensu_stricto_1
Akkermansia
[Eubacterium]_fissicatena_group
Romboutsia
norank_f__Lachnospiraceae
norank_f__Erysipelotrichaceae
Prevotellaceae_UCG-001
0.4519
0.2716
0.2243
0.1182
0.01205
0.006536
0.00146
0.4519
0.00146
0.8622
0.8622
0.8622
0.003167
0.03228
0.07285
P value
Proportions(%)
Difference between proportions(%)

D

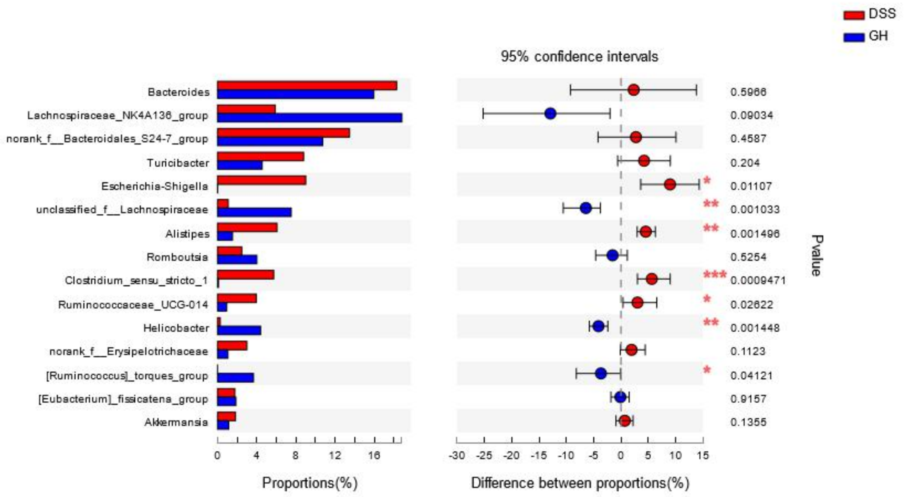

E

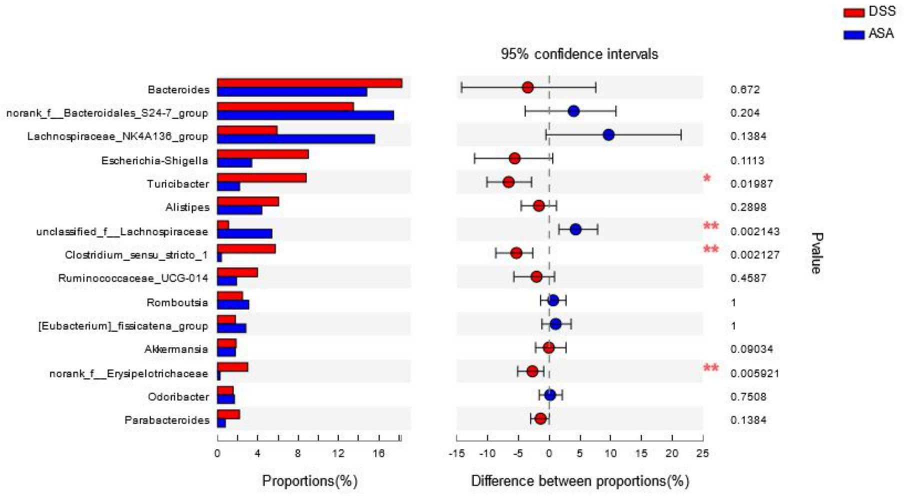

Supplementary Figure 2: Pairwise comparisons of gut microbiota compositions at the genus level between (**A**) Control and DSS (**B**) DSS and GL (**C**) DSS and GM (**D**) DSS and GH (**E**) DSS and 5-ASA. $^{*}p < 0.05$, $^{**}p < 0.01$, $^{***}p < 0.001$.

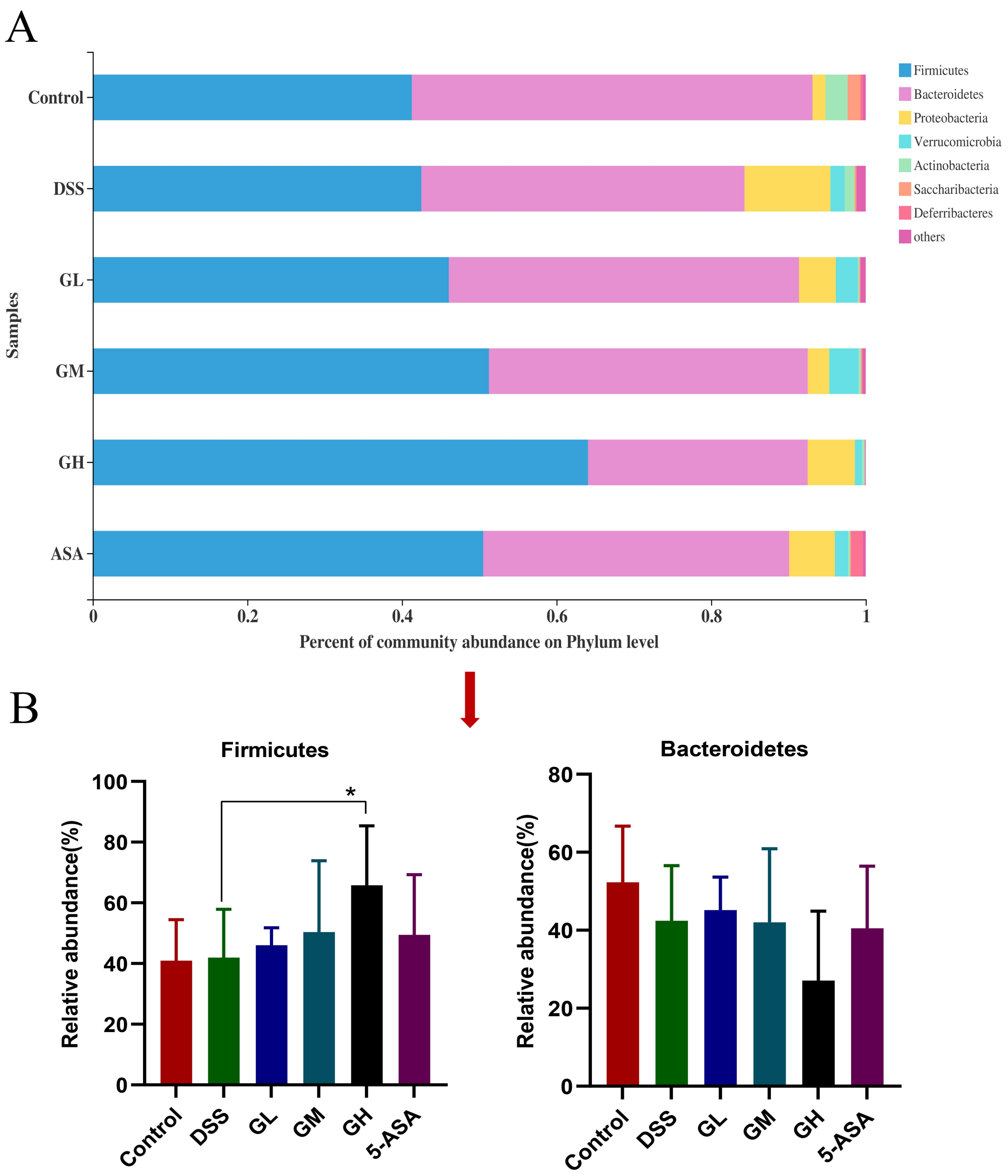

Supplementary Figure 3: Relative abundance of microbial community at the phylum level. (**A**) Bar plots of the phylum taxonomic levels. (**B**) The relative abundances of Firmicutes and Bacteroidetes. Results were shown as mean ± SD (n=6-10). $^{*}p< 0.05$ vs DSS group.

A

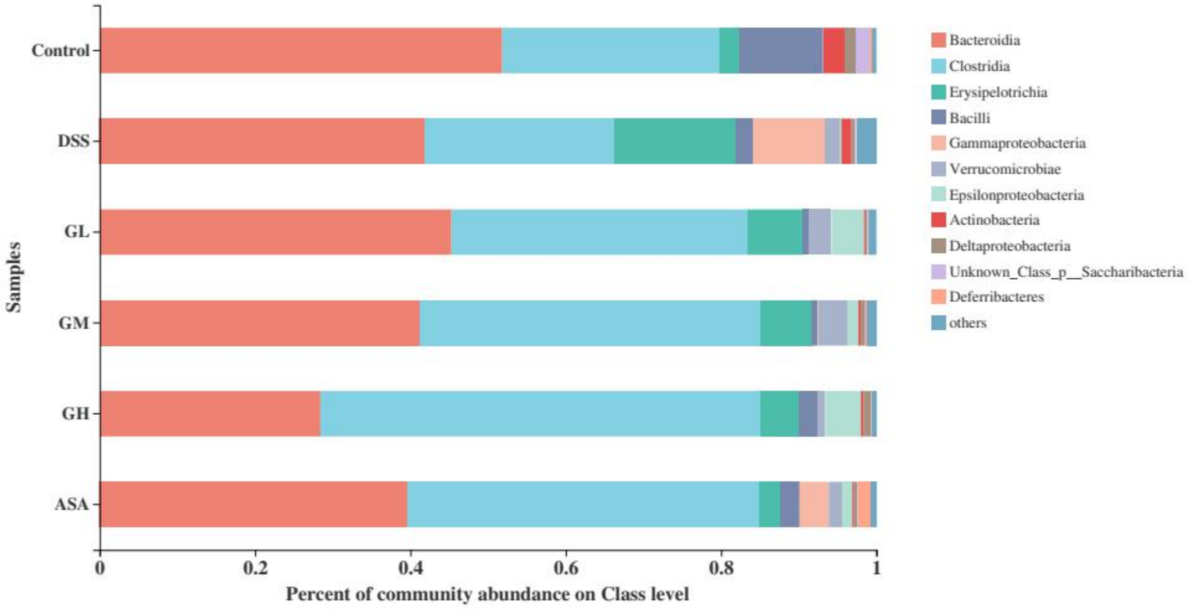

Control
DSS
GL
GM
GH
ASA
Samples
0
0.2
0.4
0.6
0.8
1
Percent of community abundance on Class level
Bacteroidia
Clostridia
Erysipelotrichia
Bacilli
Gammaproteobacteria
Verrucomicrobiae
Epsilonproteobacteria
Actinobacteria
Deltaproteobacteria
Unknown_Class_p__Saccharibacteria
Deferribacteres
others

B

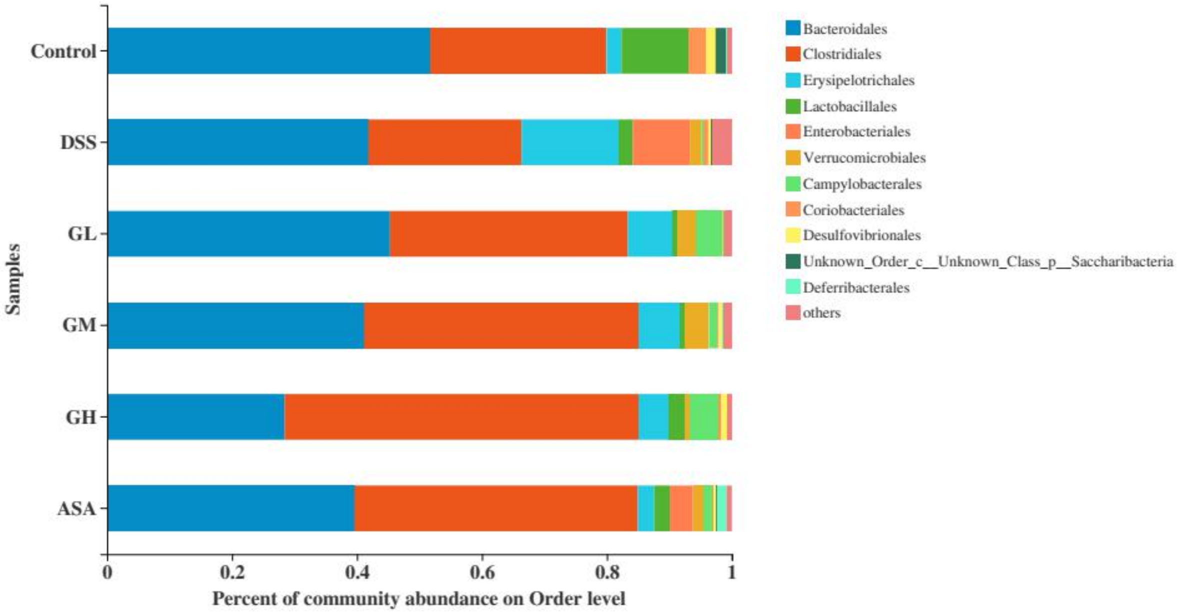

Control
DSS
GL
GM
GH
ASA
Samples
0
0.2
0.4
0.6
0.8
1
Percent of community abundance on Order level
Bacteroidales
Clostridiales
Erysipelotrichales
Lactobacillales
Enterobacteriales
Verrucomicrobiales
Campylobacterales
Coriobacteriales
Desulfovibrionales
Unknown_Order_c__Unknown_Class_p__Saccharibacteria
Deferribacterales
others

C

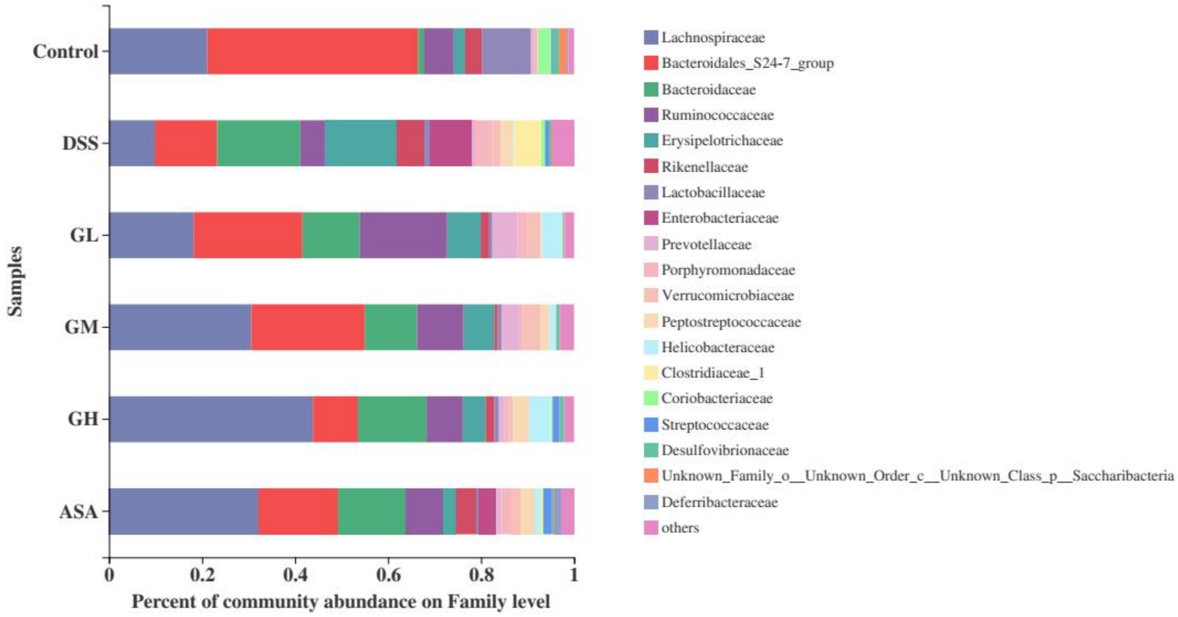

Supplementary Figure 4: Relative abundance of microbial community among the six groups at the (**A**) class, (**B**) order and (**C**) family level.

A

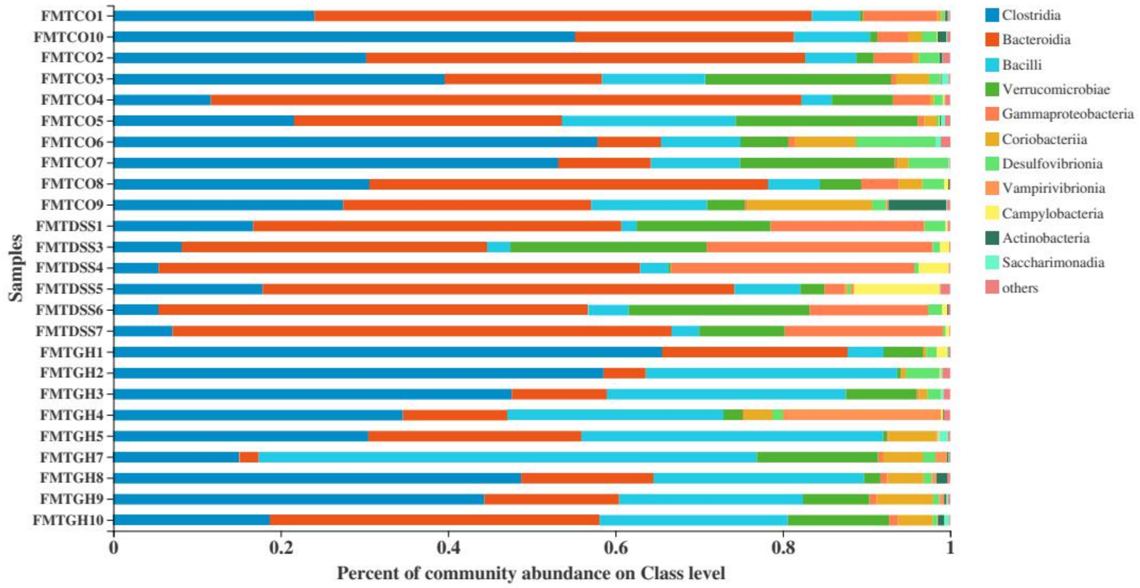
FMTCO1
FMTCO10
FMTCO2
FMTCO3
FMTCO4
FMTCO5
FMTCO6
FMTCO7
FMTCO8
FMTCO9
FMTDSS1
FMTDSS3
FMTDSS4
FMTDSS5
FMTDSS6
FMTDSS7
FMTGH1
FMTGH2
FMTGH3
FMTGH4
FMTGH5
FMTGH7
FMTGH8
FMTGH9
FMTGH10
Samples
0
0.2
0.4
0.6
0.8
1
Percent of community abundance on Class level
Clostridia
Bacteroidia
Bacilli
Verrucomicrobiae
Gammaproteobacteria
Coriobacteriia
Desulfovibrionia
Vampirivibrionia
Campylobacteria
Actinobacteria
Saccharimonadia
others

B

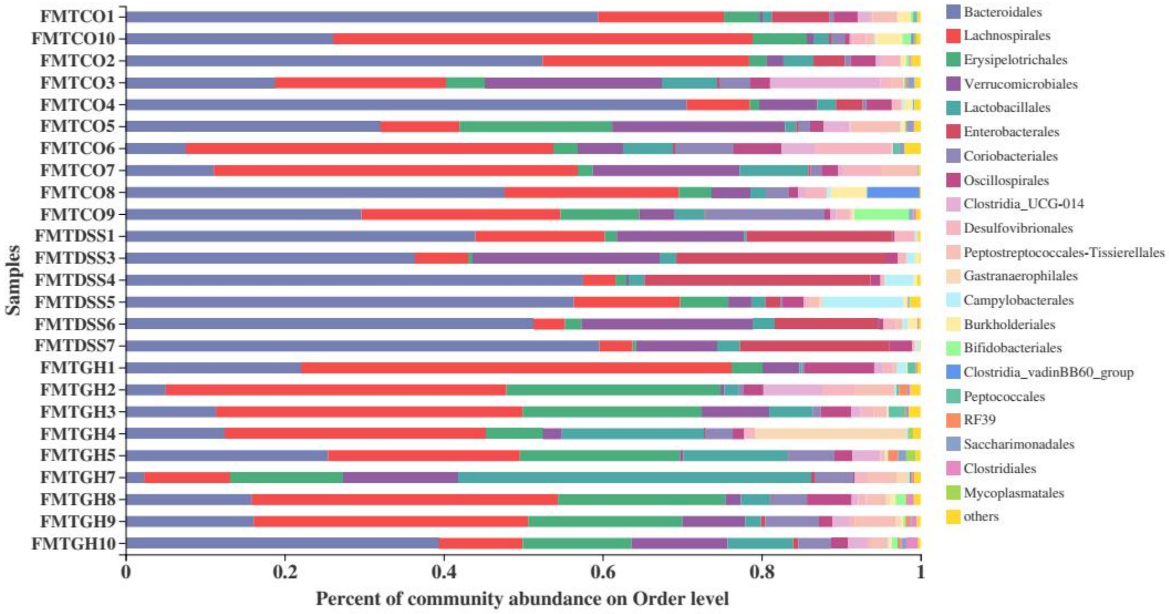
FMTCO1
FMTCO10
FMTCO2
FMTCO3
FMTCO4
FMTCO5
FMTCO6
FMTCO7
FMTCO8
FMTCO9
FMTDSS1
FMTDSS3
FMTDSS4
FMTDSS5
FMTDSS6
FMTDSS7
FMTGH1
FMTGH2
FMTGH3
FMTGH4
FMTGH5
FMTGH7
FMTGH8
FMTGH9
FMTGH10
Samples
0
0.2
0.4
0.6
0.8
1
Percent of community abundance on Order level
Bacteroidales
Lachnospirales
Erysipelotrichales
Verrucomicrobiales
Lactobacillales
Enterobacterales
Coriobacteriales
Oscillospirales
Clostridia_UCG-014
Desulfovibrionales
Peptostreptococcales-Tissierellales
Gastranaerophilales
Campylobacterales
Burkholderiales
Bifidobacteriales
Clostridia_vadinBB60_group
Peptococcales
RF39
Saccharimonadales
Clostridiales
Mycoplasmatales
others

C

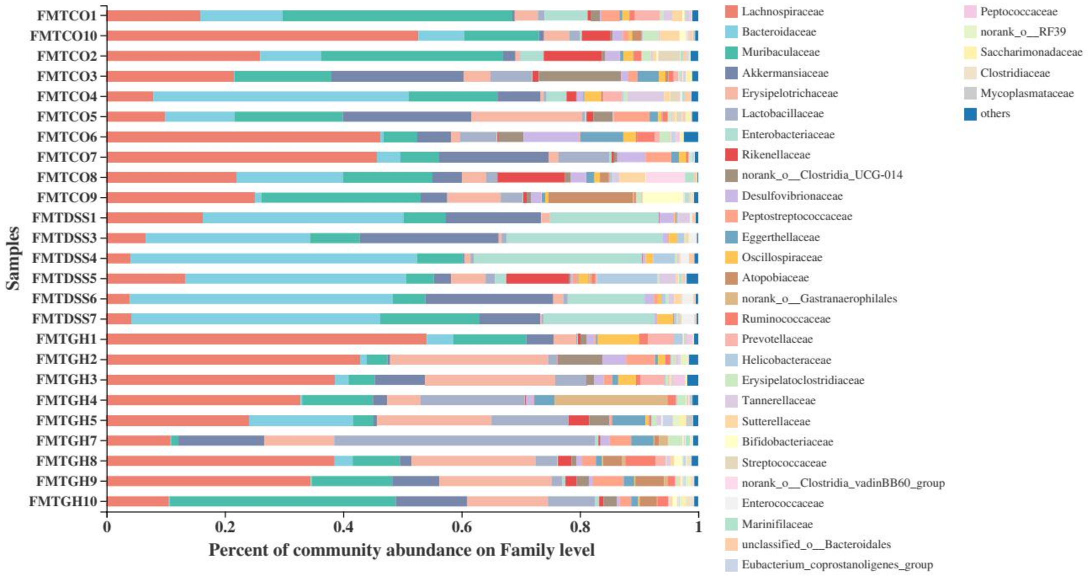

Supplementary Figure 5: Relative abundance of microbial community among the FMT groups at the (**A**) class, (**B**) order and (**C**) family level.
